# Supplementary material for: Exploratory Systematic Review of Mixed Martial Arts: An Overview of Performance of Importance Factors with over 20,000 Athletes
Source: Sports (Basel). 2022 May 24;10(6):80. doi: 10.3390/sports10060080 (PMC9227211; doi:10.3390/sports10060080)
Supplement: Supplementary file 1 [file sports-10-00080-s001.zip › Table S1 risk bias.pdf]

Table S1: Risk of bias assessment criteria

**Criteria**		**Definition**	**Scoring**		
			**0**	**1**	**2**
A	Peer reviewed	Study published in peer-reviewed journal	No	Yes	-
B	Number of participants	Number of participants included in study findings	<5	6-30	>31
C	Population defined	Age, sex, sport, time experience (or level) was described	No	Partly	Yes
D	Experimental design	Experimental design of the study period was described and replicable	No	Partly	Yes
E	MMA athlete information	MMA athlete parameters were described	No	Yes	-
