# Supplementary material for: Exploratory Systematic Review of Mixed Martial Arts: An Overview of Performance of Importance Factors with over 20,000 Athletes
Source: Sports (Basel). 2022 May 24;10(6):80. doi: 10.3390/sports10060080 (PMC9227211; doi:10.3390/sports10060080)
Supplement: Supplementary file 1 [file sports-10-00080-s001.zip › Table S2 application riskbias.pdf]

Table S2: Risk of bias assessment criteria and application in the studies

Criteria		Definition	Scoring		
			0	1	2
A	Peer reviewed	Study published in peer-reviewed journal	No	Yes	-
B	Number of participants	Number of participants included in study findings	<5	6-30	>31
C	Population defined	Age, gender, sport, time experience (or level) was described	No	Partly	Yes
D	Experimental design	Experimental design of the study period was described and replicable	No	Partly	Yes
E	MMA athlete information	MMA athlete parameters were described	No	Yes	-

Study	A	B	C	D	E	TOTAL
Alves et al., 2018	1	2	2	2	1	8
Amtmann et al., 2004	1	1	1	1	1	5
Amtmann et al., 2008	1	1	1	1	1	5
Andreato et al., 2014	1	1	1	1	1	5
Antoniettô et al., 2019	1	2	1	2	1	7
Azevedo et al., 2019	1	1	2	2	1	7
Aung et al., 2021	1	2	1	1	1	6
Barley et al., 2018[a]	1	2	2	1	1	7
Barley et al., 2018[b]	1	2	2	2	1	8
Barley et al., 2018[c]	1	2	2	1	1	7
Bernick et al., 2021	1	1	1	1	1	5
Buse et al., 2006	1	2	1	2	1	7
Bledsoe et al., 2006	1	2	1	1	0	5

Bodden et al., 2015	1	1	2	1	1	6
Brandt et al., 2018	1	1	2	2	1	7
Bray et al., 2021	1	2	2	2	1	8
Brechney et al., 2019	1	2	2	2	1	8
Brito et al., 2018	1	2	1	2	1	7
Camarço et al., 2016	1	0	2	2	1	6
Chen & Cheesman., 2013	1	2	1	2	1	7
Cherepkova et al., 2019	1	2	2	2	1	8
Chernozub et al., 2018	1	1	2	2	1	7
Chernozub et al., 2019	1	2	1	1	1	6
Connor & Egan., 2019	1	1	1	1	1	5
Connor, Shelley & Egan., 2020	1	1	2	2	1	7
Connor & Egan., 2021	1	1	2	2	1	7
Coswig et al., 2015	1	1	2	1	1	6
Coswig et al., 2016[a]	1	1	1	2	1	6
Coswig et al., 2016[b]	1	1	1	2	1	6
Coswig et al., 2019	1	1	2	2	1	7
Curran-Sills & Abedin, 2018	1	2	2	2	1	8
Dal Bello et al., 2019	1	2	1	2	1	7
De Souza et al., 2017	1	1	2	1	1	6
Del Vecchio et al., 2011	1	2	2	2	1	8
Dixson et al., 2018	1	2	2	1	1	7
Dos Santos et al., 2018	1	2	1	1	1	6
Fares et al., 2019	1	2	2	2	1	8
Fares et al., 2021[a]	1	2	1	2	1	7

Fares et al., 2021[b]	1	2	2	2	1	8
Faro, Lima-Junior & Machado., 2021	1	2	1	2	1	7
Fernandes et al., 2018	1	2	2	2	1	8
Follmer et al., 2019	1	2	1	1	1	6
Follmer, Andreato & Coswig., 2021	1	2	1	2	1	7
Fliotsos et al., 2021	1	2	1	2	1	7
Ghoul et al., 2019	1	1	2	2	1	7
Giboin & Gruber, 2019	1	1	2	2	1	7
Gomes-Santos et al., 2022	1	1	2	1	1	6
Heath & Callahan, 2013	1	2	2	1	1	7
Hillier et al., 2019	1	2	2	2	1	8
Hubbard et al., 2019	1	2	2	2	1	8
Hutchison et al., 2014	1	2	1	2	1	7
Ignatjeva et al., 2021	1	2	1	2	1	7
James et al., 2017[a]	1	2	2	2	1	8
James et al., 2017[b]	1	1	2	2	1	7
James et al., 2018	1	2	2	2	1	8
James et al., 2020	1	1	2	2	1	7
Jansen et al., 2021	1	1	1	1	1	5
Jetton et al., 2013	1	2	2	2	1	8
Ji, 2016	1	2	2	1	1	7
Kasper et al., 2019	1	0	2	1	1	5
Kirk et al., 2015	1	1	2	2	1	7
Kirk et al., 2021	1	1	2	1	1	6

Kostikiadis et al., 2018	1	1	2	2	1	7
Khatib et al., 2021	1	2	1	1	1	6
Kingery et al., 2021	1	2	2	2	1	8
Landers et al., 2017	1	1	2	2	1	7
LaRocca et al., 2019	1	2	2	2	1	8
Lindsay et al., 2015	1	1	1	2	1	6
Lindsay et al., 2017	1	1	1	2	1	6
Little et al., 2015	1	2	2	2	1	8
Marinho et al., 2016	1	1	2	2	1	7
Matuk et al., 2021	1	1	1	1	1	5
Massey et al., 2013	1	1	1	2	1	6
Matthews & Nicholas, 2017	1	1	1	2	1	6
Mayer et al., 2015	1	1	1	2	1	6
Mayer et al., 2017	1	1	2	2	1	7
McClain et al., 2014	1	2	1	1	1	6
Miarka et al., 2015	1	2	1	1	1	6
Miarka et al., 2016[a]	1	2	1	2	1	7
Miarka et al., 2016[b]	1	2	2	2	1	8
Miarka et al., 2016[c]	1	2	1	2	1	7
Miarka et al., 2017[a]	1	2	1	2	1	7
Miarka et al., 2017[b]	1	2	1	2	1	7
Miarka et al., 2018[a]	1	2	2	2	1	8
Miarka et al., 2018[b]	1	2	2	2	1	8
Miarka et al., 2019[a]	1	2	1	2	1	7
Miarka et al., 2019[b]	1	2	2	2	1	8

Miarka et al., 2021	1	1	1	1	1	5
Murugappan et al., 2021	1	1	1	1	1	5
Ngai et al., 2008	1	2	1	2	1	7
Niewczas et al., 2021	1	2	2	1	1	7
Park et al., 2019	1	2	2	1	1	7
Pavelka et al., 2020	1	2	2	2	1	8
Peacock et al., 2018[a]	1	1	2	2	1	7
[i]Peacock et al., 2018[b]	1	1	1	0	1	4
Petrou et al., 2021	1	2	1	1	1	6
Plush et al., 2021	1	2	1	1	1	6
Rahmani et al., 2017	1	1	1	1	1	5
Rainey., 2009	1	2	2	2	1	8
Ruiz-Barquín et al., 2019	1	2	1	1	1	6
Richardson et al., 2021	1	1	1	1	1	5
Santos-Junior et al., 2020	1	2	1	1	1	6
Shin et al., 2014	1	2	1	1	1	6
Sinnett, Maglinti & Kingstone., 2018	1	2	2	2	1	8
Stephenson & Rossheim, 2018	1	2	1	2	1	7
Schild & Zettler., 2021	1	2	1	1	1	6
Stellpflug, Menton & LeFevere.., 2020	1	1	1	2	1	6
Tabben et al., 2018	1	1	2	2	1	7
Tiernan et al., 2021	1	1	1	1	1	5
Tota et al., 2019	1	1	2	2	1	7
Wiechmann et al., 2016	1	1	2	2	1	7

Zebrowska et al., 2017	1	2	1	2	1	7

[i] Peacock, C. A., Sanders, G. J., Antonio, J., & Silver, T. A. (2018). The reporting of a multifaceted mixed martial arts strength and conditioning program. *Journal of Exercise Physiology Online*, *21*(1), 86-91.
