# Supplementary material for: Exploratory Systematic Review of Mixed Martial Arts: An Overview of Performance of Importance Factors with over 20,000 Athletes
Source: Sports (Basel). 2022 May 24;10(6):80. doi: 10.3390/sports10060080 (PMC9227211; doi:10.3390/sports10060080)
Supplement: Supplementary file 1 [file sports-10-00080-s001.zip › Table S3 Main findings regarding studies.pdf]

Table S3 (n = 112 studies)

Main findings regarding injuries in MMA athletes (n = 29)

Study	Sex	n (M/F)	Age (Years)	Competitive Level	Main Findings
Buse., (2006)	Male	1284	29 ± 5	Professional	Of the 642 matches, 182 (28 ±3%) were stopped because of head impact, 106 (17±3%) because of musculoskeletal stress, 91 (14±3%) because of neck choke, 83 (13±3%) because of miscellaneous trauma, 173 (27±3%) because of expiration of match time, and seven (1±1%) because of disqualification. ± 95% confidence interval. Conclusions: Blunt force to the head resulted in the highest proportion of match stoppages. Further research is warranted to delineate the morbidity associated with participation in mixed martial arts.
Bledsoe et al., (2006)	Male	220	29 ± 5 range from 19 to 44 years	Professional	A total of 171 MMA matches involving 220 different fighters occurred during the study period. There were a total of 96 injuries to 78 fighters. Of the 171 matches fought, 69 (40%) ended with at least one injured fighter. The overall injury rate was 28.6 injuries per 100 fight participations or 12.5 injuries per 100 competitor rounds. Facial laceration was the most common injury accounting for 47.9% of all injuries, followed by hand injury (14%), nose injury (10%), and eye injury (8%). With adjustment for weight and match outcome, older age was associated with significantly increased risk of injury. The most common conclusion to an MMA fight was a technical knockout (TKO) followed by a tap out. The injury rate in MMA competitions is compatible with other combat sports involving striking. The lower knockout rates in MMA compared to boxing may help prevent brain

					injury in MMA events. Injury rate in MMA competitions is similar to other combat sports.
Bray et al., (2021)	Male and female	91/ 12	32.87 ± 4.62	Professional	the current longitudinal study found temporal trends in structural, cognitive, and neuropsychiatric outcomes that differed by weight class and type of combat sport. These findings may suggest that, although weight class was not previ- ously found to be a determinant of baseline brain health among professional fighters, it alters the course of neurodegenerative changes over time as RHIs are accumulated. Previously reported null findings from the PFBHS regarding baseline neuroimaging and neu- ropsychiatric outcomes should be interpreted conservatively given the smaller sample size.
Bernick et al., (2021)	Male	30	N/A	Professional	the fighter that sustained the first concussion in a bout/match almost always went on to lose. This finding may be due to one or more of the following: sustaining a concussion makes one more susceptible to another, impairs ability to compete as well, or that the inferior fighter is most likely to be concussed first.
Curran-Sills & Abedin et al., (2018)	Male	686	27 ± 6	Amateur and professional	The injury rate per 100 athlete exposure was 23.6 (95% CI 20.5 to 27.0), the injury rate per 100 min of exposure was 4.1 (95% CI 3.48 to 4.70) and the concussion rate per 100 athlete exposure was 14.7 (95% CI 11.8 to 17.2). The most common location of injury was the head and mild traumatic brain injury (mTBI) mTBI was the most common type of injury.
Fares et al., (2019)	Male and female	249/36	N/A	Amateur and professional	A total of 291 injuries were recorded in 285 fights from nine weight divisions. The overall injury rate was 51 per 100 athletic exposures (AE). Males predominantly partook in 249 matches (87%) and had higher injury rates (54 injuries per 100 AE) than females (30 injuries per 100 AE). Decision was the most common way a match ended. Knockouts (KOs) were significantly higher in males (36%) than in females (14%, P

					= 0.0007). Submissions were significantly higher in females (36%) than in males (16%, P = 0.001). Head injuries (67%) were the most common injuries reported with a rate of 34 per 100 AE. Upper limb injuries were significantly higher in females (40%) than in males (14%, P = 0.0003). Lower limb injuries were significantly higher in males (19%) than in females (5%, P = 0.01). Head injuries were significantly associated with KOs (P < 0.0001). Upper limb injuries (P = 0.032) and lower limb injuries (P = 0.034) were significantly associated with matches that ended with Decision. Trend-line analyses showed that as weight division increases, overall injury rates, head injuries, lower limb injuries, and KOs' frequency increase, whereas upper limb injuries, Submission frequency, and Decision frequency decrease. MMA has a high injury rate. Gender, way of finish, and weight play an important role in predicting fight outcomes and injury profiles. Injury prevention policies must be entertained to limit injury risk in MMA.
Fares et al., (2021)[a]	Male and Female	816	N/A	Professional	The UFC recorded a head injury rate of 35 injuries per 100AE over the studied period. This is higher than the injury rate reported by Thomas RE and Thomas BC; probably due to their inclusion of other promotions that exhibit lower injury rates. Male fighters had a higher head injury rate when com- pared to females, and the most common form of injury was TBI. Injuries occurred more often in fights that ended via TKO/ KO, and the rate was generally higher in heavier weight divisions.
Follmer et al., (2019)	Male and Female	1728/175	N/A	Professional	The combined KO/TKO rates per 100 athlete-exposures in the middleweight (19.53), light heavyweight (20.8), and heavyweight (26.09) divisions were greater than previously reported for MMA. While stoppage via KO/TKO occurred in 8% of combats in the female strawweight division, it occurred in 52% of the male heavyweight fights. The male middleweight (P = 0.001), light heavyweight (P < 0.001),

					and heavyweight divisions (P < 0.001) had an increased risk of KO/TKO due to strikes to the head by 80%, 100%, and 206%, respectively. The risk in the flyweight division decreased 62% (P = 0.001). All categories were compared with the lightweight division. The female bantamweight category presented a 221% increased risk in matches ending due to KO/TKO compared with the strawweight division (P = 0.012). Punches to the head were the major technique used to end a combat via KO/TKO, regardless of sex and weight class.
Fliotsos et al., (2021)	N/A	4416	N/A	Professional	Ophthalmic injuries in professional MMA were prevalent, were most often lacerations surrounding the eye, and often accompanied the fighter losing their match.
Heath & Callahan., (2013)	Male and female	112/7	27 ± 7	N/R	15% of the MMA athletes reported experiencing a KO, and nearly one-third reported a TKO. 13% of athletes who reported experiencing a concussive-like incident over the course of their training. 60% of the athletes returning in 2 days or less. KO history with the amount of time they sparred during the week r(16) = .51, p = .04. concussive symptoms on the Post-Concussion Scale (PCS) after a KO, TKO: headaches (M = 2.0±1.7), fatigue (M = 1.7±1.8), and dizziness (M = 1.3±1.5), all of which were in the mild range.
Hubbard et al., (2019)	Male and female	86/7	M: 28 ± 7<br>F: 27 ± 1	Regional	MMA athletes (28 ± 7 years, n = 92) underwent K-D testing prior to and following a workout or match. Comparison of baseline and post-workout/match K-D times to assess any significant change. K-D tests worsened (longer) in a majority of athletes following an 'event' (N = 21) (49.6 ± 7.8 s vs 46.6 ± 7.8 s, p = 0.0156, Wilcoxon signed-rank test). K-D tests improved (shorter) following a standard workout or match in which no 'event' occurred in a majority of cases (n = 69) (44.2 ± 7.2 s vs 49.2 ± 10.9 s, p = <0.0001, Wilcoxon signed-rank test). Longer duration (worsening) of post- match K-D tests occurred in most athletes

					sustaining an 'event'; K-D tests shortened (improved) in a majority of athletes not sustaining an 'event'. Our study suggests MMA athletes suffering an 'event' may have
Hutchison et al., (2014)	Male	844	30 ± 4	Professional	Rates of KOs and TKOs in MMA are higher than previously reported rates in other combative and contact sports.
Jansen et at., (2021)	Male and female	16/4	18 to 40 years	Amateur	Characteristic profiles of head impact exposure differed between boxing and MMA athletes; however, the impact magnitudes were not significantly different for male and female athletes.
Ji., (2016)	Male and female	438/17	28 ± 8	Regional	The main locations of injuries during MMA were the upper extremities, neck, and head. The injury patterns in MMA are generally considered similar to those in other combat sports. However, as MMA includes various types of martial arts, the risk of injury is higher in MMA than in other combat sports.
Landers et al., (2017)	Male	234	29 ± 5	Professional	The mean static visual acuity testing logMAR was -0.173 (standard deviation [SD] = 0.114). Mean dynamic visual acuity test values decreased with head movement to 0.196 logMAR (SD = 0.103) in yaw; $p < 0.001$, and to 0.283 logMAR (SD = 0.133) in pitch; $p < 0.001$. MMA fighters had a decay, beyond normal ranges, in visual acuity during head movement. These decreases may suggest vestibulo-ocular reflex impairment and were unrelated to self-reported concussion history. These results should be cautiously interpreted since.
LaRocca et al., (2019)		40/2	26.5 ± 5.8		Traumatic brain injury (TBI) is a major cause of death and disability worldwide, with mild TBI (mTBI) accounting for 85% of cases. We identified a subset of salivary and serum miRNAs that showed robust utility at predicting TBI likelihood and demonstrated quantitative associations with head impacts as well as cognitive and balance measures. In contrast, serum proteins demonstrated far less utility. We

					also found that the timing of the responses varies in saliva and serum, which is a critical observation for biomarker studies to consider.
Mayer et al., (2015)	Male and Female	11/2	28 ± 5	Regional	MMA athletes self-reported a higher incidence of previous concussions and significantly more cognitive symptoms during concussion recovery. Fighters also exhibited reduced memory and processing speed relative to controls on neuropsychological testing coupled with cortical thinning in the left posterior cingulate gyrus and right occipital cortex at baseline assessment. Over a 1-year follow-up period, the athletes experienced a significant decrease in both white matter volume and N-acetyl aspartate concentration, as well as relative thinning in the left middle and superior frontal gyri.
Mayer et al., (2017)	Male and Female	11/2	28 ± 5	Regional	Although diffusion magnetic resonance imaging (dMRI) has been widely used to characterize the effects of repetitive mild traumatic brain injury (rmTBI), to date no studies have investigated how novel geometric models of microstructure relate to more typical diffusion tensor imaging (DTI) sequences. Moreover, few studies have evaluated the sensitivity of different registration pipelines (non-linear, linear and tract-based spatial statistics) for detecting dMRI abnormalities in clinical populations. Results from single-subject analyses in healthy controls (HC) indicated a strong negative relationship between fractional anisotropy (FA) and orientation dispersion index (ODI) in both white and gray matter. Equally important, only moderate relationships existed between all other estimates of free/intracellular water volume fractions and more traditional DTI metrics (FA, mean, axial and radial diffusivity). These findings suggest that geometric measures provide differential information about the cellular microstructure relative to traditional DTI measures. Results also suggest greater sensitivity for non-linear registration pipelines that

					maximize the anatomical information available in T1-weighted images. Clinically, rmTBI resulted in a pattern of decreased FA and increased ODI, largely overlapping in space, in conjunction with increased intracellular and free water fractions, highlighting the potential role of edema following repeated head trauma. In summary, current results suggest that geometric models of diffusion can provide relatively unique information regarding potential mechanisms of pathology that contribute to long- term neurological damage.
McClain et al., (2014)	Male and female	1422	N/A	Amateur and professional	The overall injury rate was 9% of fight participations (121 injuries/1422 fight participations) or 6% of rounds (121/2178 rounds). Injury rates were similar between men and women, but a greater percentage of the injuries caused an altered mental state in men. The risk of being injured was significantly greater for bouts held in Kansas, at the professional level, lasting more rounds, and ending in a KO/TKO. Fighters also were more likely to be referred to the ER if they participated in longer bouts ending in a KO/TKO. The observed injury rate was lower than previously reported suggesting recent regulatory changes have made MMA a safer sport. Increased clinical awareness and additional research should be extended to head-related injuries in MMAs especially those associated with KOs/TKOs.
Miarka et al., (2018)[b]	Male and female	41/41	M: 32 ± 5,4<br>F: 32 ± 3	Professional	Male middleweights and male lightweights showed longer durations of groundwork and standing actions than female middleweights and male lightweights. In standing and groundwork actions, male lightweights presented longer time between striking actions than female middleweights.
Matuk et al., (2021)	Male and female	5/3	18–30	Professional	When selecting fighter samples for gene analysis, athletes who had more cumulative hits in longer fights (decision or later round matches) and more severe mecha- nisms and methods of injury (knockout or

					technical knockout by punches or kicks) would have higher genetic expression than those who had shorter bouts or less hits. Using this standard technique, we noted the highest ratios in fighters sustaining longer fights and those who sustained a technical knockout. Conversely, we noted the lowest ratios for fighters who fought the least amount of time and those who won matches in earlier rounds. This result was particularly evident at the key seven gene level and suggests a pattern of higher genetic signal associated with mechanism of injury.
Ngai et al., (2008)	NR	636	NR	Professional	Injury rate was 23.6 per 100 fight participations. Most common reported injuries were lacerations and upper limb injuries. Severe concussion rate was 15.4 per 1000 athlete exposures, or 3% of all matches.
Rahmani et al., (2017)	Male and female	18/2	N/A	Professional	The majority of injuries were sustained during competition (81%) with the remainder of injuries sustained during training. A fracture of the mid-shaft of the ulna being the most common. In cases where the injury was sustained during competition, 6/17 fights ended in victory for the injured athlete.
Rainey., (2009)	Male and female	52/3	18-39	Amateur and professional	Two hundred seven injuries were reported in the study. Low belt ranks had significantly more injuries more than any other belt rank, resulting in more than two times higher injury rate. Professional fighters had significantly more injuries than amateur fighters, resulting in three times higher injury rate. The most common body region injured was the head/neck/face (38%), followed by the lower extremities (30%), upper extremities (23%), torso (8%), and groin (1%). Injuries to the nose (6%), shoulder (6%), and toe (6%) were the most common. The most common type of injury was contusions (29%), followed by strains (16%), sprains (15%), and abrasions (10%).Injury prevention efforts should consider the prevalence and distribution of injuries and focus on reducing or preventing injuries to the head/neck/face in MMA

					related activities. Preventative measures should focus on improving protective equipment during training, and possible competition rule modifications to further minimize participant injury.
Shin et al., (2014)	Male	81	28 ± 5	Professional	A total of 74 boxers and 81 mixed martial arts fighters were included in the analysis and scanned by use of DTI. Individual information and data on fight exposures, including number of fights and knockouts, were collected. A multiple hierarchical linear regression model was used in region-of-interest analysis to test the hypothesis that fight-related exposure could predict DTI values separately in boxers and mixed martial arts fighters. Age, weight, and years of education were controlled to ensure that these factors would not account for the hypothesized effects. We found that the number of knockouts among boxers predicted increased longitudinal diffusivity and transversal diffusivity in white matter and subcortical gray matter regions, including corpus callosum, isthmus cingulate, pericalcarine, praecuneus, and amygdala, leading to increased mean diffusivity and decreased fractional anisotropy in the corresponding regions. The mixed martial arts fighters had increased transversal diffusivity in the posterior cingulate. The number of fights did not predict any DTI measures in either group. These findings suggest that the history of fight exposure in a fighter population can be used to predict microstructural brain damage.
Stepheson & Rossheim., (2018)	Male	409	25	Amateur	Abrasions/contusions were the most commonly diagnosed MMA injury. Being struck resulted in the majority of injuries. The head was the most injured body region. Most MMA injuries occurred during competition.

Tiernan et al., (2021)	Male	12	N/R	professional or semi-professional and one amateur	Head acceleration data were recorded from 451 video confirmed impacts in MMA at 30 events. This is the only known study to measure head accelerations in vivo that have resulted in a concussive injury in an unhelmeted sport. The impact with the highest resultant angular acceleration from each event was simulated using the GHBMC head model. Linear accelerations to the side of the head (Y-direction) were 62.2% higher in impacts that resulted in a concussive injury. Punches to the jaw create high lateral accelerations and due to the offset from the coordinate system this leads to high angular accelerations about the X-axis (coronal plane).
Khatib et al., (2021)	Male	60	N/R	Professional	Punches were the most landed event types for both weight classes. This is likely because MMA fights start standing, and punches can be thrown at a variety of angles, velocities, and distances. Moreover, punches can be strung together in combinations, making them an ideal attack to inflict damage on an opponent. This finding is consistent with previous research reporting that the majority of KO and non-KO head strikes MMA fights came from punches to the head. The LW were targeting the mandibular region for the KO leading to high-category strains, whereas the added mass and strength of the HW fighters led to a trend of higher frequency of very high strains from punches, elbows, and knees. Impacts to the mandible are an effective location for fighters to induce high rotational accelerations to induce loss of conscious- ness. These results indicate that LW and HW fighters engage in MMA in unique ways, creating differences in brain trauma profiles.
Kingery et al., (2021)	Male and female	438/16	30.0 ±3.9	Professional	there was a high rate of return to professional sport and no evidence of an associated decline in performance following major injury requiring withdrawal from a fight card. Older age at the time of injury was

					associated with decreased odds of being able to return to professional fighting.

M: male; F: female. N/A: not available. * Mean considering male and female; NA: Not reported

Main findings regarding weight loss in MMA athletes (n = 21)

Study	Sex	n (M/F)	Age (Years)	Competitive Level	Main Findings
Alves et al., (2018)	Male	12	20 ± 1	Amateur	Decreased BM and handgrip strength, and increased urine density from baseline to match time, indicating that athletes could not fully restore physiological function within the 24 hrs between the official weigh-in and competition time. This indicates that the negative effects of RWL on physiological function are not fully regained in the 24-hr period between the official weigh-in and the start of a bout.
Andreato et al., (2014)	Male	8	22 ± 5	Professional	The results showed that all participants already had lost weight to take part in another event. The athletes reported that they always adopted as method to lose weight: to make more exercise (50 %) and reduce food intake of liquids (37.5 %). The dangers methods to health were cited by athletes in the following sequence: use of diuretics (37.5%), sauna (37.5%), training intentionally in heated training rooms (50 %) and use of diet pills (12.5%). In the competition analyzed 88% of athletes reduced their body mass (4.5 ± 4.2 kg, 5.4 ± 4.3 %) to compete. The salivary osmolality did not show significant difference between weigh- in (55.6 ± 30.7 mOsmol/kg H2O) and pre-match moment (40.2 ± 27.9 mOsmol/kg H2 O). The profile of mood states did not change between the weigh-in (tension: 1.2 ± 1.8, depression: 0 ± 0, anger: 1.2 ± 1.6, vigour: 14.0 ± 0.7, fatigue: 2.6 ± 5.3, confusion: 0.2 ± 0.4) and the pre-match moment (tension: 2.6 ± 2.2, depression: 0 ± 0, anger: 1.4 ± 1.9, vigour: 15.6 ± 0.5, fatigue: 0.0, confusion: 2.8 ± 3.9).

					Conclusions. The conclusion is that there is a great prevalence of weight loss in MMA athletes in this sample. Furthermore, dangers methods to health are or were practiced to reduce the body mass to compete.
Barley et al., (2018)[a]	Male and female	70	27 ± 6	Amateur to professional	The majority (≥85%) of all groups reported using a weight loss strategy prior to competition. 95% of athletes cut weight for competition, with 9.8 ± 7.9 kg lost on average over 27 ± 24 days. Specifically, MMA athletes reported loosing 12% of their body mass 14 days before the fight (~7% of body mass) and another more 24-hours before the official weight-in (~4% of body mass). Gradual dieting, increased quantities of exercise, sauna, restricting fluid ingestion, training in plastic/rubber suits, training in heated rooms, skipping meals, and water loading were the main methods reported for MMA athletes to lose weight.
Barley et al., (2018)[b]	Male	14	23 ± 4	Amateur	Mixed martial arts athletes completed in a randomized counterbalanced order a dehydration protocol, (DHY: 3-hour cycling at 60 W in 408 C to induce 5% dehydration) or thermoneutral control (258 C: CONT) exercise, followed by ad libitum fluid/food intake. Body mass was reduced (4.8 ± 0.8%) after DHY (p, 0.001) and remained lower than CONT at 3 hours and 24 hours after DHY ($p = 0.003$ and $p = 0.024$, respectively). Compared with CONT, average sled push times were slower 3 hours and 24 hours after DHY (19 ± 15%; $p = 0.001$; $g = 1.229$ and 14 ± 15%; $p = 0.012$; $g = 0.671$, respectively). When compared with the CONT, handgrip was weaker 3 hours after DHY (53 ± 8 and 51 ± 8 kg; $p = 0.044$, $g = 0.243$, respectively) and medicine ball chest throw distances were shorter 24 hours after DHY (474 ± 52 and 449 ± 44 cm; $p = 0.016$, $g = 0.253$, respectively). No significant differences were observed in vertical jump ($p = 0.467$). Urine specific

					gravity was higher than CONT 20 minutes (p = 0.035) and 24 hours (p = 0.035) after DHY. Acute dehydration of 4.8% body mass results in reduced physical performance 3 and 24 hours after DHY. There is need for caution when athletes use dehydration for weight loss 24 hours before competition.
Barley et al., (2018)[c]	Male	14	25 ± 4	Amateur	Acute dehydration of ~3% body mass followed by 3 h of recovery impaired muscular strength-endurance and increases fatigue perception without changes in markers of central or peripheral function.
Brandt et al., (2018)	Male	12	28 ± 9	Professional	RWL associated with reporting higher confusion, greater total mood disturbance, and high anger at the official weigh-in. However, RWL did not have deleterious effects on performance.
Brechney et al., (2019)	Male and female	73/2	N/A	Amateur and professional	This study aimed to identify whether fight outcome (win vs. loss vs. type of loss) was influenced by magnitudes of body mass (BM) lost through weight cutting and BM regained before the fight after official weigh-in in amateur and professional MMA athletes with previous weight-cutting experience. Body mass data were collected using self-report from 75 MMA athletes (59 amateur and 16 professional) before commencing weight-cutting practices 7 days before weigh-in, by the regulating body at their official weigh-in 24 hours before the fight and through direct measurement immediately before competition. Data were analysed according to win; loss by technical knockout or knockout (KO); loss by submission; or loss by the judge's decision. Athletes who lost their fight cut significantly more BM (10.6%) compared with athletes who won (8.6%) (p= 0.04, d= 0.48, 95% confidence interval [CI]= 0.02–0.93), but there were no differences between types of loss. There were no significant differences in recovered BM between athletes who won (6.8%) vs. lost (7.4%), or type

					of loss. Furthermore, there was a significant relationship between greater magnitudes of BM cut and greater likelihood of losing the fight (B= - 0.12, P= 0.048), odd ratio 0.89 (95% CI: 0.79–1.00). This study provides the first line of evidence that excessive weight cutting may be detrimental to fight outcome in MMA.
Camarço et al., (2016)	Male	2	22 ± 0	Professional	Recovery period between weigh-in and competition was insufficient for total reestablishment of salivary nitrate after RWL. Higher amounts of RWL had negative impacts on average power and cognition.
Connor & Egan (2019)	Male	30	25 ± 4	Amateur and professional	The aim of the present study was to describe self-reported methods of RWL in a sample of competitive MMA athletes comprising of both amateur and professional fighters. The previously-validated Rapid Weight Loss Questionnaire, with the addition of questions on water loading and hot salt baths, was completed anonymously online by athletes (n = 30; all male, n = 15/15 professional/amateur) from MMA clubs around Dublin, Ireland. All but one (97%) of the athletes surveyed lost weight in order to compete, with the average weight loss being 7.9% ± 3.1% of habitual body mass. The RWL score (mean ± SD) for this sample was 37.9 ± 9.6, and a tendency for higher [6.0 (95%CI; −1.1, 13.1) (p = 0.093; d = 0.64)] RWL scores for professional (40.8 ± 8.9) compared to amateur (34.8 ± 9.6) athletes was observed. Frequencies of "always" or "sometimes" were reported as 90% for water loading, 76% for hot salt baths and 55% for 24 h of fasting. Fellow fighters (41%) and coaches/mentors (38%) were "very influential" on RWL practices of these athletes, with doctors (67%), dietitians (41%), and physical trainers (37%) said to be "not influential". RWL is highly prevalent in MMA across both amateur and professional athletes, and RWL scores are higher than other

					combat sports. Water loading and hot salt baths are amongst the most commonly used methods of RWL despite little research on these methods for body mass reduction or effects on performance in weight category sports.
Connor, Shelley & Egan (2020)	Male	11	29 ± 5	Professional	The magnitude of body weight loss were similar for the protocols with and without salt on the bath. Body mass loss when bathing in a hot bath of fresh water (FWB) is similar to bathing in a hot bath with ~1.6% Epsom salt added (SWB).
Connor & Egan (2021)	Male	8	29.4 ± 5.3	Amateur and professional	Under the conditions employed, the magnitude of body mass lost in SWB was similar to water was used FWB. Augmenting passive fluid loss during hot water immersion with the addition of salt may require a higher salt concentration than that presently utilised.
Coswig et al., (2015)	Male	17	27 ± 5	Professional	Less glucose availability and increased muscle damage markers in the RWL group that weighed in 24 h before the fight compared with the non-weight loss group that weighed in on the day of the fight.
Coswig et al., (2019)	Male	15	25 ± 6	Professional	Results showed significant differences between the time points in terms of total caloric intake as well as carbohydrate, protein and lipid ingestion. Statistical differences in combat analysis were observed between the winners and losers in terms of high- intensity relative time [58 (10;98) s and 32 (1;60) s, respectively], lower limb sequences [3.5 (1.0;7.5) sequences and 1.0 (0.0;1.0) sequences, respectively], and ground and pound actions [2.5 (0.0;4.5) actions and 0.0 (0.0;0.5) actions, respectively], and logistic regression confirmed the importance of high-intensity relative time and lower limb sequences on MMA performance. RWL and WRG strategies were related to technical-tactical and time-motion patterns as well as match outcomes. Weight management should be carefully supervised by

					specialized professionals to reduce health risks and raise competitive performance
Faro, Lima-Junior & Machado et al., (2021)	Male and female	1296/104	N/R	Professional	The current study aimed to analyze the influence of RWG on fight success in professional MMA. The main finding was that %RWG was a predictor of fight success, independently of sex, BM division, and competition level. In fact, each 1% of additional RWG increased the probability of winning by 4.5%. Moreover, elite and national level athletes regain more BM than regional level MMA athletes. The absolute and relative RWGs were different among the BM divisions, with more pronounced differences for flyweight, bantamweight, featherweight, and lightweight divisions, especially for %RWG. Previous studies presented controversial findings regarding the influence of the magnitude of RWG on the fight outcome (Brechney et al., 2019; Coswig et al., 2019; Kirk et al., 2020). Our results provide robust evidence from a large-scale ecological study that RWG might boost the chances of winning in MMA athletes.
Hillier et al., (2019)	Male and female	287/27	M: 27 ± 5<br>F: 29 ± 5	Amateur and professional	The magnitude of RWL in one week prior to weigh-in was significantly greater for professional athletes compared to amateur level. In the 24 h preceding weigh-in, the magnitude of rapid weight loss was greater at professional than amateur level in men.
Jetton et al., (2013)	Male and female	38/2	25 ± 1	Amateur (> 1 year)	The magnitude of rapid weight loss in one week prior to weigh-in was significantly greater for professional athletes compared to those competing at amateur level (men: 5.9% v 4.2%; women: 5.0% v 2.1% of body weight; p<0.05). In the 24 h preceding weigh-in, the magnitude of rapid weight loss was greater at professional than amateur level in men (3.7% v 2.5% of body weight; p<0.05. Most athletes 'always' or 'sometimes' used water loading (72.9%), restricting fluid intake (71.3%) and sweat suits (55.4%) for rapid weight loss.

					Coaches were cited as the primary source of influence on rapid weight loss practices (men: 29.3%, women: 48.1%). There is a high reported prevalence of rapid weight loss in MMA, at professional and amateur levels. Our findings, constituting the largest enquiry to date, call for urgent action from MMA organisations to safeguard the health and wellbeing of athletes competing in this sport
Kasper et al., (2019)	Male	1	22	Professional	The athlete adhered to a "phased" weight loss plan consisting of 7 weeks of reduced energy (ranging from 1300 – 1900 kcal.d-1) intake (phase 1), 5 days of water loading with 8 L per day for 4 days followed by 250 ml on day 5 (phase 2), 20 h fasting and dehydration (phase 3) and 32 h of rehydration and refuelling prior to competition (phase 4). Body mass declined by 18.1 % (80.2 to 65.7 kg) corresponding to changes of 4.4, 2.8 and 7.3 kg in phase 1, 2 and 3, respectively. We observed clear indices of relative energy deficiency, as evidenced by reduced RMR (-331 kcal), inability to complete performance tests, alterations to endocrine hormones (testosterone: <3 nmol.L-1) and hypercholesterolemia (>6 mmol.L-1). Moreover, severe dehydration (reducing body mass by 9.3%) in the final 24 hours prior to weigh-in induced hypernatremia (plasma sodium: 148 mmol.L-1) and acute kidney injury (serum creatinine: 177 μmol.L-1). These data therefore support publicised reports of the harmful (and potentially fatal) effects of extreme weight cutting in MMA athletes and represent a call for action to governing bodies to safeguard the welfare of MMA athletes.
Matthews & Nicholas et al., (2017)	Male	7	25 ± 4	Amateur and professional	Body mass decreased significantly from baseline, and increased significantly during the regain period. At weigh-in, 57% of athletes were dehydrated and the remaining 43% were severely dehydrated.

					Athletes reported using harmful dehydration-based RWL strategies such as sauna (43%) and training in plastic suits (43%).
Murugappan et al., (2021)	Male and Female	455/57	N/A	Professional	In total, 512 professional mixed martial artists (455 males and 57 females) were included. Of these, 503 (98%) athletes gained body mass between weigh-in and their bouts. Total RWG between weigh-in and competition was 5.5 ± 2.5 kg, corresponding to an 8.1% ± 3.6% body mass increase. Total RWG was 5.6 ± 2.5 kg (8.1% ± 3.6%) for males and 4.5 ± 2.3 kg (8.0% ± 3.8%) for females. More than one quarter of men and one third of women gained >10% body mass between weigh-in and competition. Athletes from leading international promotions gained more absolute, but not relative, body mass than those from regional promotions. Our findings indicate RWG is nearly ubiquitous in professional , with a similar prevalence in male and female athletes. Trends based on promotion suggest a larger magnitude of RWG in presumably more experienced and/or successful mixed martial artists from leading international promotions
Park et al., (2019)	Male	92	N/A	Professional	When comparing the number of methods of weight cutting with the source of advice, those who received their advice from social media used slightly more methods of weight cutting (M= 4.86, SD = 1.27) than those who did not (M= 4.02, SD = 1.55); t(90) = − 2.53, p < .05. MMA athletes that used the help of a registered dietitian nutritionist also reported using the least amount of methods for weight-cutting than any other category (M= 3.84, SD = 1.67). Those that used teammates and did not use a registered dietitian nutritionist used slightly more methods (M= 4.46, SD = 1.41) than those who used a registered dietitian nutritionist. The findings of this study report that professional MMA athletes do undergo rapid weight loss through various methods to make weight for competition. This study adds

					evidence to the literature that most professional MMA athletes undergo RWL for competition without the guidance of a registered dietitian nutritionist. It is unclear what the effect of using a registered dietitian nutritionist may have on an MMA athletes' ability to reduce weight in a safe and effective manner. Future research should seek to investigate if employing a registered dietitian nutritionist may lead to a higher rate of success for MMA athletes to make weight, and help reduce adverse risks of RWL.
Santos-Junior et al., (2020)	Male and female	164/15	19 to 37	Professional	All athletes stated that they had engaged in weight loss procedures to make weight for competition and ~ 35% had engaged in weight loss procedures twice in the last season, usually losing a magnitude of up to 10% of their body mass. About 26% started their weight loss procedures in a time span of 30 days prior to competition. When athletes were analyzed by sex and weight classes, no significant differences were found in the questionnaire score. In contrast, international level athletes showed significant differences in the questionnaire score when compared to state ($P < 0.001$) and national ($P < 0.05$) level athletes. Athletes also reported always use a combination of gradual diet (64.2%), restricting fluid intake (62.6%), and sweat suits (55.9%) as methods to cut weight. Additionally, athletes reported the usage of diuretics (~ 49%) and laxatives (~ 32%) at least once in their professional MMA carrier. The most influential sources of weight management behaviours were coaches, training partners, and physical trainers. Conclusion Brazilian professional MMA athletes commonly undergo weight loss procedures through harmful and illegal methods regardless of sex, weight class, although international level athletes demonstrated weight management behaviour that was found to be even more aggressive.

M: male; F: female. N/A: not available. * Mean considering male and female; RWL: Rapid weight loss

Main findings regarding physiological responses and training characteristics of MMA athletes (n = 13)

Study	Sex	n (M/F)	Age (Years)	Competitive Level	Main Findings
Amtmann., (2004)	NR	28	19-37	Amateur	Overall frequency of strength training sessions/week ranged from 1–7, and overall frequency of fighting specific training sessions/week ranged from 3–12. Five out of the 28 athletes used/had used anabolic-androgenic steroids. Twelve of the MMA athletes did not perform exercises specifically for the neck musculature, and only 8 used the power clean and/or power snatch within their strength-training program.
Amtmann, Amtmann & Spath., (2008)	Male	6	21-41	Amateur	During training lactate ranged from 8.1 to 19.7 mmol, and the RPE was 15 to 19 on the Borg scale (ref). Post bout lactate measurements ranged from 10.2 to 20.7 mmol, and post-bout RPE was ranged 13 to 19.
Coswig et al., (2016)[a]	Male	25	27 ± 5	Professional	No significant differences were observed among the groups for demographic variables. The athletes were 26.5 ± 5 years with 80 ± 10 kg, 1.74 ± 0.05 m and had 39.4 ± 25 months of training experience. Primary results indicated higher blood glucose concentration prior to fights for OFF group (OFF= 6.1 ± 1.2 mmol/L and SIM= 4.4 ± 0.7 mmol/L; $P < 0.01$) and higher ALT values for OFF group at both time points (OFF: PRE = 41.2 ± 12 U/L, POST = 44.2 ± 14.1 U/L; SIM: PRE = 28.1 ± 13.8 U/L, POST = 30.5 ± 12.5 U/L; $P = 0.001$). In addition, the blood lactate showed similar responses for both groups (OFF: PRE= 4 [3.4 - 4.4] mmol/L, POST= 16.9 [13.8 - 23.5] mmol/L; SIM: PRE = 3.8 [2.8

					- 5.5] mmol/L, POST= 16.8 [12.3 - 19.2] mmol/L; P < 0.001). Conclusions: In conclusion, MMA official and simulated matches induce similar high intensity glycolytic demands and minimal changes to biochemical markers of muscle damage immediately following the fights. Glycolytic availability prior to the fights was raised exclusively in response to official matches.
Coswig et al., (2016)[b]	Male	13	25 ± 5	Regional	In biochemical analysis, significant changes (p<0.05) were identified between PRE and POST (glucose: 80.3 ± 12.7 to 156.5 ± 19.1 mg.ml-1; lactate: 4 ± 1.7 to15.6±4.8 mmol/dL), POST and 48h (glucose: 156.5 ± 19.1 to 87.6 ± 15.5 mg/mL-1; lactate:15.6 ±4.8 to 2.9 ± 3.5 mmol/dL; urea: 44.1 ± 8.9 to 36.3 ± 7.8 mg/mL-1), and PRE and 48h (CK: 255.8 ±137.4 to 395.9±188.7 U/L). In addition, time-motion analyses showed a total high: low intensity of1:2 and a effort: pause ratio of 1:3. In conclusion, simulated MMA sparring matches feature 1 moderate to high intensity and a low degree of musculoskeletal damage, which can be seen by6 absence of physical performance and [CK] decreases. Results of the study indicate that sparring training could be introduced into competitive microcycles to improve technical and tactical aspects of MMA matches, due the high motor specificity and low muscle damage.
De Souza et al., (2017)	Male	20	25 ± 2	Professional	It was observed: a significant decrease in T and T/C between moment -24h and 0h and a subsequent increase between the moment 0h and +24h and a reverse behaviour in variables C, LAC and GLU (p<0.0001); a decrease in CK between moment -24h and -1h and an increase between moment -1h and +24h (p<0.0001); and differences between winners and losers T levels, in moments -24h, -1h, 0h and +24h (p = 0.009 e p < 0.001, p = 0.005 e p = 0,001, T and C, respectively), in T/C in the moments -24h and 0h (p=0.006 and p=0.001, respectively)

					and in GLU levels ($p<0.0001$) in the moment 0h. Therefore, it seems that an MMA fight leads to metabolic stress and muscle damage, regardless of the result of the fight. The coaches have now more biochemical and hormonal references and indicators in response to an MMA fight.
Fares et al., (2021)[b]	Male	1070	N/R	Professional	Doping is present in the UFC. Increasing testing numbers, raising awarenes and education on the risks of doping, and conducting further research on the topic is key to help resolve this problem.
Ghoul et al., (2019)	Male	12	26 ± 5	Regional	The experimental protocol consisted of six testing time points Before ($T_{rest)}$, directly after the first (T_{rd1}), second (T_{rd2}), and third (T_{rd3}), rounds, and 30-min ($T_{recovery30min}$) and/or 24-hr ($T_{recovery24h}$) post-competition. Physiological/physical data were assessed before (T_{rest}), directly after round 1 (T_{rd1}), round 2 (T_{rd2}) and round 3 (T_{rd3}), and then 30 minutes ($T_{recovery30min}$) and 24 hours ($T_{recovery24h}$) post competition. Heart rate (HR), rating of perceived exertion (RPE), and blood lactate concentration ([La^-]) were assessed at Trest, Trd1, Trd2, and Trd3. Biological data were collected at T_{rest}, T_{rd3}, $T_{recovery30min}$, and $T_{recovery24h}$. Physical tests were performed at T_{rest}, $T_{recovery30min}$, and $T_{recovery24h}$. HR, RPE, and [La^-] were high during competition. Leukocytes, hemoglobin, total protein, and glycemia were increased at T_{rd3} compared with all other time points (p # 0.05). Cortisol was increased at T_{rd3} compared with T_{rest} and $T_{recovery24h}$ (p # 0.05). Testosterone was higher at T_{rd3} and $T_{recovery30min}$ than T_{rest} (p, 0.001). Higher values of uric acid were noted during recovery periods (p, 0.001). Lactate dehydrogenase was lower at T_{rest} compared with T_{rd3}, $T_{recovery30min}$, and $T_{recovery24h}$ (p # 0.05). Countermovement jump was higher at T_{rest} than $T_{recovery30min}$ ($p = 0.020$). Consequently, MMA is a high-intensity

					intermittent combat sport that induces significant fatigue and muscle damage, both of which are still present at 24 hours post competition.
Gomes-Santos et al., (2022)	Male	12	26.7 6 4.8	Professional	Considering metabolic and mechanical stresses (imposed by combat techniques, e.g., punches and joint locks), this study indicates pre-existing inflammation, although minor oxidative stress, in MMA professionals after combat.
Kirk et al., (2015)	Male	6	26 ± 5	Semi-professional and professional	Mean accumulated fighter load was 224.32 ± 26.59 au, and the load per minute was 14.91 ± 1.78 au. Mean post bout lactate of 9.25 ± 2.96 mmol.L significant differences in lactate levels were found across all six sampling points. The cohort was found to have a work rest ratio (W:R) of 1:1.01. A significant difference was found between bout winners and bout losers in terms of successful takedowns.
Lindsay et al., (2015)	Male	10	27 ± 3	Amateur and professional	Myoglobin was analyzed by reverse-phase high performance liquid chromatography, and urinary neopterin and total neopterin (neopterin+7,8-dihydroneopterin) were measured by strong cation exchange high-performance liquid chromatography. Cold water immersion and passive recovery were compared using changes in these markers, while cryotherapy tested total neopterin production in γ-interferon and phorbol myristate acetate (PMA)- stimulated blood-derived mononuclear cells (monocytes/T cells). Myoglobin significantly increased ($p < 0.05$) at 1 h post-contest, neopterin significantly increased at 1 and 24 h ($p < 0.05$), total neopterin significantly increased ($p < 0.05$) at 1 h post for the passive group only, and significant individual variation was observed for all markers ($p < 0.01$). Cold water immersion attenuated total neopterin production ($p < 0.05$), while cryotherapy significantly reduced total neopterin production in PMA-stimulated mononuclear cells ($p < 0.01$). Cryotherapy attenuates the post-exercise inflammatory response

					following an MMA contest. The evidence also suggests that the mechanisms responsible for this may be related to direct immune cell suppression.
Lindsay et al., (2017)	Male	15	28 ± 6	Semi-professional	Combative sport athletes who are subjected to impact-induced stress may benefit from immediate cold-water immersion as a simple recovery intervention that reduces delayed onset muscle soreness as well as macrophage and HPA activation whilst not impairing functional performance.
Tabben et al., (2018)	Male	12	26.5 ± 5	Regional	The main results indicate that POST-R sprint (5- and 10-m) performances were 'likely to very likely' (d = 0.64 and 0.65) impaired by prior CWI in well-trained MMA athletes. However, moderate improvements were in 10-m sprint performance were 'likely' evident at POST-24 after CWI compared with CON (d = 0.53). Additionally, the use of CWI 'almost certainly' resulted in a large overall improvement in Hooper scores (d = 1.93). Specifically, CWI 'almost certainly' resulted in improved sleep quality (d = 1.36), stress (d = 1.56) and perceived fatigue (d = 1.51), and 'likely' resulted in a moderate decrease in muscle soreness DOMS (d = 0.60).The use of CWI resulted in an enhanced recovery of 10-m sprint performance, as well as improved perceived wellness 24-h following simulated MMA competition.
Wiechmann et al., (2016)	Male	10	28 ± 6	Elite	The number of per- formed upright punches and kicks (UKF) that failed the opponent, the number of obtained hits to the upper and lower body (LBH), as well as the total fight duration (TFD) were evaluated as potential predictors from video recordings. CK peaked 24 h (829±753 U/L^{-1}and Mb peaked 2 h (210±122 μg/L^{-1}post matches. Almost 80% of the peak CK variance could be explained by LBH and UKF, whereas 87% of the Mb variation was explained by TFD and

					LBH. MMA result in a significant skeletal muscle damage, which largely depends on LBH. Furthermore, eccentric contractions to decelerate kicks that missed the opponent and the TFD seem to contribute to the MDM response.

M: male; F: female. N/A: not available. * Mean considering male and female

Main findings regarding technical and tactical analysis in MMA athletes (n = 22)

Study	Sex	n (M/F)	Age (Years)	Competitive Level	Main Findings
Antoniettô et al., (2019)	Male and female	NR	NR	Professional	The analyses were performed according to the duration (Δ) in each phase: Δ standing preparatory activity time, Δ standing combat activity time, Δ ground preparatory activity time, and Δ ground combat activity time and their technical-tactical actions (attempted and landed strikes to the head, body and leg, takedowns, and submissions). The main results demonstrated a shorter Δ standing preparatory activity time in 1R 3 1ER (95.6 ± 62.9 seconds) and 2R 3 2ER (93.6 ± 67.9 seconds) vs. 2R 3 3ER (160.5 ± 87.4 seconds) and 3R 3 3ER (144.0 ± 88.5 seconds) with fewer strikes attempted and landed to the head, body, and legs (p # 0.05). No differences were observed (p . 0.05) between Δ standing combat activity time, but lower attempted and landed takedowns and strikes to the head, body, and leg frequencies. There were shorter Δ ground combat activity time (p # 0.05) in 1R 3 1ER (23.4 ± 45.5) and 2R 3 2ER (25.3 ±41.9) vs. 2R 3 3ER (50.4 ± 69.9) and 3R 3 3ER (52.9 ± 74.2), with lower attempted submissions, chokes, and attempted and landed strikes to the head, body, and leg frequencies observed. These results contribute to the

					information developed from current research to help improve the quality of training and promote effective athletic preparation related to pacing strategy and performance models.
Aung et al., (2021)	Male	475	N/R	Professional	Sexual dimorphism in fo likely arose in the common ancestor of the catarrhine primates after their divergence from the New World monkeys approximately 43.5mya and appears to have been subsequently elaborated or reduced depending on the form and degree of male mating competition. Relatively low male fo may have evolved as a means of exaggerating the appearance of size to same-sex competitors and/or potential mates, but there is considerable debate regarding whether male fo is purely deceptive or provides any reliable information about formidability in men
Brito et al., (2018)	Male and Female	54	NR	Professional	For this, 267 rounds (male and female) were analyzed in professional matches. The rounds were paired by athletes in the conditions: doping, winning and losing. Motor actions were analyzed through a specific and previously-validated protocol. Of the substances detected, anabolic androgenic steroids represented 55% ($p \leq 0.001$). Doped athletes had lower pause time (83.4±68.3 vs. 131.7±95.2, $p \leq 0.001$) and longer time at high-intensity (85.2±86.6 vs. 51.2±73.3, $p = 0.002$) compared to the losing condition. Regarding the technical-tactical analysis in standing combat, winning presented a higher mean compared to doping in all variables except for Knockdowns ($p = 0.08$), single body strikes landed ($p = 0.15$), single leg strikes landed ($p = 0.25$) and single strike attempts ($p = 0.4$). In conclusion, athletes who tested positive presented higher performance in the physical variables (effort and pause time) in comparison to the losing condition; however, doping did not reflect in better technical-tactical performance.

Dal Bello et al., (2019)	NR	NR	NR	Professional	304 rounds of UFC fights. Fights finished by Unanimous decisions showed high frequency of takedowns than another outcome (1.9 ± 1.9 vs. 1.3 ± 1.4 vs. 1.0 ± 1.1 attempts). Bouts with Split Decision demonstrated higher takedowns/round than bouts ended by Submission (0.4 ± 0.7 vs. 0.2 ± 0.6 attempts). TKO/KO showed lower values of sweeps/round (0.0 ± 0.0 vs. 0.1 ± 0.3 attempts) and takedowns attempted/round ($p = 0.014$, 1.3 ± 1.4 vs. 2.0 ± 1.6 attempts) than bouts ending by Split Decision. These results also show that the grappling strategy and tactics are variable depending on the strengths and weaknesses of the athletes, and can be used by coaches and athletes to develop specific training programs.
Del Vecchio et al., (2011)	Male	52	24 ± 5	Professional	Effort-pause ratio (EP) MMA athletes, and classified effort segments of stand-up or groundwork performed per round in MMA matches. Time in groundwork of low-intensity was longer in the second compared to the third round. The effort:pause ratio was 1:2 to 1:4.
Dos Santos et al., (2018)	Male	45	34 to 54	Professional	The main results showed significant differences ($p \leq 0.05$) in total strikes landed [M1: 22(13;34) > M2: 18(10;31.7)]; total strikes attempted [M1: 41(24.5;62) > M2: 35(21;48)], single head strikes attempted [M1: 19(9;34.5) > M2: 16.5(9;28)], single body strikes landed [M1: 1(0;4) > M2: 1(0;2)], single body strikes landed [M1: 2(0;5) > M2: 1(0;3)], takedowns attempted [M1: 1(0;2) > M2: 1(0;2)], standing combat time (M1: 2:10.28±1:38.95 > M2: 1:55.56±1:32.17) and low intensity time (M1: 2:11.45±1:38.95 > M2: 1:56.26±1:31.89). Variables, which increased the probability to be associated with over the years, were body strikes landed, head strikes landed, total strikes landed and single strikes attempted, while body strikes attempted, head strikes attempted, total strikes attempted and submission attempted had a negative association with MMA time experience. Therefore, M2 athletes should

					be focused on stand combat moment combined with strikes landed actions, targeting the head – it has the highest potential performance probability and avoid unsuccessful body strike attempts and submissions – it has the lowest potential performance probability over 10 years.
Fernandes et al., (2018)	Male	NR	NR	Professional	The analyses showed higher frequency after rule changes of total strike attempts (41.5±25.9 and 43.6±26.4 for Before vs. After; p=0.03), single strike attempts (31.0±22.8 and 34.8±24.8 for Before vs. After; p≤0.001), single head strike attempts (24.1±19.6 and 27.4±21.5 for Before vs. After; p≤0.001), single body strike attempts (4.0±4.2 and 4.4±4.3 for Before vs. After; p≤0.001), single strike landed (13.3±11.1 and 14.7±11.0 for Before vs. After; p≤0.001), single head strike landed (8.1±8.5 and 9.2±8.3 for Before vs. After; p≤0.001), and single leg strike landed (2.3±3.15 and 2.5±3.2 for Before vs. After; p=0.02). There was a higher exposure time after the changes (383.3 vs. 480.2; for Before vs. After, p=0.019). Furthermore, there was a higher frequency of bouts ended in the last round (5º) after the rule changes (90 vs. 150; p=0.006). Our results showed higher exposure time and frequency of technical-tactical actions correlated with injury incidence in professional MMA..
Follmer, Andreato & Coswig., (2021)	Male and Female	1728/175	N/R	Professional	This study aimed to assess the ratios of match-ending due to submission, as well as the frequency of submission techniques to finish a combat among the weight and gender categories in modern MMA. The main finding is that, in general, lighter athletes presented higher chances to end matches by submission. Rear and front chokes accounted for the majority of submission tech- niques, whereas the elbow was the most attacked body site for joint locks.
James et al., (2017)[a]	Male	NR	NR	Professional	Effect size revealed differences between gains and losses in various performance indicators. The decision tree (gross: 71.8%; on the rate

					scale: 76.3%) and DFA discriminant function analysis analyzes (gross: 71.4%; on the rate scale 71.2%) achieved similar classification accuracies. The fight and precision performance indicators were the most influential in explaining the results. Decision tree analysis suggests that fighting activity and technique accuracy are of particular importance to achieving victory in elite MMA competitions. The DFA results supported the importance of these performance indicators. Decision tree induction represents an intuitive and slightly more accurate approach to explaining the fighting outcome in this sport compared to DFA.
Miarka et al., (2015)	Male	NR	NR	Professional	In the 1st round, heavyweights (212.4±101.5s) presented with a shorter effort time than all other weight divisions (257.6±79.9s).In the 2nd round, the bantamweight competitors (132.8±90.9s) presented with the shortest total effort time (171.7±81.5s).In the 3rd round, the shortest total effort time was in the heavyweight division (246.3±89.1s) again, with the other weight divisions showing variable effort times, including: welterweight (289.6±42.3s), lightweight (280.3±57.3s), featherweight (281.4±58s), bantamweight (285.6±47.2s), flyweight (287.7±43.8s) and middleweight (268.3±72.2s).
Miarka et al., (2016)[a]	NR	NR	NR	Professional	824 rounds performed over 351 bouts in UFC events were studied. Winners had higher values for total strikes and submissions in all rounds, and also positional improvements over losers.
Miarka et al., (2016)[b]	Female	NR	NR	Professional	The principal findings showed significant differences between Split and Unanimous Decision outcomes vs. KO/TKO and Submission groups in stand-up combat with low intensity by round (160.4±83.6s and 158.4±87.6s vs. 44.8±38.8s and 42.1±44.1s, respectively, $p<0.001$) and in total combat time by round (300.7±0.3s and 300.0±0.4s vs. 154.4±95.2s and 204.2±96.6s, respectively, $p<0.001$). Significant

					differences were found when compared the Total, Head and Leg Strikes Attempts, where winners by KO/TKO and Submission demonstrated lower frequencies than Split and Unanimous Decision($p<0.001$; for all comparisons).. In conclusion, technical-tactical skills can be associated with contextualized practices, where female athletes who finalized the matches by KO/TKO and Submission had higher values of striking and grappling actions during the groundwork combat, while those who had Split or Unanimous Decision outcomes showed higher values of striking actions during stand-up combat.
Miarka et al., (2016)[c]	Male	NR	NR	Professional	202 UFC bouts three round bouts were analysed for this study. A significant effect was observed when comparing home vs. away bouts in total strikes landed, total strikes attempts, head strikes attempts, and in body strikes attempts. Time-motion and technical-tactical differences did not impact the combats' outcome.
Miarka et al., (2017)[a]	NR	NR	NR	Professional	For this, we analyzed 2814 rounds of all weight divisions by motor actions and spatiotemporal changes according actions and time of the Keeping distance, Clinch and Groundwork combat phases. We observed differences between weight divisions in the keeping distance on stand-up combat ($p \leq 0.001$; with lower time in Featherweight 131.4 s and bantamweight 127.9 s) clinch without attack ($p \leq 0.001$; with higher timer in Flyweight 11.4 s and Half-middleweight 12.6 s) and groundwork without attack ($p \leq 0.001$; with higher timer in Half- middleweight 0.9 s). During keeping distance, half-middleweight presented a higher frequency of Head Strikes Landed ($p = 0.026$; 7 ± 8 times) and attempted ($p = 0.003$; 24 ± 22 times). In clinch actions heavyweight present a higher frequency ($p \leq 0.023$) of head strike landed (3 ± 7 times) and attempted (4 ± 9 times) and

					half-middleweight for body strikes (p ≤ 0.023) landed (2 ± 5 times) and attempted (3 ± 5 times). At the last, during groundwork, Bantamweight present a higher frequency (p ≤ 0.036) of head strikes landed (8 ± 10 times) and attempted (10 ± 13 times) body strikes landed (p ≤ 0.044; 3 ± 5 times) and attempted (3 ± 6 times). This study reveals important point to training and provide a challenge applied referential to the conditioning plains. From the weight divisions differences should be aware of the increase in the frequency of distance actions, especially in light and middleweights. On the Ground, bantam- weight can focus on striking and grappling actions than others.
Miarka et al., (2017)[b]	NR	NR	NR	Professional	The sample was composed of 1,752 rounds of 584 UFC bouts (Winning vs. Losing) from 67 events in 2013 and 2014. Technical–tactical actions during spatiotemporal changes (i.e. keeping distance, clinch and ground) were observed, according to frequency of attempted and landed strikes (separated in leg, body and head orientations), takedowns, chokes, locks and submissions. Wilcoxon was applied to compare outcomes, and a logistic regression analysis was used to confirm the effects of technical–tactical actions on the dichotomous performance (losing vs. winning), p ≤ .05. The main results showed significant differences in all spatiotemporal changes, except for head and leg strike attempts while keeping the distance. The performance probability was 69.6% for outcomes, 64.5% for winning vs. 74.7% for losing. Furthermore, when verified the Wald's criterion, it is possible to corroborate that Head Strikes Landed Keeping Distance, Offensive Passes and Takedowns Landed were the most significant variables, which contributed to probability of predicting winning bouts.

Miarka et al., (2019)[b]	Male	2320	N/A	Professional	The main cause of injuries in doctor stoppage situations were due to facial injuries (>90%), with 87% occurring after striking actions during the second round. Lacerations were the leading type of injury, which occurred with 80% frequency.
Miarka et al., (2018)[a]	Male	364	20 to 48	Professional	Analyzed 1,564 rounds of 678 UFC bouts. Our results indicated that KO/TKO is the main outcome that defines the ending round (≈60%), however, there is a higher frequency of ending by submission on the 1st and 2nd round (>30%). Bouts ending during the 1st or 2ndrounds had shorter total time and standing combat with low-intensity than ending in the 3rd round (91.5±71.4, 93.4±67.5 and 143.2±87.4; for low-intensity in the 1st, 2nd and 3rd round respectively; p<0.05), while standing combat time with high intensity was longer in the last round in comparison to bouts that finished in the 1st<br>or 2ndrounds (7.4±9.2, 9.7±18.0 and17.7±29.1 for high intensity in the 1st, 2nd and 3rdround respectively; p<0.05). The lower time dedicated to low-intensity stand up combat actions, regardless of round, and forcefulness of the actions in groundwork in the 1st and 2nd rounds appear to be elements that increase the probability of success in professional MMA bouts, these factors have essential implications related to training program design.
Miarka et al., (2019)[a]	Male	NR	NR	Professional	3573 bouts of professional UFC events were analysed. Analysed 779 rounds (264 bouts – 3rd round n = 120; 4th round n = 92; and; 5th round n = 52) which were compared by rounds. Our main results showed that bouts with 5th showed higher percentage/ round of low-intensity time than the 3rd round (79% vs. 75%) and the 4th rounds demonstrated higher percentage/round of low- intensity time effort 20 than other rounds (84% vs. 76%). Regarding striking actions, 1st round (39.5 ± 17.6) and 4th round (19.8 ± 12.5) showed lower

					frequency of strikes attempted than 2nd round (46.1 ± 21.9) and 3rd round (46.1 ± 27.0). This study demonstrated new parameters for long MMA bouts with 3, 4 and 5 ending- rounds and other rounds
Miarka et al., (2021)	Female	74	NR	Professional	The present research sought to understand the mechanisms involved in pacing strategy and technical-tactical behaviors used in the reactive/proactive dynamics by a round of female professional MMA combat. The main results indicated an increase in the frequency of attacks to the head, body, leg orientations, and the preparatory activity time compared to 1ER vs. 3ER. The correlation of round phases during MMA combats and critical technical-tactical behaviors, such as takedowns, submissions, and strike attempts in different orientations (i.e., to the head, body, or leg), represent the main aspects of attacking systems used by women. Besides, to obtain information for conditioning and strength training of female athletes, it is significant to highlight that open task and intermittent combat sports involve complex sequential skills, with a range of around 92.2 ± 71.0–162.3 ± 89.4s of low-intensity behaviors to 33.5 ± 52.2–69.8 ± 79.6s by high intensity.
Richardson ., (2021)	Male and female	3357	N/R	Professional	I show that arm span to height ratio shows positive allometry with weight and its use does not fully remove the influence of height. Once height and weight are controlled for, I show that male MMA fighters have longer arms than fe- male fighters, and that fighters with longer arms of both sexes win a greater percentage of their fights. These results are consistent with the hy- pothesis that arm length is a male intrasexually selected adaptation for combat.

Stellpflug, Menton & LeFevere., (2022)	N/R	N/R	N/R	Professional	This was a descriptive exploration into the fight-ending chokes across the history of the UFCTM. The data presented can help guide Sports Medicine physicians to provide a safe environment at events. Knowledge of the frequency of certain fight events can guide training on what they should be prepared for and what to look for during the action and afterward. Ringside physicians should have an understanding of the types of chokes that result in submissions, as well as how often these chokes result in a loss of consciousness. This will help the physician recognize when a fighter is in danger, and assist in keeping the athletes healthy and safe. Additionally, competitors can use the information to help guide the most likely attacks to prepare for in the course of a fight or grappling match in order to recognize when they are in danger.
Schild & Zettler., (2021)	Male	135	N/R	Amateurs	The results indicated no significant relation between a fighter's voice pitch, as directly measured before a fight, and successive fighting success in both mixed martial arts fighters and boxers.

M: male; F: female. NR: not reported. * Mean considering male and female.

Main findings regarding physical fitness in MMA athletes (n = 6)

Study	Sex	n (M/F)	Age (Years)	Competitive Level	Main Findings
Giboin & Gruber., (2019)	Male	9	28 ± 7	Amateur and professional	Estimated neuromuscular fatigue of knee extensors muscles during and after an MMA training protocol designed to simulate the physiological demands of MMA competition in competitive practitioners (n = 9) with isometric maximal voluntary force (MVF), potentiated muscle twitch at rest (Ptw), and voluntary activation (VA). Bayesian linear mixed models showed that the training protocol induced a reduction of MVF, Ptw, and VA. Although the largest reduction across time of VA was smaller than the largest reduction of Ptw, an effect of VA, but not of Ptw, was found on MVF variation. The training protocol induced neuromuscular fatigue, with a larger peripheral (Ptw) than central component (VA). However, despite the large decrease in Ptw, force production capacity was related only to VA, indicating that central control might play an important role in the compensation of the peripheral fatigue components estimated with Ptw. This central compensation can most probably prevent a too large loss of muscle force during the training protocol.
James et al., (2017)[b]	Male	29	28.1 ± 5.1	Semi-Professional	The HL competitors produced significantly superior values across a multitude of measures. These included 1RM squat strength (1.84 ± 0.23 vs 1.56 ± 0.24 kg.BM-1; P=0.003), in addition to performance in the incremental load jump squat that revealed greater peak power (P=0.005-0.002), force (P=0.002-0.004) and velocity (P=0.002- 0.03) at each load. Higher measures of impulse (P=0.01-0.04) were noted in a number of conditions. Average power (P=0.002-0.02) and velocity (P=0.01-0.04) at all loads in addition to a series of rate-dependent

					measures were also superior in the HL group (P=0.005-0.02). The HL competitors' 1RM bench press values approached significantly greater levels (P=0.056), while IMTP performance did not differ between groups. Maximal lower body neuromuscular capabilities are key attributes distinguishing HL from LL MMA competitors. This information can be used to inform evidenced-based training and performance monitoring practices.
James et al., (2018)	Male	15	N/A	Amateur and professional	Found practically relevant (nontrivial) differences between semi-professional and amateur MMA competitors across sprints, repeated sprint ability RSA and Yo-Yo Intermittent Recovery Level 2 Test (IR2). These results provide an indication of the relevance of short-term anaerobic qualities, the ability to repeatedly produce maximal expressions, and intermittent aerobic capacity to MMA performance.
James et al., (2020)	Male	27	19-34	Amateur and professional	Power, force, velocity, displacement-time waveforms and eccentric phase displacement, eccentric time, eccentric impulse, and the modified reactive strength index (RSImod) were compared between groups using statistical parametric mapping procedures and independent t-tests. Power (between 65 and 71% of the power-time curve) was greater in the HL than that of the LL group ($p = 0.01$) despite no differences in eccentric displacement ($p = 0.50$) or movement time ($p = 0.17$) between groups. The HL group demonstrated a greater RSImod ($p = 0.05$) alongside a reduced eccentric time ($p = 0.02$) and eccentric impulse ($p = 0.02$). Timing and control of lower-body force production contributed to between-group differences in CMJ performance among MMA competitors.
Marinho et al., (2016)	Male	8	31 ± 5	Professional	Body fat levels of 13.4 ± 5.6 %, lean mass levels of 69.6 ± 4.6 %, and mesomorphic component (6.4 ± 0.8) were observed. Athletes

					performed 42 ± 14 sit-ups and 37 ± 9 push-ups, and remained for 35 ± 10 s in the flexed-arm hang test. Athletes reached 2.19 ± 0.31 m in the horizontal jump test and obtained an absolute 1-RM values of 80 ± 15 kg and 68.5 ± 6.0 kg and relative values of 1.00 ± 0.2 kg/kg and 0.84 ± 0.10 kg/kg in bench press and squat tests, respectively. Body fat levels were in accordance with other studies finding high lean body mass, and a predominantly mesomorphic component. Abdominal and upper limb endurance were classified as excellent, lower limb performance in the horizontal jump was classified as weak.
Plush et al., (2021)	Male	6	N/R	Amateur and professional	One of the main observations from this study was that the testing battery was viable and able to be completed by the subjects recruited in a timely fashion, as an athlete could get through all testing within 7 days when accounting for familiarization to testing protocols. Additionally, the VAS scale used appeared to show differences in competitive styles between the participants of the study.

M: male; F: female. N/A: not available. * Mean considering male and female

Main findings regarding psychobiological parameters in MMA athletes (n = 11)

Study	Sex	n (M/F)	Age (Years)	Competitive Level	Main Findings
Chen & Cheesman., (2013)	Male	158	27 ± 5	Amateur and professional	The results showed that certain subscales of the inventories did indeed discriminate between the competitor's status, with statistically significant results being obtained on Determination, Positive cognition (PPI–A), and Confidence (SMTQ), thus

					supporting the hypothesis. The PPI–A showed professionals to have on average 10% higher Mental Toughness scores than semi-professional competitors and 9% higher than amateur athletes. For the SMTQ, these values were 7% and 9%, respectively.
Cherepkova et al., (2019)	Male	107	33 ± 6	N/A	Among those convicted of felonies and serious crimes, carriers of DRD4 long alleles are found more frequently, similarly to the cohort of MMA fighters (lacking criminal record in both pathernal lines). A frequency of the combination of the DRD4 genotype 4/7 and DAT genotype 10/10 is clearly higher among the convicts of violent crimes and MMA fighters. One can speculate that the presence of a "controlled aggression" without a predisposition to pathological violence is found in MMA fighters. The hypothesis of genetic predisposition to different variants of extreme behaviour mediated by genetic determinants involved in the functioning of neuromediator systems including those controlling dopamine pathways.
Dixson et al., (2018)	Male	395	N/A	Professional	Found no evidence that beardedness was associated with fewer losses by knock-out or greater fighter ability. While fighters with longer reaches won more fights, neither stance nor past experience influenced fight outcomes. The study suggests that beards represent dishonest signals of formidability that may serve to curtail the escalation of intra-sexual conflict through intimidation rather than providing advantages in direct combat.
Little et al., (2015)	Male and female	196	29 ± 11	N/A	Individuals performed at rates above chance in correctly selecting a fight winner as more likely to win the fight than the loser. Winners were seen to be more masculine, stronger, and more aggressive than losers. Women saw the winners as more attractive than the losers.

Massey et al., (2013)	Male and female	8/1	N/A	Amateur and professional	Throughout data collection and analysis, self-regulation during a training camp emerged as integral to optimal performance. Motivation and ongoing evaluation aided fighters in their ability to self- regulate both external and internal factors related to training and performance. External factors associated with self-regulation consisted of the creation and maintenance of an ascetic routine through environmental regulation, social support, and structured amnesty. Internal factors associated with self- regulation consisted of deliberately induced pain and distress, which facilitated self-efficacy, and produced stress and fatigue. Results of the current study advance the scientific literature in sport psychology by examining the role of self-regulation in the performance of MMA fighters. As the popularity of MMA continues to increase, future research should also examine the role of self-regulation in other aspects of MMA (e.g., injury rehabilitation, life balance).
Niewczas et al., (2021)	Male	85	25.95 ± 6.69	Professional	We observed a statistically significant effect of a complex factor of the *DRD2 rs1799732* genotype on MMA participants' control and reward dependence. Engaging in high-risk sport may be associated with several personality characteristics. The *DRD2 rs1799732* polymorphism may be associated with reduced harm avoidance in martial arts athletes, thereby modulating athletes' predisposition to participate in high-risk sport.
Pavelka et al., (2020)	Male	45	27 ± 6	Professional	Greater amounts of fatigue during the Wingate test seemed to negatively affect the consistency of post-Wingate movement time. Due to cumulative fatigue and the dynamic nature of MMA, this indicates that not only decrements in aerobic and anaerobic power likely affect a fighter's performance, but their reaction time and motor function may also be compromised during a fight.
Peacock et al., (2018)[a]	Male	8	28 ± 3	Professional	Significant correlation between sleep latency and VO2max, heart rate recovery, prowler sled push, vertical jump, and missed practice sessions. Significant correlation Average fall asleep time and heart

					rate recovery. Sleep efficiency, heart rate recovery, and missed practice sessions. Significant correlation MMA athletes who exhibited consistency in sleep demonstrated stronger relationships with performance testing during the fight-camp period.
Petrou et al., (2021)	Male	249	Median = 26–30	Professional and Amateurs	The study examined, for the first time, the multivariate association between social norms, negative self-conscious emotions (i.e., anticipated guilt, shame, and embarrass- ment), and self-regulatory efficacy and doping intentions in an international sample of MMA athletes, with an emphasis on moderation and mediation effects. We also examined whether MMA athletes with different doping experiences reported stronger pro-doping intentions and social norms, and lower scores in self-conscious emotions and self-regulat- ory efficacy.
Ruiz-Barquín et al., (2019)	Male and female	34/8	M: 26 ± 4<br>F: 26 ± 4	Amateur and professional	MMA athletes obtained higher scores than those reported by samples of other sports in previous studies, and more specifically in factors I - Support for long-term success, II - Imagery use during practice and competition, and IV - Ability to organise and engage in quality practice. This can be explained due to the higher average age of our sample and the professional or semi- professional level already achieved by many of the MMA fighters. The study also revealed that MMA fighters may need specific psychological training related to factor III - Coping with performance and developmental pressures.
Sinnett, Maglinti & Kingstone., (2018)	Male and female	18/2	20-35	Amateur	Grunting was advantageous in terms of not only generating increased force when kicking, but also as a means of distracting an opponent leading to slower and more error prone responses.

M: male; F: female. N/A: not available. * Mean considering male and female

Main findings regarding interventions applied in mixed martial arts (MMA) athletes (n = 10)

Study	Sex	n (M/F)	Age (Years)	Competitive Level	Main Findings
Azevedo et al. (2019)	Male	11	28 ± 4	Professional	The study used a double-blind, counterbalanced, crossover design. Using a force transducer attached to a cushioned plate, the punch frequency, and mean and maximal punch force was measured. The readiness to invest in both rate how physically (RTIPE) and mental (RTIME) effort was assessed prior to the protocol, and the rating of perceived exertion (RPE) was recorded after. Caffeine ingestion did not result in increased punching frequency, mean and maximum punch force, RTIPE, RTIME, and RPE when compared to the placebo condition. acute caffeine ingestion did not improve punching performance in professional MMA athletes.
Bodden et al., (2015)	Male	25	24 ± 5	Amateur	There was a significant difference in FMS test scores between control and intervention groups at both 4- and 8-week periods. Additionally, the results of this study suggested that a 4-week intervention program of corrective exercise was successful at significantly improving FMS scores. It was noticed that a number of subjects at week 4 testing achieved considerable improvements in FMS scores, which in theory would have changed the subjects' weakest movement patterns and therefore their corrective exercise program. However, to standardize the study, subjects persisted with the program from the initial screening for the entire 8-week period. A built-in exercise progression would have been valuable at week 4 to target the change in the weakest links of the improved subjects, which consequently could have produced further improvements during this period. Functional movement screen (FMS) based programmers improved FMS scores.

Chernozub et al., (2018)	Male	30	21 ± 1	More than 2 years	One of the priority factors, together with high technical and tactical training of athletes, allowing to achieve a short-term and bright victory in combat in mixed martial arts is the level of development of the body power capabilities. It was established that the optimal power loads for fighters using the strike style of fighting was to use a high-intensity regime when working with an alactate or lactate energy supply system. In turn, the most effective power loads, for the maximum realisation of functional potential in athletes prioritising the wrestling style during the fight, was the use of low-intensity regimes with a large amount of work in the glycolytic power supply system. The analysis of the results obtained during the experiment demonstrates the need for using models of training sessions developed in the process of power training of MMA fighters, taking into account the particular fighting style.
Chernozub et al., (2019)	Male	40	21 ± 1	Amateur	The obtained results show that athletes of group A increased their circumference indices by 1.9% on average after 3 months of research. In group B, for the same period of time, the studied indices increased by 5.5% on average compared to the baseline data. It was noted that at the beginning of the study, the cortisol concentration in blood serum of group A athletes decreased after exercises by 23.9% in comparison with the basal ones. This fact indicates compensatory reactions to the stimulus and high energy costs. We observed completely opposite changes in group B sportsmen. They were expressed in the increase of the studied indicator by 46% in comparison with the data recorded before exercises. This fact testifies to the adequacy of the loads to the functional body capabilities of this group of athletes. The results of biochemical control of the changes in the hormone testosterone and the enzyme

					lactate dehydrogenase in the blood of both groups' athletes confirm the fact that it is the use of high-intensity loads that promotes the enhancement of the athletes' adaptive body capabilities for training activities in MMA. Conclusions: The analysis of the results obtained during the series of experimental studies indicates the necessity of correcting load parameters during power training. It should be based on the analysis of athletes' adaptive and compensatory body reactions to a stress stimulus. Otherwise there should be developed completely new training programs.
Ignatjeva et al., (2021)	Male	31	N/R	Professional	Performing the progression of the external load may allow the detection of the dominance of one lower limb and determination of the dynamics of differences in the reaction time of the frontal and back limb in MMA fighters.
Kostikiadis et al., (2018)	Male	17	27 ± 5	Professional	Before and after the four-week training period (3 days per week), body composition, aerobic fitness, strength, power and speed were evaluated. Significant improvements in estimated VO2max, average power during the 2000 m rowing, bench press, back squat and deadlift 1RM, SJ power, CMJ height power, medicine ball throw velocity, 10 m sprint and 2 m take down speed and fat-free mass were found only in the STG (3.7 to 22.2%; $p < 0.05$; Hedge's g = -0.42 – 4.1). No significant changes were found for the RTG ($p = 0.225$ to 0.811). Significant differences between the groups were found for almost all post-training assessments ($p < 0.05$; Hedge's g = 0.25 – 1.45) as well as for the percentage changes from pre to post training ($p < 0.05$; Hedge's g: 0.25 – 1.45). Significant relationships were found between percentage changes in fat-free mass, endurance capacity, muscle strength/power and speed (r: - 0.475 to 0.758; $p < 0.05$). These results suggest that a high-intensity low-volume

					strength and conditioning training intervention designed according to the demands of MMA competition may result in significant performance improvements for well-trained fighters.
Kirk et al., (2021)	Male and female	9/5	22.4 ± 4.4	Amateurs and Professional	In contrast to our hypothesis, we observed limited evidence of training periodisation within or between weekly microcycles. Additionally, differences in training load practices between athletes preparing for competition and those in normal train- ing was limited to the final week before competition, largely reflective of reduced training duration. Related to this limited evidence of periodisation, we observed no changes in ratings of fatigue or soreness throughout the 8-week observational period.
[I]Peacock et al., (2018)[b]	Male	12	25,2 ± 2,3	Professional	With the recent rise in popularity of Mixed Martial Arts (MMA), it is imperative to evaluate the effectiveness of strength and conditioning (S&C) modalities while encouraging other professionals. Results show that after 6 weeks, Tai Chi (TC) training in conjunction with the traditional strength and conditioning (S&C) modalities training schedule improves flexibility and balance. These results may also be beneficial for athletes interested in improving various aspects of performance during MMA training.
Total et al., (2019)	Male	15	N/A	Professional	After a conditioning training program, body fat mass decreased while anaerobic peak power and aerobic performance improved. Improvement in the time to obtain and maintain peak power in the upper limbs was noted. Training periodization resulted in advantageous body composition changes and improved physical fitness of the MMA athletes.
Zebrowska et al., (2019)	N/A	80	28 ± 6	N/A	The muscle fatigue test reduced Fmax in all subjects, but in the groups receiving manual lymphatic drainage MLD, deep oscillation DO, and physical stimulation BF significantly higher Fmax was

					observed at recovery compared with post-ex values. The application of MDL reduced the postexercise blood LA and postexercise muscle tension. the effect of lymphatic drainage (PMLD) on postexercise muscle regeneration in MMA athletes was determined by analysing variables characterizing the ability of muscles to work and generate maximum power. One of its main findings is that in the first phase of postexercise recovery (Rec20min), the selected PMLD increased maximal strength of the forearm muscles while reducing their tension. This indicates that they can be used to enable an athlete to perform repetitive efforts.

M: male; F: female. N/A: not available. * Mean considering male and female
