# Supplementary material for: Exploratory Systematic Review of Mixed Martial Arts: An Overview of Performance of Importance Factors with over 20,000 Athletes
Source: Sports (Basel). 2022 May 24;10(6):80. doi: 10.3390/sports10060080 (PMC9227211; doi:10.3390/sports10060080)
Supplement: Supplementary file 1 [file sports-10-00080-s001.zip › Table S4 Summary of studies reporting Effect Size.pdf]

Table S4**: Summary of the 112 studies included in the** exploratory **systematic review**

**Autors**	The effect size has been calculated.	Summary
Alves et al., (2018)	Yes	The aimwas examine the effects of rapid weight loss (RWL) on body mass (BM), strength, and hydration status, and its effects on fight performance in mixed martial artists (MMA). Athletes had BM, handgrip strength, and hydration status assessed at baseline (the official match weigh-in) and at match time 24 hrs later. There were statistically significant changes and very large and large effect sizes (ES) demonstrating decreased BM and handgrip strength, and increased urine density from baseline to match time, indicating that athletes could not fully restore physiological function within the 24 hrs between the official weigh-in and match time. However, at match time only 2 subjects were well-hydrated, while 5 subjects had minimal dehydration, and 5 subjects were significantly dehydrated. Therefore, the findings indicate that the negative effects of RWL on physiological function are not fully regained in the 24-hr period between the official weighin and the start of a match in MMA athletes.
Amtmann (2004)	No	The survey attempted to gather information regarding overall training volume, supplement use, and specific exercises used. The survey return rate was 100% (28/28). 25 out of the 28 athletes supplemented their training with strength training. Overall frequency of strength training sessions/week ranged from 1–7, and overall frequency of fighting specific training sessions/week ranged from 3–12. Five out of the 28 athletes used/had used anabolic-androgenic steroids. 12 of the MMA athletes did not perform exercises specifically for the neck musculature, and only 8 used the power clean and/or power snatch within their strength-training program. The results suggest that strength and conditioning specialists should educate MMA athletes regarding the importance of balanced training, effective exercises, and the side effects of anabolic androgenic steroid use.

Amtmannet al., (2008)	No	Lactate and rate of perceived exertion (RPE) was monitored in 6male subjects training for and competing in a mixed martial artsevent held in Butte, Montana, to determine 1) the metabolicdemands of the sport and 2) the effectiveness of the preboutinterval training programs chosen to help prepare the competitorsfor this event. The training lactate measurementsranged from 8.1 to 19.7 mmol_L21, and the training RPE levelsranged from 15 to 19 on Borg's Category Scale of perceivedexertion, the scores of which ranged from 6 to 20. The postboutlactate measurements ranged from 10.2 to 20.7 mmol_L21, andthe post-bout RPE measurements ranged from 13 to 19. Of the4 subjects that had both training and postbout lactate measurements,3 had obtained lactate levels during training thatexceeded lactate levels immediately after the bout. Thisindicated that, when using lactate measurements as a benchmark,the conditioning training was effective for these 3 athletes.When we used RPE scores as a benchmark, theconditioning was effective for all 4 subjects because allsubjects reached 18–19 during their training, which was atleast as high as their reported post-bout RPE levels.
Andreato et al., (2014)	Yes	Introduction. The aim of this study was to analyze the magnitude and methods of weight loss among MMA fighters and theinfluence of this practice in hydration and profile of mood state.Material and methods. The sample was composed by eight MMA fighters during a professional MMA competition, but onlyfive athletes finished all procedures. To evaluate the weight loss prevalence, magnitude and methods it was applied the RapidWeight Loss Questionnaire adapted to MMA. Saliva samples were taken in the official weigh-in and one hour before of the combatsto estimate the salivary osmolality. To evaluate the profile of mood states the Brunel Mood Scale (BRUMS) was applied.Results: The results showed that all participants already had lost weight to take part in another event. The athletes reportedthat they always adopted as method to lose weight: to make more exercise (50 %) and reduce food intake of liquids (37.5 %).The dangers methods to health were cited by

		athletes in the following sequence: use of diuretics (37.5%), sauna (37.5%), trainingintentionally in heated training rooms (50 %) and use of diet pills (12.5%). In the competition analyzed 88% of athletes reducedtheir body mass (4.5 Å} 4.2 kg, 5.4 Å} 4.3 %) to compete. The salivary osmolality did not show significant difference between weighin(55.6 Å} 30.7 mOsmol/kg H2O) and pre-match moment (40.2 Å} 27.9 mOsmol/kg H2O). The profile of mood states did not changebetween the weigh-in (tension: 1.2 Å} 1.8, depression: 0 Å} 0, anger: 1.2 Å} 1.6, vigour: 14.0 Å} 0.7, fatigue: 2.6 Å} 5.3, confusion:0.2 Å} 0.4) and the pre-match moment (tension: 2.6 Å} 2.2, depression: 0 Å} 0, anger: 1.4 Å} 1.9, vigour: 15.6 Å} 0.5, fatigue: 0.0,confusion: 2.8 Å} 3.9).Conclusions. The conclusion is that there is a great prevalence of weight loss in MMA athletes in this sample. Furthermore,dangers methods to health are or were practiced to reduce the body mass to compete.
Antoniettô et al., (2019)	No	Suggestionsfor professional mixed martial arts training with pacingstrategy and technical-tactical actions by rounds. J StrengthCond Res XX(X): 000–000, 2019—This study compared thepacing strategy and motor actions used in mixed martial artscombats ending by knockout/technical knockout (KO/TKO) orsubmission. All of the sample bouts ended in KO/TKO andconsisted of 1,564 rounds of 678 bouts. The bouts were separatedby round (R) of bouts ending (ER) in the first round (n =192), first (1R 3 2ER) and second (2R 3 2ER) of boutsending in the second round (n = 172), and first (1R 3 3ER),second (2R 3 3ER), and third (3R 3 3ER) of bouts ending inthe third round (n = 1,200). The analyses were performedaccording to the duration (Δ) in each phase: Δ standing preparatoryactivity time, Δ standing combat activity time, Δ groundpreparatory activity time, and Δ ground combat activity timeand their technical-tactical actions (attempted and landedstrikes to the head, body and leg, takedowns, and submissions).The main results demonstrated a shorter Δ standingpreparatory activity time in 1R 3 1ER (95.6 6 62.9 seconds) and 2R 3 2ER (93.6 ± 67.9 seconds) vs. 2R 3 3ER (160.5

		± 87.4 seconds) and 3R 3 3ER (144.0 ± 88.5 seconds) withfewer strikes attempted and landed to the head, body, and legs(p # 0.05). No differences were observed (p . 0.05) between Δ standing combat activity time, but lower attempted andlanded takedowns and strikes to the head, body, and leg frequencies.There were shorter Δ ground combat activity time (p # 0.05) in 1R 3 1ER (23.4 ± 45.5) and 2R 3 2ER (25.3 ± 41.9) vs. 2R 3 3ER (50.4 ± 69.9) and 3R 3 3ER (52.9 ± 74.2), with lower attempted submissions, chokes, and attemptedand landed strikes to the head, body, and leg frequenciesobserved. These results contribute to the information developedfrom current research to help improve the quality of trainingand promote effective athletic preparation related to pacing strategy and performance models.
De Azevedo et al., (2019)	No	Abstract: Mixed martial arts (MMA) is a combat sport where competitors utilize strikes (punches,kicks, knees, and elbows) and submission techniques to defeat opponents in a cage or ring. The aimof this study was to investigate the effect of acute caffeine ingestion on punching performance byprofessional MMA athletes. The study used a double-blind, counterbalanced, crossover design.Eleven professional MMA competitors (27.6 ±4.3 years and 83.5 ±7.8 kg of body weight) ingested a dose of caffeine (5 mg_kg/1) or placebo 60 min prior to three sets of punching. Each set consistedof 15 s, at which participants were asked to perform straight punches with maximum strength andfrequency with his dominant arm. After each set, a 45 s recovery time was applied. Using a forcetransducer attached to a cushioned plate, the punch frequency, and mean and maximal punch forcewas measured. The readiness to invest in both physical (RTIPE) and mental (RTIME) effort wasassessed prior to the protocol, and the rating of perceived exertion (RPE) was recorded after. Caffeineingestion did not result in increased punching frequency, mean and maximum punch force, RTIPE,RTIME, and RPE when compared to the placebo condition. Based on these results, acute caffeineingestion did not improve punching performance in professional MMA athletes.

Aung et al., 2021	Yes	Human voice pitch is highly sexually dimorphic and eminently quantifiable, making it an ideal phenotype for studying the influence of sexual selection. In both traditional and industrial populations, lower pitch in men predicts mating success, reproductive success, and social status and shapes social perceptions, especially those related to physical formidability. Due to practical and ethical constraints however, scant evidence tests the central question of whether male voice pitch and other acoustic measures indicate actual fighting ability in humans. To address this, we examined pitch, pitch variability, and formant position of 475 mixed martial arts (MMA) fighters from an elite fighting league, with each fighter's acoustic measures assessed from multiple voice recordings extracted from audio or video interviews available online (YouTube, Google Video, podcasts), totaling 1312 voice recording samples. In four regression models each predicting a separate measure of fighting ability (win percentages, number of fights, Elo ratings, and retirement status), no acoustic measure significantly predicted fighting ability above and beyond covariates. However, after fight statistics, fight history, height, weight, and age were used to extract underlying dimensions of fighting ability via factor analysis, pitch and formant position negatively predicted "Fighting Experience" and "Size" factor scores in a multivariate regression model, explaining 3–8% of the variance. Our findings suggest that lower male pitch and formants may be valid cues of some components of fighting ability in men.

Barley et al., (2018)[a]	No	Purpose: Combat sports are typically divided into weight classes and body mass manipulation to reach a weight class is commonplace. Previous research suggests that mixed martial arts (MMA) weight loss practices may be more extreme than other combat sports. We sought to investigate the magnitude of weight lost and prevalence of weight loss strategies in different combat sports. Methods: Competitors (n=637) from Brazilian jiu jitsu (BJJ), boxing, judo, MMA, muay Thai/kickboxing (MT/K), taekwondo (TKD) and wrestling completed an online questionnaire seeking information regarding their weight loss practices. Results: Body mass manipulation was commonly undertaken by all combat sports athletes, with a particularly high incidence of gradual dieting, increased exercise and fluid restriction. Skipping meals was higher in TKD and wrestling (84%) compared with the other combat sports (~58%), whilst training in heated rooms and forced oral fluid loss (spitting) was higher in wrestling (83% and 47%, respectively) compared with other combat sports (~45% and ~19%, respectively). MMA athletes reported the highest usage of sauna (76%) and water loading (67%) whilst also reporting the second highest use of training in rubber/plastic suits (63%). Conclusions: Body mass manipulation was present in all combat sports with the prevalence and magnitude of acute weight loss greater in MMA. The incidence of and practices reported will assist support staff to be fully aware of the variety of methods these athletes and coaches may use to achieve weight loss. Additionally, the results could aid regulatory bodies in the further development of policies on weight cutting.

Barley et al., (2018)[b]	Yes	This study sought to determine the influence of acute dehydration on physical performance and3 physiology in Mixed Martial Arts (MMA). MMA athletes (n=14; age: 23±4 years), completed in4 a randomized counterbalanced order a dehydration protocol, (DHY: 3 h cycling at 60 W in 40°C5 to induce 5% dehydration) or thermoneutral control (25°C: CONT) exercise, followed by ad6 libitum fluid/food intake. Performance testing (a repeat sled push test, medicine ball chest throw7 and vertical jump) was completed 3 and 24 h following the intervention, while urine and blood8 samples were collected before, 20 min, 3 and 24 h following the intervention. Body mass was9 reduced (4.8±0.8%) following DHY (p<0.001) and remained lower than CONT at 3 and 24 h10 post (p=0.003 and p=0.024, respectively). Compared to CONT average sled push times were11 slower 3 and 24 h following DHY (19±15%; p=0.001; g=1.229 and 14±15%; p=0.012; g=0.671,12 respectively). When compared to the CONT hand grip was weaker 3 h following DHY (53±813 and 51±8 kg; p=0.044, g=0.243 respectively) and medicine ball chest throw distances were14 shorter 24 h following DHY (474±52 and 449±44 cm; p=0.016, g=0.253 respectively). No15 significant differences were observed in vertical jump (p=0.467). Urine specific gravity was16 higher than CONT 20 min (p=0.035) and 24 h (p=0.035) following DHY. Acute dehydration of17 4.8% body mass results in reduced physical performance 3 and 24 h following. There is need for18 caution when athletes use dehydration for weight loss 24 h prior to competition.

Barley et al., (2018)[c]	Yes	Introduction/Purpose: This study examined the influence of acute dehydration onneuromuscular function. Methods: On separate days, combat sports athletes experienced in acute dehydrationpractices (n = 14) completed a 3 h passive heating intervention (40_C, 63% relativehumidity) to induce dehydration (DHY) or a thermoneutral euhydration control (25_C,50% relative humidity: CON). In the ensuing 3 h ad libitum fluid and food intake wasallowed, after which participants performed fatiguing exercise consisting of repeatedunilateral knee extensions at 85% of their maximal voluntary isometric contraction(MVIC) torque until task failure. Both before and after the fatiguing protocol participantsperformed six MVICs during which measures of central and peripheral neuromuscular function were made. Urine and whole blood samples to assess urine specific gravity,urine osmolality, hematocrit and serum osmolality were collected before, immediatelyand 3 h after intervention. Results: Body mass was reduced by 3.2 _ 1.1% immediately after DHY (P < 0.001)but recovered by 3 h. Urine and whole blood markers indicated dehydration immediatelyafter DHY, although blood markers were not different to CON at 3 h. Participants completed 28% fewer knee extensions at 85% MVIC (P < 0.001, g = 0.775) and reported a greater perception of fatigue (P = 0.012) 3 h after DHY than CON despite peak torque results being unaffected. No between-condition differences were observed in central or peripheral indicators of neuromuscular function at any timepoint. Conclusion: Results indicate that acute dehydration of 3.2% body mass followed by3 h of recovery impairs muscular strength-endurance and increases fatigue perceptionwithout changes in markers of central or peripheral function. These findings suggestthat altered fatigue perception underpins muscular performance decrements in recovery from acute dehydration.

Bernick et al., (2021)	No	Objectives: Determine, through video reviews, how often concussions occur in combat sport matches, what influence they have on the outcome, and how well non-physician personnel can be trained to recognize concussions.<br>Methods: This is a retrospective video analysis by an 8-person panel of 60 professional fights (30 boxing and 30 mixed martial arts). Through video review, physician and non-physician personnel recorded details about each probable concussion and determined if and when they would have stopped the fight compared to the official stoppage time.<br>Results: A concussion was recorded in 47/60 fights. The mean number of concussions per minute of fight time was 0.061 (0.047 for boxers and 0.085 for MMA). When stratifying by outcome of the bout, the mean number of concussions per minute for the winner was 0.010 compared to the loser at 0.111 concussions per minute. The fighter that sustained the first concussion ultimately lost 98% of the time. The physician and non-physician raters had high agreement regarding the number of concussions that occurred to each fighter per match. The physician raters judged that 24 of the 60 fights (11 boxing [37%]; 13 MMA [43 %]) should have been stopped sooner than what occurred.<br>Conclusion: Recognizing that the concussions often occur in combat sport matches, that the losing fighter almost always is concussed first and tends to sustain more concussions during the fight, along with the demonstration that non-physician personnel can be taught to recognize concussion, may guide policy changes that improve brain health in combat sports.

Buse (2006)	No	Objective: To identify the most salient medical issues that may be associated with mixed martial arts competition by determining the types and proportions of match stoppages. Methods: Publicly available video footage of 1284 men competing in 642 consecutive televised matches from November 1993 to November 2003 was reviewed to determine the reasons for which matches were stopped. Matches were sanctioned by either a United States or Japan based mixed martial arts organisation. Results: Of the 642 matches, 182 (28.3¡3.4%) were stopped because of head impact, 106 (16.5¡2.9%) because of musculoskeletal stress, 91 (14.1¡2.7%) because of neck choke, 83 (12.9¡2.6%) because of miscellaneous trauma, 173 (27.0¡3.4%) because of expiration of match time, and seven (1.0¡0.8%) because of disqualification, where the values in parentheses are percentages¡95% confidence interval. Conclusions: Blunt force to the head resulted in the highest proportion of match stoppages. Further research is warranted to delineate the morbidity associated with participation in mixed martial arts.
Bledsoe et al., (2006)	No	Mixed Martial Arts (MMA) competitions were introduced in the United States with the first Ultimate Fighting Championship (UFC) in 1993. In 2001, Nevada and New Jersey sanctioned MMA events after requiring a series of rule changes. The purpose of this study was to determine the incidence of injury in professional MMA fighters. Data from all professional MMA events that took place between September 2001 and December 2004 in the state of Nevada were obtained from the Nevada Athletic Commission. Medical and outcome data from events were analyzed based on a pair-matched case-control design. Both conditional and unconditional logistic regression models were used to assess risk factors for injury. A total of 171 MMA matches involving 220 different fighters occurred during the study period. There were a total of 96 injuries to 78 fighters. Of the 171 matches fought, 69 (40.3%) ended with at least one injured fighter. The overall injury rate was 28.6 injuries per 100 fight participations or 12.5 injuries per 100 competitor rounds. Facial laceration

		was the most common injury accounting for 47.9% of all injuries, followed by hand injury (13.5%), nose injury (10.4%), and eye injury (8.3%). With adjustment for weight and match outcome, older age was associated with significantly increased risk of injury. The most common conclusion to a MMA fight was a technical knockout (TKO) followed by a tap out. The injury rate in MMA competitions is compatible with other combat sports involving striking. The lower knockout rates in MMA compared to boxing may help prevent brain injury in MMA events.
Bodden et al., (2015)	No	This study assessed the basic fundamentalmovements of mixed martial arts (MMA) athletes using the functional movement screen (FMS) assessment and determined if an intervention program was successful at improvingresults. Participants were placed into 1 of the 2 groups: intervention and control groups. The intervention group wasrequired to complete a corrective exercise program 4 times per week, and all participants were asked to continue their usual MMA training routine. A mid-intervention FMS test was included to examine if successful results were noticed sooner than the 8-week period. Results highlighted differences in FMStest scores between the control group and intervention group = 0.006). Post hoc testing revealed a significant increase inthe FMS score of the intervention group between weeks 0 and8 (p = 0.00) and weeks 0 and 4 (p = 0.00) and no significant increase between weeks 4 and 8 (p = 1.00). A x2 analysis revealed that the intervention group participants were morelikely to have an FMS score .14 than participants in the controlgroup at week 4 (x2 = 7.29, p , 0.01) and week 8 (x2 =5.2, p # 0.05). Finally, a greater number of participants in theintervention group were free from asymmetry at week 4 andweek 8 compared with the initial test period. The results of thestudy suggested that a 4-week intervention program was sufficient at improving FMS scores. Most if not all, the movements covered on the FMS relate to many aspects of MMA training.The knowledge that the FMS can identify movement

		dysfunctionsand, furthermore, the fact that the issues can be improved through a standardized intervention program could be advantageous to MMA coaches, thus, providing the opportunity to adapt and implement new additions to training programs.
Brandt et al., (2018)	No	Mixed martial arts (MMA) fighters typicallyuse rapid weight loss (RWL) as a strategy to make competitionweight. The aim of the present study was to compare bodyweight and mood changes in professional male MMA athleteswho used strategies to rapidly lose weight (n = 9) and withMMA athletes who did not (n = 3). Body mass and mood statesof anger, confusion, depression, fatigue, tension, and vigor and total mood disturbance were assessed (a) 30 days before competition,(b) at the official weigh-in 1 day before competition, (c)10 minutes before competition, and (d) 10 minutes post competition.Results indicated that RWL associated with reporting higherconfusion and greater total mood disturbance at each assessmentpoint. Rapid weight loss also associated with high anger at theofficial weigh-in. However, in performance, RWL did not havedeleterious effects on performance. The RWL group also reportedgreater total mood disturbance at all assessment points witha moderate difference effect size. Research supports the notion that RWL associates with potentially dysfunctional mood states.

Bray et al., (2021)	No	Traumatic brain injury (TBI) is a common source of functional impairment among athletes, military personnel, and the general population. Professional fighters in both boxing and mixed martial arts (MMA) are at particular risk for repetitive TBI and may provide valuable insight into both the pathophysiology of TBI and its consequences. Currently, effects of fighter weight class on brain volumetrics (regional and total) and functional outcomes are unknown. Fifty-three boxers and 103 MMA fighters participating in the Professional Fighters Brain Health Study (PRBHS) underwent volumetric magnetic resonance imaging (MRI) and neuropsychological testing. Fighters were divided into lightweight (£139.9 lb), middleweight (140.0–178.5 lb), and heavyweight (>178.5 lb). Compared with lightweight fighters, heavyweights displayed greater yearly reductions in regional brain volume (boxers: bi- lateral thalami; MMA: left thalamus, right putamen) and functional performance (boxers: processing speed, simple and choice reaction; MMA: Trails A and B tests). Lightweights suffered greater reductions in regional brain volume on a per-fight basis (boxers: left thalamus; MMA: right putamen). Heavyweight fighters bore greater yearly burden of regional brain volume and functional decrements, possibly related to differing fight dynamics and force of strikes in this division. Lightweights demonstrated greater volumetric decrements on a per-fight basis. Although more research is needed, greater per-fight decrements in lightweights may be related to practices of weight- cutting, which may increase vulnerability to neurodegeneration post-TBI. Observed decrements associated with weight class may result in progressive impairments in fighter performance, suggesting interventions mitigat- ing the burden of TBI in professional fighters may both improve brain health and increase professional longevity.

Brechney et al., (2019)	No	Weight cutting is common among amateur and professional mixed martial arts (MMA)competitors because of the belief that it provides an advantage in combat sports. This studyaimed to identify whether fight outcome(win vs. loss vs. type of loss) was influenced by magnitudes of body mass (BM) lost through weight cutting and BM regained beforethe fight after official weigh-in in amateur and professional MMA athletes with previous weight-cutting experience. Body mass datawere collected using self-report from 75 MMA athletes (59 amateur and 16 professional) before commencing weight-cutting practices 7 days before weigh-in, by the regulating body at their official weigh-in 24 hours before the fight and through direct measurement immediately before competition. Data were analyzed according to win; loss by technical knockout or knockout (KO);loss by submission; or loss by the judge’s decision. Athletes who lost their fight cut significantly more BM (10.6%) compared with athletes who won (8.6%) (p 5 0.04, d 5 0.48, 95% confidence interval [CI] 5 0.02–0.93), but there were no differences betweentypes of loss. There were no significant differences in recoveredBMbetween athletes who won (6.8%) vs. lost (7.4%), or type of loss.Furthermore, there was a significant relationship between greater magnitudes of BM cut and greater likelihood of losing the fight(B 5 20.12, P 5 0.048), odd ratio 0.89 (95% CI: 0.79–1.00). This study provides the first line of evidence that excessive weightcutting may be detrimental to fight outcome in MMA.
Brito et al., (2018)	Yes	Mixed Martial Arts (MMA) is a combat sport that requires maximum physicaleffort during competitions. In this context, some athletes can use illicit substancesin order to improve their performance. By means of paired analysis, the present studycompared the motor actions of athletes who had failed an anti-doping test versus theirperformance in combat against a winner or loser without doping presence. For this, 267rounds (male and female) were analyzed in professional matches. The rounds were pairedby athletes in the conditions: doping, winning and losing. Motor actions were analyzedthrough a specific and previously validated

		protocol. Of the substances detected, anabolic androgenic steroids represented 55% (p≤0.001). Doped athletes had lower pause time(83.4Å}68.3 vs. 131.7Å}95.2, p≤0.001) and longer time at high intensity (85.2Å}86.6 vs.51.2Å}73.3, p=0.002) compared to the losing condition. Regarding the technical-tacticalanalysis in standing combat, winning presented a higher mean compared to doping inall variables except for Knockdowns (p=0.08), single body strikes landed (p=0.15), singleleg strikes landed (p=0.25) and single strike attempts (p=0.4). In conclusion, athletes whotested positive presented higher performance in the physical variables (effort and pausetime) in comparison to the losing condition; however, doping did not reflect in better technical-tactical performance.
Camarço et al., (2016)	No	Background and Aim:Rapid weight loss (RWL) is extensively practiced by combat sports athletes, in-cluding Mixed Martial Arts (MMA), but its effects on performance are not well established with different magnitudes of RWL, including those higher than 5% of total body weight. The aim of the present study was to follow MMA athletes during RWL with subsequent weight regain to evaluate the responses of isometric strength, power, cognition and salivary nitrite (NO2–) content. Methods: Two professional male MMA fighters, same age, competing in the same weight category under-went two magnitudes of RWL before a simulated competition period. Anthropometric measures, records of nutritional status, training, voluntary dehydration strategies, salivary samples, cognition response, isometric strength and muscular power were obtained: (I) 7 days before combat, (II) at the weigh-in moment, and (III) in the combat day. Results and Conclusions: Athlete 1 lost 7.2 kg (9.1% of total bodyweight) and Athlete 2 lost 4.0 kg (5.3% of total bodyweight). Athlete 1 had a lower and misbalanced caloric ingestion (708 ± 428 kcal), ingested 6 L of water during the first 5 days of RWL, underwent 2 days of fasting, water and sodium restriction before weigh-in. Athlete 2 was supervised by a nutritionist, had a balanced diet (1600 ± 0 kcal), ingested 2 L of water during the first 6 days of RWL, underwent only 1 day of

		fasting and water restriction, and did not restrict sodium. As expected, there was a negative effect of RWL in the evaluated parameters at the weigh-in moment, while in the combat day, salivary NO2– was not completely reestablished at baseline levels (decreased by 35.9% in Athlete 1 and, 25.2% in Athlete 2, as compared with 7 days before). The athlete who underwent a lower weight loss (5.3%) presented better recovery of cognition and upper limbs power on the combat day as compared with the athlete who lost 9.1% of body weight. Although we cannot precisely conclude, this case report led us to believe that the recovery period between weigh-in and competition may be insufficient for total reestablishment of salivary NO2– after RWL, and higher amounts of RWL have negative impacts on average power and cognition when compared with lower RWL. Relevance for patients: Scientific aspects related with performance in MMA athletes brought to light the absence of studies investigating the recovery of isometric strength, power, cognition and salivary NO2–during RWL with subsequent weight regain. This study revealed that athletes from the same categories can adopt different magnitudes of weight loss, and that this procedure impacts several important measures, for example, the reduction of salivary NO2– is associated with the lower O2 transport capacity, decreasing muscle performance.
Chen & Cheesman., (2013)	Yes	This study investigated whether mental toughness distinguishes mixed martial arts (MMA) athletes competing at different levels. It was theorized that higher mental toughness would separate those competing at the professional level compared to lower levels. Male MMA competitors (N = 136, M age = 27.1 yr.,SD = 4.8) were categorized as amateur, semi-professional, or professional and assessed by questionnaire using the Psychological Performance Inventory–A andthe Sports Mental Toughness Questionnaire. There were statistically significant differencesbetween the three groups on mental toughness. The professional group had higher scores compared to semi-professional and

		amateur groups with regard to confidence, positive cognition, and determination. The findings supported previous work that athletes performing at higher levels have superior mental toughness.
Cherepkova et al., (2019)	No	Objectives: VNTR polymorphisms of DRD4 and DAT genes were studied in the Russian andChechen men convicted of crimes, and two control groups comprised of the MMA fighters and asample of general population. A group of MMA fighters included only the subjects withouthistory of antisocial behavior.Methods: DNA was isolated by phenol–chloroform extraction from the blood. GenotypingVNTR polymorphisms of the DRD4 and DAT genes were performed by PCR on publishedmethods.Results: Among those convicted of felonies and most grave crimes, carriers of DRD4 long alleles are found more frequently, similarly to the cohort of MMA fighters (lacking criminalrecord in both paternal lines). The 9/9 DAT genotype carriers are more frequently encountered among the habitual offenders. A frequency of the combination of the DRD4 genotype 4/7 andDAT genotype 10/10 is clearly higher among the convicts of violent crimes and the MMAfighters. One can speculate the presence of a “controlled aggression” without a predisposition to pathological violence in the MMA fighters.Conclusions: Our study supports the hypothesis of genetic predisposition to different variants ofextreme behavior mediated by genetic determinants involved in the functioning of neuromediatorsystems including those controlling dopamine pathways.

Chernozub et al., (2018)	No	Purpose – to develop models for Mixed Martial Arts athletes power training, depending on the predominance of the strike orthe wrestling style in fighting, and also to determine the impact of the proposed training loads on increasing the functional abilityof their bodies.Methods. We examined 30 athletes aged 20-22 who were involved in Mixed Martial Arts fights over the last 2 years. Half the fightersuse the strike style in the course of combat, and the rest specialize in the wrestling style. To assess the effectiveness of the occupationmodels we developed, we used the control testing method of the level of power capabilities development. With the help ofthe biochemical control of cortisol concentrations in the blood serum of the fighters, we determined the manifestation of adaptive-compensatory reactions of the body to various power loads.Results: It was established that the optimal power loads for fighters using the strike style of fighting was to use a high-intensityregime when working with an a lactate or lactate energy supply system. In turn, the most effective power loads, for the maximumrealisation of functional potential in athletes prioritizing the wrestling style during the fight, was the use of low-intensity regimeswith a large amount of work in the glycolytic power supply system.Conclusions. The analysis of the results obtained during the experiment demonstrates the need for using models of training sessionsdeveloped in the process of power training of MMA fighters, taking into account the particular fighting style.
Chernozub et al., (2019)	No	Purpose: to identify the optimal load parameters in the process of power training of Mixed Martial Arts athleteswith a wrestling style on the basis of studying adaptive and compensatory body reactions in conditions of intense muscular activity of different intensity and volume of work. Material: We examined 40 sportsmen aged (21 Å}0.8), who participated in Mixed Martial Arts fights during the last (2.0 Å} 0.3) years and used predominantly awrestling style of fighting. The athletes were divided into 2 groups: group A and B. The participants of group Aused the low-intensity load and the large amount of work in the training process.

		Athletes of group B used loadsof high intensity and small volume of work. Morphometric, biochemical, and statistical methods of researchwere used to study the features of adaptive and compensatory body reactions of these athletes in conditions ofpower training of different intensity. Results: The obtained results show that athletes of group A increased theircircumference indices by 1.9% on average after 3 months of research. In group B, for the same period of time,the studied indices increased by 5.5% on average compared to the baseline data. It was noted that at thebeginning of the study, the cortisol concentration in blood serum of group A athletes decreased after exercises by23.9% in comparison with the basal ones. This fact indicates compensatory reactions to the stimulus and highenergy costs. We observed completely opposite changes in group B sportsmen. They were expressed in theincrease of the studied indicator by 46% in comparison with the data recorded before exercises. This fact testifiesto the adequacy of the loads to the functional body capabilities of this group of athletes. The results ofbiochemical control of the changes in the hormone testosterone and the enzyme lactate dehydrogenase in theblood of both groups' athletes confirm the fact that it is the use of high-intensity loads that promotes theenhancement of the athletes' adaptive body capabilities for training activities in MMA. Conclusions: Theanalysis of the results obtained during the series of experimental studies indicates the necessity of correcting loadparameters during power training. It should be based on the analysis of athletes' adaptive and compensatorybody reactions to a stress stimulus. Otherwise there should be developed completely new training programs.

Connor & Egan., (2019)	Yes	Abstract: Rapid weight loss (RWL) is frequently practiced in weight category sports, including MixedMartial Arts (MMA). The aim of the present study was to describe self-reported methods of RWL ina sample of competitive MMA athletes comprising of both amateur and professional fighters. Thepreviously validated Rapid Weight Loss Questionnaire, with the addition of questions on waterloading and hot salt baths, was completed anonymously online by athletes (n = 30; all male, n = 15/15professional/amateur) from MMA clubs around Dublin, Ireland. All but one (97%) of the athletes surveyed lost weight in order to compete, with the average weight loss being 7.9% _ 3.1% of habitualbody mass. The RWL score (mean _ SD) for this sample was 37.9 _ 9.6, and a tendency for higher[6.0 (95%CI; 1.1, 13.1) (p = 0.093; d = 0.64)] RWL scores for professional (40.8 _ 8.9) compared toamateur (34.8 _ 9.6) athletes was observed. Frequencies of "always" or "sometimes" were reported as 90% for water loading, 76% for hot salt baths and 55% for 24 h of fasting. Fellow fighters (41%) andcoaches/mentors (38%) were "very influential" on RWL practices of these athletes, with doctors (67%),dietitians (41%), and physical trainers (37%) said to be "not influential". RWL is highly prevalent inMMA across both amateur and professional athletes, and RWL scores are higher than other combatsports. Water loading and hot salt baths are amongst the most commonly used methods of RWLdespite little research on these methods for body mass reduction or ects on performance inweight category sports.

Connor, Shelley & Egan., (2020)	Yes	Hot water immersion, known as a hot bath, is used by MMA athletes to produce rapid weight loss (RWL) bymeans of passive fluid loss. This study investigated the magnitude of bodymass losses using a standardizedhot bath protocol with or without the addition of salt. In a crossover design, elevenmaleMMA athletes (28.5± 4.6 y; 1.83 ± 0.07 m; 82.5 ± 9.1 kg) performed a 20-min immersion at 37.8°C followed by a 40-min wrap in awarm room. This bath and wrap was performed twice per visit. During one visit, only fresh water was used(FWB), and in the other visit, magnesium sulphate (1.6% wt/vol) was added to the bath (SWB). Prior to eachvisit, 24 h of carbohydrate, fibre, and fluid restriction was undertaken as part of the RWL protocol. Body masslosses induced by the hot bath protocols were 1.63 ± 0.75 kg and 1.60 ± 0.80 kg for FWB and SWB,respectively, and equivalent to ~2.1% body mass. Under the conditions employed, the magnitude of bodymass loss in SWB was similar to FWB. However, further research should explore bathing in a temperaturethat is consistent with that habitually used by fighters, and/or higher concentrations of salt.
Connor & Egan., (2021)	Yes	Hot water immersion is used by athletes in weight category sports to produce rapid weight loss (RWL) by means of passive fluid loss, and often is performed with the addition of Epsom salts (magnesium sulphate). This study investigated the magnitude of body mass losses during hot water immersion with or without the addition of salt, with the temperature commencing at 37.8°C and being self-adjusted by participants to their maximum tolerable temperature. In a crossover design, eight male MMA athletes (29.4 ± 5.3 y; 1.83 ± 0.05 m; 85.0 ± 4.9 kg) performed a 20 min whole-body immersion followed by a 40 min wrap in a warm room, twice in sequence per visit. During one visit, only fresh water was used (FWB), and in the other visit, magnesium sulphate (1.6% wt/vol) was added to the bath (SWB). Prior to each visit, 24 h of carbohydrate, fibre and fluid restriction was undertaken. Water temperatures at the end of the first and second baths were ~39.0°C and

		~39.5°C, respectively. Body mass losses induced by the hot bath protocols were 1.71 ± 0.70 kg and 1.66 ± 0.78 kg for FWB and SWB, respectively (P = 0.867 between trials, d = 0.07), and equivalent to ~2.0% body mass. Body mass lost during the entire RWL protocol was 4.5 ± 0.7%. Under the conditions employed, the magnitude of body mass lost in SWB was similar to FWB. Augmenting passive fluid loss during hot water immersion with the addition of salt may require a higher salt concentration than that presently utilised.
Coswig et al., (2015)	No	The purpose of this study was to compare biochemical and hormonal responses between mixed martial arts(MMA) competitors with minimal prefight weight loss and those undergoing rapid weight loss (RWL). Bloodsamples were taken from 17 MMA athletes (Mean± SD ; age: 27.4 ±5.3yr; body mass: 76.2 ± 12.4kg; height:1.71 ± 0.05m and training experience: 39.4 ± 25 months) before and after each match, according to the official events rules. The no rapid weight loss (NWL, n =12) group weighed in on the day of the event (~30 min priorfight) and athletes declared not having used RWL strategies, while the RWL group (n = 5) weighed in 24 hrbefore the event and the athletes claimed to have lost 7.4 ± 1.1kg, approximately 10% of their body mass in theweek preceding the event. Results showed significant ($p < .05$) increases following fights, regardless of group,in lactate, glucose, lactate dehydrogenase (LDH), creatinine, and cortisol for all athletes. With regard to groupdifferences, NWL had significantly ($p < .05$) greater creatinine levels (Mean± SD ; pre to post) (NWL= 101.6± 15–142.3 ± 22.9μ mol/L and RWL= 68.9 ± 10.6–79.5 ± 15.9μ mol/L), while RWL had higher LDH (median[interquartile range]; pre to post) (NWL= 211.5[183–236] to 231[203–258]U/L and RWL= 390[370.5–443.5]to 488[463.5–540.5]U/L) and AST (NWL= 30[22–37] to 32[22–41]U/L and 39[32.5–76.5] to 72[38.5–112.5]U/L) values (NWL versus RWL, $p < .05$). Post hoc analysis showed that AST significantly increased in onlythe RWL group, while creatinine increased in only the

		NWL group. The practice of rapid weight loss showeda negative impact on energy availability and increased both muscle damage markers and catabolic expressionin MMA fighters.
Coswig et al., (2016)[a]	Yes	Simulated matches are a relevant component of training for mixed martial arts (MMA) athletes. This study aimed to char- acterize time-motion responses and investigate physiological stress and neuromuscular changes related to MMA sparring matches. Thirteen athletes with an average age of 25 6 5 years, body mass of 81.3 6 9.5 kg, height of 176.2 6 5.5 cm, and time of practice in MMA of 39 6 25 months partici- pated in the study. The fighters executed three 5-minute rounds with 1-minute intervals. Blood and salivary samples were collected and physical tests and psychometric question- naires administered at 3 time points: before (PRE), immediately after (POST), and 48 hours after the combat (48 h). Statistical analysis applied analysis of variance for repeated measure- ments. In biochemical analysis, significant changes (p # 0.05) were identified between PRE and POST (glucose: 80.3 6 12.7 to 156.5 6 19.1 mg$ml21; lactate: 4 6 1.7 to 15.6 6 4.8 mmol$dl21), POST and 48 hours (glucose: 156.5 6 19.1 to 87.6 6 15.5 mg$ml21; lactate: 15.6 6 4.8 to 2.9 6 3.5 mmol$dl21; urea: 44.1 6 8.9 to 36.3 6 7.8 mg$ml21), and PRE and 48 hours (creatine kinase [CK]: 255.8 6 137.4 to 395.9 6 188.7 U/L). In addition, time- motion analyses showed a total high:low intensity of 1:2 and an effort:pause ratio of 1:3. In conclusion, simulated MMA sparring matches feature moderate to high intensity and a low degree of musculoskeletal damage, which can be seen by absence of physical

		performance and decrease in CK. Re- sults of the study indicate that sparring training could be intro- duced into competitive microcycles to improve technical and tactical aspects of MMA matches, due to the high motor spec- ificity and low muscle damage.
Coswig et al., (2016)[b]	Yes	Background: One of the goals for training in combat sports is to mimic real situations. For mixed martial arts (MMA), simulatedsparring matches are a frequent component during training, but a there is a lack of knowledge considering the differences in sparringand competitive environments.Objectives: The main objective of this study was to compare biochemical responses to sparring and official MMA matches.Materials and Methods: Twenty five male professionalMMAfighters were evaluated during official events (OFF = 12) and simulatedmatches (SIM = 13). For both situations, blood samples were taken before (PRE) and immediately after (POST) matches. For statisticalanalysis, two-way analysis of variance (time x group and time x winner) were used to compare the dependent parametric variables.For non-parametric data, the Kruskal-Wallis test was used and differences were confirmed by Mann-Whitney tests.Results: No significant differences were observed among the groups for demographic variables. The athletes were 26.5 _ 5 yearswith 80 _ 10 kg, 1.74 _ 0.05 m and had 39.4 _ 25 months of training experience. Primary results indicated higher blood glucoseconcentration prior to fights for OFF group (OFF= 6.1 _ 1.2 mmol/L and SIM= 4.4 _ 0.7 mmol/L; P < 0.01) and higher ALT values forOFF group at both time points (OFF: PRE = 41.2 _ 12 U/L, POST = 44.2 _ 14.1 U/L; SIM: PRE = 28.1 _ 13.8 U/L, POST = 30.5 _ 12.5 U/L; P =0.001). In addition, the blood lactate showed similar responses for both groups (OFF: PRE= 4 [3.4 - 4.4] mmol/L, POST= 16.9 [13.8 - 23.5]mmol/L; SIM: PRE = 3.8 [2.8 - 5.5] mmol/L, POST= 16.8 [12.3 - 19.2] mmol/L; P < 0.001).Conclusions: In conclusion, MMA official and simulated matches

		induce similar high intensity glycolytic demands and minimalchanges to biochemical markers of muscle damage immediately following the fights. Glycolytic availability prior to the fights wasraised exclusively in response to official matches.
Coswig et al., (2019)	Yes	We aimed to describe the nutritional and behavioral strategies for rapid weight loss (RWL), investigate the effects of RWL and weight regain (WRG) in winners and losers and verify mood state and technical-tactical/time-motion parameters in Mixed Martial Arts (MMA). The sample consisted of MMA athletes after a single real match and was separated into two groups: Winners (n=8, age: 25.4±6.1yo., height: 173.9±0.2cm, habitual body mass (BM): 89.9±17.3kg) and Losers (n=7, age: 24.4±6.8yo., height: 178.4±0.9cm, habitual BM: 90.8±19.5kg). Both groups exhibited RWL and WRG, verified their macronutrient intake, underwent weight and height assessments and completed two questionnaires (POMS and RWL) at i) 24 h before weigh-in, ii) weigh-in, iii) post-bout and iv) during a validated time-motion and technical-tactical analysis during the bout. Variance analysis, repeated measures and a logistic regression analysis were used. The main results showed significant differences between the time points in terms of total caloric intake as well as carbohydrate, protein and lipid ingestion. Statistical differences in combat analysis were observed between the winners and losers in terms of high-intensity relative time [58(10;98) s and 32(1;60) s, respectively], lower limb sequences [3.5(1.0;7.5) sequences and 1.0(0.0;1.0) sequences, respectively], and ground and pound actions [2.5(0.0;4.5) actions and 0.0(0.0;0.5) actions, respectively], and logistic regression confirmed the importance of high-intensity relative time and lower limb sequences on

		MMA performance. RWL and WRG strategies were related to technical-tactical and time-motion patterns as well as match outcomes. Weight management should be carefully supervised by specialized professionals to reduce health risks and raise competitive performance.
Curran-Sills & Abedin (2018)	No	Background There is limited literature that examinesrisk factors for injury and mild traumatic brain injury (mTBI)in mixed martial arts (MMA). An examination of previouslystudied bout and athlete characteristics that may pose health risks while partaking in this sport is warranted. Hypothesis/purpose To determine the incidence ofinjury and concussion, along with the identification of riskfactors that contribute to injury and mTBI in amateur andprofessional MMA bouts in Calgary, Alberta. Study design A retrospective cohort study with case–control design. Methods Calgary amateur and professional MMA recordswere examined from 1 January 2010 to 31 December2015. Descriptive statistics were used to describe theincidence of injury and concussion, along with univariate and multivariable logistic regression to identify risk factorsfor injury and mTBI. Results The injury rate per 100 athlete exposure (AE),the injury rate per 100 min of exposure and the concussionrate per 100 AE were 23.6 (95% CI 20.5 to 27.0), 4.1(95% CI 3.48 to 4.70) and 14.7 (95% CI 11.8 to 17.2),respectively. The most common location of injury wasthe head and mTBI was the most common type of injury.Athletes whose bout was finished by a knockout/technical knockout, corner stoppage, draw, no contest or physician,and those whose country of origin was non-Canadian,were more likely to sustain an injury. No risk factors forconcussion were shown to be significant. Conclusion Engaging in MMA exposes athletes toinherent risk and several recommendations are

		proposedto reduce these risks. Future prospective investigations arenecessary to better delineate the findings in this study.
Dal Bello et al., (2019)	Yes	This study compared grappling motor actions of male mixed martial arts (MMA) athletes considering outcometypes from Ultimate Fighting Championship (UFC) bouts. A validated protocol of technical-tactical analysis was utilizedas in previous studies addressing MMA performance analysis, and Kruskall Wallis and U Mann-Whitney tests wereapplied to compare effects of types of outcome decisions (Split vs. Unanimous Decision vs. Knockout-KO/Technical knockout-TKO vs. Submission). Unanimous Decision showed higher frequencies of takedowns attempted/round thanKO/TKO and Submission outcomes ($p \leq 0.05$; 1.9 ± 1.9 vs. 1.3 ± 1.4 vs. 1.0 ± 1.1 attempts). Bouts with Split Decisiondemonstrated higher takedowns/round than bouts ended by Submission ($p = 0.048$; 0.4 ± 0.7 vs. 0.2 ± 0.6 attempts).TKO/KO showed lower values of sweeps/round ($p = 0.008$, 0.0 ± 0.0 vs. 0.1 ± 0.3 attempts) and takedowns attempted/round ($p = 0.014$, 1.3 ± 1.4 vs. 2.0 ± 1.6 attempts) than bouts ending by Split Decision. The Submission outcome showed a higher frequency of submissions attempted/round than KO/TKO and Unanimous Decision ($p \leq 0.041$,0.3 ± 0.7 vs 0.2 ± 0.5 vs 0.2 ± 0.5). These results show a large specificity in the type of grappling attack/situation according to the strategy to end the combat. These results also show that the grappling strategy and tactics are variable dependingon the strengths and weaknesses of the athletes, and can be used by coaches and athletes to develop specific training programs.

De Souza et al., (2017)	No	The aim of this study was to analyze the serum concentrations of testosterone (T), cortisol (C), lactate (LAC), creatine kinase (CK) and glucose (GLU) on *mixed martial arts* (MMA) athletes, before and after a fight. We divided 20 MMA athletes into two groups of 10 fighters each, according to the result of a fight, and were then evaluated four times: 24 hours before (-24h), one hour before (-1h), immediately after (0h) and 24 hours after the fight (+24h). It was observed: a significant decrease in T and T/C between moment -24h and 0h and a subsequent increase between the moment 0h and +24h and a reverse behaviour in variables C, LAC and GLU ($p<0.0001$); a decrease in CK between moment -24h and -1h and an increase between moment -1h and +24h ($p<0.0001$); and differences between winners and losers T levels, in moments -24h, -1h, 0h and +24h ($p = 0.009$ e $p < 0.001$, $p = 0.005$ e $p = 0,001$, T and C, respectively), in T/C in the moments -24h and 0h ($p=0.006$ and $p=0.001$, respectively) and in GLU levels ($p<0.0001$) in the moment 0h. Therefore, it seems that an MMA fight leads to metabolic stress and muscle damage, regardless of the result of the fight. The coaches have now more biochemical and hormonal references and indicators in response to an MMA fight.
Del Vecchio (2011)	No	*Summary.* —Mixed martial arts (MMA) have become a fast-growing worldwide expansion of martial arts competition, requiring high level of skill, physical conditioning, and strategy, and involving a synthesis of combat while standing or on the ground. This study quantified the effort-pause ratio (EP), and classified effort segments of stand-up or groundwork development to identify the number of actions performed per round in MMA matches. 52 MMA athletes participated in the study (*M* age = 24 yr., *SD* = 5; average experience in MMA = 5 yr., *SD* = 3). A one-way analysis of variance with repeated measurements was conducted to compare the type of action across the rounds. A chi-squared test was applied across the percentages to compare proportions of different events. Only one significant difference ($p < .05$) was observed among rounds: time in groundwork of low intensity was longer in the

		second compared to the third round. When the interval between rounds was not considered, the EP ratio (between high-intensity effort to low-intensity effort plus pauses) was 1:2 to 1:4. This ratio is between ratios typical for judo, wrestling, karate, and taekwondo and reflects the combination of ground and standup techniques. Most of the matches ended in the third round, involving high-intensity actions, predominantly executed during groundwork combat.
Dixson et al., (2018)	Yes	In contemporary human societies, where direct male-male competition is reduced compared to ancestral socie- ties, sporting competitions remain an avenue for status acquisition via intra-sexual competition. Beards are the most visually salient and sexually dimorphic of men's secondary sexual traits and play a strong role in commu- nicating masculinity, dominance and aggressiveness intra-sexually. Hypotheses have been advanced that beards provide advantages in intra-sexual combat, as protective organs and honest signals of fighting ability. Here we provide the first test of these hypotheses using data from professional mixed martial arts fighters competing in the Ultimate Fighting Championship. We explored whether secondary sexual traits (height, weight, bearded- ness), fighting stance (southpaw, orthodox), arm reach and past contest experiences impact on contest out- comes. If beards function as protective organs, bearded fighters should succumb to fewer knock-outs, and hence protection to injuries to the jaw, fewer abrasions and lacerations to the face and brain damage than clean-shaven fighters. Alternatively, if beards signal fighting ability then bearded fighters should win more fights. We found no evidence that beardedness was associated with fewer losses by knock-out or greater fighter ability. While fighters with longer reaches won more fights, neither stance nor past experience influenced fight out- comes. We suggest that beards represent dishonest signals of formidability that may serve to curtail the escala- tion of intra-sexual

		conflict through intimidation rather than providing advantages in direct combat.
Dos Santos et al., (2018)	Yes	This study verified the performance probabilities by mixed martial arts (MMA) rounds by the same athletes, doing paired comparisons of time-motion and actions before and after 10-years. The sample was composed of 845 UFC rounds of 45 athletes separated in before (M1, age range: 34-44 yrs.) and after (M2, age range: 44-54 yrs.). Motor control (takedowns, submissions, chokes, locks, strike actions to the head, body and leg attempted and landed) and time-motion (high and low intensities; standing and ground times) analysis were done. The main results showed significant differences ($p \leq 0.05$) in total strikes landed [M1: 22(13;34) > M2: 18(10;31.7)]; total strikes attempted [M1: 41(24.5;62) > M2: 35(21;48)], single head strikes attempted [M1: 19(9;34.5) > M2: 16.5(9;28)], single body strikes landed [M1: 1(0;4) > M2: 1(0;2)], single body strikes landed [M1: 2(0;5) > M2: 1(0;3)], takedowns attempted [M1: 1(0;2) > M2: 1(0;2)], standing combat time (M1: 2:10.28±1:38.95 > M2: 1:55.56±1:32.17) and low intensity time (M1: 2:11.45±1:38.95 > M2: 1:56.26±1:31.89). Variables, which increased the probability to be associated with over the years, were body strikes landed, head strikes landed, total strikes landed and single strikes attempted, while body strikes attempted, head strikes attempted, total strikes attempted and submission attempted had a negative association with MMA time experience. Therefore, M2 athletes should be focused on stand combat moment combined with strikes landed actions, targeting the head – it has the highest potential performance probability

		and avoid unsuccessful body strike attempts and submissions – it has the lowest potential performance probability over 10 years.
Fares et al., (2019)	No	Objectives: Mixed martial arts (MMA) has witnessed a surge in popularity worldwide. This studyexplores the musculoskeletal and head injuries sustained in the professional fights of the UltimateFighting Championship (UFC), and establishes associations between injury profiles and impactfull contributory factors.Methods: The Nevada State Athletic Commission database was screened for ringside physician reportsof UFC fights between January 2016 and July 2018. Information on the fighter's gender, weight, injury,way of finish, and match result were collected. Injury rates were calculated, and statistical analyses wereconducted to determine significant associations among variables. P-values <0.05 were considered significant (95% CI).Results: A total of 291 injuries were recorded in 285 fights from nine weight divisions. The overall injuryrate was 51 per 100 athletic exposures (AE). Males predominantly partook in 249 matches (87%) andhad higher injury rates (54 injuries per 100 AE) than females (30 injuries per 100 AE). Decision was themost common way a match ended. Knockouts (KOs) were significantly higher in males (36%) than infemales (14%, P = 0.0007). Submissions were significantly higher in females (36%) than in males (16%,P = 0.001). Head injuries (67%) were the most common injuries reported with a rate of 34 per 100 AE.Upper limb injuries were significantly higher in females (40%) than in males (14%, P = 0.0003). Lowerlimb injuries were significantly higher in males (19%) than in females (5%, P = 0.01). Head injuries weresignificantly associated with KOs (P < 0.0001). Upper limb injuries (P = 0.032) and lower limb injuries(P

		= 0.034) were significantly associated with matches that ended with Decision. Trend-line analyses howed that as weight division increases, overall injury rates, head injuries, lower limb injuries, and KOs'frequency increase, whereas upper limb injuries, Submission frequency, and Decision frequencydecrease.Conclusion: MMA has a high injury rate. Gender, way of finish, and weight play an important role inpredicting fight outcomes and injury profiles. Injury prevention policies must be entertained to limit injury risk in MMA.
Fares et al., (2021)[a]	No	Objective: Mixed-Martial-Arts (MMA) has witnessed a rapid growth over the recent years. This study aims to explore the patterns and trends of head injuries in MMA. Design: Descriptive epidemiological study. Setting: Ringside physician reports of the Ultimate Fighting Championship (UFC) fights between 2016 and 2019 (inclusive) were screened. Data were extracted from the Nevada State Athletic Commission (NSAC) database. Play-by-play video analysis was also conducted. Participants: UFC fighters involved in fights sanctioned by the NSAC, between 2016 and the end of 2019 (N = 816). Independent variables: Sex, location of head injury, type of head injury, injury mechanism, number of significant head strikes, type of finish, and weight division. Main outcome measures: Head injury rates were calculated. A one-way analysis of variance (ANOVA) was used to explore any statistically significant differences between injury rates of different locations, types, and types of finishes. An independent t-test was used to determine whether any significant differences existed between the two sexes, and a Joinpoint regression analysis was used to determine the statistical significance of the trends of head injury rates across different weight divisions. P-values <0.05 were considered significant (95% CI). Results: A total of 288 head injuries in 408 fights were recorded during our study period. Head injury rate constituted 35 injuries per 100 athletic-exposures (AE) in sanctioned fights. Traumatic brain injuries (TBI) were the most common type of injury, with a rate of 16 per 100AE, significantly

		greater than that of fractures (p = 0.003). Males had a head injury rate of 37 per 100AE, higher than that of females which was 23 per 100AE. Technical Knockout (TKO)/ Knockout(KO) was the type of finish with the highest rate of head injuries, significantly greater than that of decision or submission (p < 0.001). In general, head injury rates were higher as weight divisions increased. Conclusion: Head injuries are prevalent in MMA. Preventive measures need to be implemented to ensure fighter safety and limit injury risk.
Fares et al, (2021)[b]	No	Doping is a practice that is present in many sports and organizations, including mixed martial arts and the Ultimate Fighting Championship (UFC). The aim of this study is to explore the epidemiological patterns of doping among UFC athletes. **Methods:** We screened the official United-States-Anti-Doping-Agency® (USADA) website, the annual USADA reports and the official UFC website for information on fighters and anti-doping rule violations (ADRVs). Our dataset included gender, age, weight class, testing numbers, date of ADRV, type of ADRV and duration of suspension. Appropriate statistical tests were conducted to assess for statistical significance. **Results:** USADA tested 1070 UFC athletes 2,624 times as of late 2015 up till the end of 2019 (N=1070). A total of 209 adverse findings were recorded; out of which, 102 ADRVs were committed by 93 athletes (8.7%) from all weight divisions. This constituted an adverse finding rate of 16.55 per 1000 test and an ADRV rate of 8.08 per1000 test. Mean age of sanctioned athletes was 32 years. Use of anabolic steroids was signficantly the most common ADRV recorded (p=0.018). The men's heavyweight division had an ADRV rate of 19.3 per 1000 tests, significantly higher than that of women's bantamweight (p=0.03), women's featherweight (p=0.009), and men's flyweight divisions (p=0.035). ADRV rate showed a significantly increasing trend among men's weight divisions (p<0.001).

Faro, Lima-Junior & Machado., (2021)	Yes	We aimed to analyze whether rapid weight gain (RWG) between the official weigh-in and the time of the fight was associated with fight success in MMA. A total of 700 professional MMA fights involving 1,400 weigh-ins from 21 MMA promotions regulated by the California State Athletic Commission were analyzed. Multilevel logistic regression accounting for individual (i.e., athlete) and cluster levels (i.e., fights) was used to analyze the association of all measures with a theoretical relationship with the dependent variable and without interdependency with one another (i.e., %RWG, sex, body mass division, competition level) with the fight outcome (i.e., win or loss). The odds ratios (OR) with 95% confidence intervals (95%CI) were calculated. The highest mean %RWG was found for the flyweight, bantamweight, featherweight, and lightweight divisions. The %RWG significantly predicted the fight outcome (ß=0.044; OR=1.045; 95%CI=1.014–1.078; p=0.005) so that for each 1% of additional RWG, the chance of winning increased by 4.5%. With the largest sample to date and in a 'real-world' scenario, the present results suggest that the magnitude of RWG is linked to the chance of winning in MMA combats. It is suggested that regulatory commissions, confederations, and event organizers should consider regulating RWG, considering that, despite its detrimental impact on the athletes' health and performance, the potential advantage might stimulate athletes to invest in rapid weight loss, followed by gain after the official weigh-in to increase their chance of winning.

Fernandes et al., (2018)	Yes	This study compared the injury incidence, motor actions and the time-motion of combat before and after thechanges in Mixed Martial Arts (MMA) rules. The sample was composed of 3,538 male matches, with 1,756rounds prior to 2011, and 1,802 after the 2012 rule changes. The analyses showed higher frequency after rulechanges of total strike attempts (41.5±25.9 and 43.6±26.4 for Before vs. After; p=0.03), single strike attempts(31.0±22.8 and 34.8±24.8 for Before vs. After; p≤0.001), single head strike attempts (24.1±19.6 and 27.4±21.5for Before vs. After; p≤0.001), single body strike attempts (4.0±4.2 and 4.4±4.3 for Before vs. After; p≤0.001),single strike landed (13.3±11.1 and 14.7±11.0 for Before vs. After; p≤0.001), single head strike landed (8.1±8.5and 9.2±8.3 for Before vs. After; p≤0.001), and single leg strike landed (2.3±3.15 and 2.5±3.2 for Before vs.After; p=0.02). There was a higher exposure time after the changes 383.3 vs. 480.2; for Before vs. After,p=0.019). Furthermore, there was a higher frequency of bouts ended in the last round (5.) after the rule changes(90 vs. 150; p=0.006). Our results showed higher exposure time and frequency of technical-tactical actionscorrelated with injury incidence in professional MMA.
Follmer et al., (2019)	No	Background: Brain injury arising from head trauma is a major concern in mixed martial arts (MMA) because knockout (KO) and technical knockout (TKO) are frequent fight outcomes. Previous studies have shown a high incidence of matches ending due to strikes to the head but did not consider weight categories and female fights. This study aimed at analyzing match stoppages in MMA and the exposure to head trauma distinguished by sex and weight categories. Hypothesis: The heavier the weight class, the greater the risk and incidence of head trauma will be, regardless of sex. Study Design: Descriptive epidemiology study. Level of Evidence: Level 3. Methods: Publicly available data of 167 MMA events from 1903 fights between 2014 and 2017 were assessed, comprising 8 male and 2 female weight categories.Results: The combined KO/TKO rates per 100 athlete-exposures in the middleweight (19.53), light heavyweight

		(20.8), and heavyweight (26.09) divisions were greater than previously reported for MMA. While stoppage via KO/TKO occurred in 7.9% of combats in the female strawweight division, it occurred in 52.1% of the male heavyweight fights. The male middleweight (P = 0.001), light heavyweight (P < 0.001), and heavyweight divisions (P < 0.001) had an increased risk of KO/TKO due to strikes to the head by 80%, 100%, and 206%, respectively. The risk in the flyweight division decreased 62% (P = 0.001). All categories were compared with the lightweight division. The female bantamweight category presented a 221% increased risk in matches ending due to KO/TKO compared with the strawweight division (P = 0.012). Punches to the head were the major technique used to end a combat via KO/TKO, regardless of sex and weight class. Conclusion: Head injury risk and incidence varies considerably according to sex and weight category in MMA.
Follmer, Andreato & Coswig., (2021)	No	Background. Submissions due to joint locks and strangleholds accounted for many stoppages in the early days of Mixed Martial Arts (MMA). Since then, the adoption of unified weight classes, and rule changes have considerably impacted the sport. There is a need to update the database of the incidence of submission outcomes in modern MMA. Problem and Aim. Assess ratios of match-ending by submission as well as to identify the most frequently-used submission tech- niques among different weight and gender categories in MMA. Methods. A total of 1903 fights (1728 male and 175 female) in 167 events from 2014 to 2017 were assessed. Submissions were fur- ther investigated to identify the specific technique used to end a fight. Results. Submissions accounted for the ending in 17.3% of male and 21.1% of female modern MMA combats, respectively. As the weight class became lighter, the chance of a stoppage due to submission is greater (p<0.05). The more frequent combat-ending techniques were based on strangleholds, mainly rear and front chokes. The elbow was the most targeted joint for all weight classes, except the male heavyweight. The shoulder was the second most attacked joint,

		whereas the knee and the ankle were reported in a relatively small number. Conclusion. This updated database on fight outcomes should be considered for MMA trainers, coaches and athletes when pro- gramming specific training sessions aiming to mimic combat patterns.
Fliotsos et al., (2021)	No	Purpose: To describe the frequency and type of eye injuries in fighters in mixed martial arts (MMA) competition.Methods: Fight result data were collected from the Nevada Athletic Commission database from 2001 to 2020. Any fighters in a professional mixed martial arts (MMA) contest with an eye injury were included. Main outcome measures included frequency and rate of eye injuries per fight and the types of eye injuries. Secondary outcome measures were gender, laterality, decision type, and length of no-contact recommended. Results: Of the 256 MMA events in the database, 187 events (73.3%) had at least one eye injury. Of a total 2208 fights at these events, there were 363 fighters who sustained 369 eye injuries, with the yearly rate of eye injuries per 100 fighters ranging from 2.56 to 12.22. The most common injuries were eyebrow and eyelid lacerations (n=160, 43%), lacerations around the eye (n=98, 27%), and orbital fractures (n=62, 17%). Most eye injuries were right sided (n=197, 53.3%) and the majority of fighters with eye injuries lost their match (n=228, 62.8%). Fifty-seven fighters were recommended for further ophthalmology clearance after the match. The most common reasons for recommended ophthalmology follow-up was orbital fracture (n=25, 44%) and retinal injury (n=7, 12%). Forty-three fighters received no- contact requirements relating to their injury for an average of 8.9 weeks (range 1–24 weeks). Conclusion: Ophthalmic injuries in professional MMA were

		prevalent, were most often lacerations surrounding the eye, and often accompanied the fighter losing their match.
Ghoul et al., (2019)	Yes	This study investigates the physiological/physical responses to a simulated mixedmartial arts (MMA) competition over 24 hr. Twelve fighters performed a simulatedMMA competition, consisting of three 5-min MMA matches. Physiological/physicaldata were assessed before (Trest), directly after round 1 (Trd1), round 2 (Trd2) andround 3 (Trd3), and then 30-min (Trecovery30min) and/or 24-hr (Trecovery24h) postcompetition.Heart rate (HR), rating of perceived exertion (RPE) and blood lactateconcentration ([La-]) were assessed at Trest, Trd1, Trd2 and Trd3. Biological data werecollected at Trest, Trd3, Trecovery30min and Trecovery24h. Physical tests were performed atTrest, Trecovery30min and Trecovery24h. HR, RPE and [La-] were high during competition.Leukocytes, hemoglobin, total protein and glycemia were increased at Trd3compared with all other time points ($p<0.05$). Cortisol was increased at Trd3compared with Trest and Trecovery24h ($p<0.05$). Testosterone was higher at Trd3 andTrecovery30min than Trest ($p<0.001$). Higher values of uric acid were noted duringrecovery periods ($p<0.001$). Lactate dehydrogenase was lower at Trest comparedwith Trd3, Trecovery30min and Trecovery24h ($p<0.05$). Countermovement jump was higher atTrest than Trecovery30min ($p=0.020$). Consequently, MMA is a high-intensity intermittent combat sport that induces significant fatigue and muscle damage, both of which arestill present 24-hr post-competition.

Giboin & Gruber (2019)	Yes	Mixed martial arts (MMA) is a full-contact sport whose popularity and professionalism are rapidly growing.However, the specific physiological demands of this sport have been only scarcely studied so far, and especially the amount or typeof neuromuscular fatigue induced by an MMA bout remains completely unknown. We estimated neuromuscular fatigue of kneeextensors muscles during and after an MMA training protocol designed to simulate thephysiological demands of MMA competitionin competitive practitioners (n 5 9) with isometric maximal voluntary force (MVF), potentiated muscle twitch at rest (Ptw), andvoluntary activation (VA). Bayesian linear mixed models showed that the training protocol induced a reduction of MVF, Ptw, and VA.Although the largest reduction across time of VA was smaller than the largest reduction of Ptw, an effect of VA, but not of Ptw, wasfound on MVF variation. The training protocol induced neuromuscular fatigue, with a larger peripheral (Ptw) than central component(VA). However, despite the large decrease in Ptw, force production capacity was related only to VA, indicating that central controlmight play an important role in the compensation of the peripheral fatigue components estimated with Ptw. This central compensationcan most probably prevent a too large loss of muscle force during the training protocol.
Gomes-Santos et al., (2022)	Yes	Combat sports involve a combination of strenuous physical activity, usually at the anaerobic threshold, followed by intermittent low-intensity recovery periods for energy re-establishment. Oxidative stress and inflammation are inevitable exercise-related processes that could drastically affect athletic performance and practitioners' health, unless efficiently con- trolled during and after physical activities. This study aims to measure oxidative stress and inflammation biomarkers in the plasma of 12 top ranked professional Mixed Martial Arts (MMAs) athletes before and after simulated combats under official rules (pre-post study). Our results show that the athletes exhibited mild oxidative imbalances in plasma, evidenced by significant (p , 0.01) higher contents

		of both reduced (+7.3%) and oxidized glutathione (+28%), uric acid (+21%), and “free” iron (+21%) after combat, whereas variation tendencies (0.05 , p , 0.01) were observed in the antioxidant capacity in plasma (240%), and SOD (227%) or GPX (+20%) antioxidant activities in erythrocytes. However, a clear pro-inflammatory state was detected by increases in circulating cytokines IL-6 (+6,020%), IL-1b (+4,357%), and tumor necrosis factor alpha (+63%), and by an abrupt drop of the anti-inflammatory cytokine IL-10 (298%). A significant correlation was observed between pre-post variations of IL-6 and GSH/GSSG ratio in plasma (p , 0.0001), which reinforces the integration between oxidative stress and inflammation during MMA combats. Considering metabolic and mechanical stresses (imposed by combat techniques, e.g., punches and joint locks), this study indicates pre-existing inflammation, although minor oxidative stress, in MMA professionals after combat.
Heath & Callahan (2013)	No	Mixed martial arts (MMA) is a full-contact, fighting sport that hasrisen in popularity over recent years, resulting in an increasein both training facilities and sport participants. To date, littleresearch has examined the complications and vulnerability to headtrauma, or concussive symptomatology, in MMA athletes. In thisstudy, we assessed relationships between training routines andconcussive symptoms, as well as medical care, in MMA athletes.A sample (N = 119) of MMA athletes reported concussive symptoms,training routines, and medical histories through an online survey.Nearly 15% of the MMA athletes reported history of a knockout, andnearly one-third reported a technical knockout. Subjective ratingsof concussive symptoms were high for these athletes, with manyof them waiting only a brief time after such incidents to returnto competition. These findings have important implications forinforming the medical treatment and safety decision for returningto participation for these athletes.

Hillier et al., (2019)	Yes	The practice of rapid weight loss in mixed martial arts (MMA) is an increasing concern but data remains scarce. The aim of this study was to investigate the prevalence, magnitude, methods and influencers of rapid weight loss in professional and amateur MMA athletes. MMA athletes (n=314; 287 men, 27 women) across nine weight categories (strawweight to heavyweight), completed a validated questionnaire adapted for this sport. Sex-specific data were analysed, and sub-group comparisons were made between athletes competing at professional and amateur levels. Most athletes purposefully reduced body weight for competition (men: 97.2%; women: 100%). The magnitude of rapid weight loss in one week prior to weigh-in was significantly greater for professional athletes compared to those competing at amateur level (men: 5.9% v 4.2%; women: 5.0% v 2.1% of body weight; $p<0.05$). In the 24 h preceding weigh-in, the magnitude of rapid weight loss was greater at professional than amateur level in men (3.7% v 2.5% of body weight; $p<0.05$. Most athletes 'always' or 'sometimes' used water loading (72.9%), restricting fluid intake (71.3%) and sweat suits (55.4%) for rapid weight loss. Coaches were cited as the primary source of influence on rapid weight loss practices (men: 29.3%, women: 48.1%). There is a high reported prevalence of rapid weight loss in MMA, at professional and amateur levels. Our findings, constituting the largest enquiry to date, call for urgent action from MMA organizations to safeguard the health and wellbeing of athletes competing in this sport.

Hubbard et al., (2019)	No	The aim of this prospective cohort study was to determine the effect of an 'event,' defined as a knockout(KO), technical knock-out (TKO), choke, or submission, on King-Devick (K-D) test times in mixedmartial arts (MMA) athletes. MMA athletes (28.3 ± 6.6 years, n = 92) underwent K-D testing prior to andfollowing a workout or match. Comparison of baseline and post-workout/match K-D times to assess anysignificant change. K-D tests worsened (longer) in a majority of athletes following an 'event' (N = 21)(49.6 ± 7.8 s vs 46.6 ± 7.8 s, p = 0.0156, Wilcoxon signed-rank test). K-D tests improved (shorter)following a standard workout or match in which no 'event' occurred in a majority of cases (n = 69) (44.2± 7.2 s vs 49.2 ± 10.9 s, p = <0.0001, Wilcoxon signed-rank test). Longer duration (worsening) of postmatchK-D tests occurred in most athletes sustaining an 'event'; K-D tests shortened (improved) in a majority of athletes not sustaining an 'event'. Our study suggests MMA athletes suffering an 'event' mayhave sustained a brain injury similar to a concussion.
Hutchison et al., (2014)	No	Background: Mixed martial arts (MMA) is a full combative sport with a recent global increase in popularity despite significant scrutiny from medical associations. To date, the empirical research of the risk of head injuries associated with this sport is limited.Youth and amateur participation is growing, warranting investigation into the burden and mechanism of injuries associated withthis sport.Purpose: (1) To determine the incidence, risk factors, and characteristics of knockouts (KOs) and technical knockouts (TKOs)from repetitive strikes in professional MMA; and (2) to identify the mechanisms of head trauma and the situational factors that lead to KOs and TKOs secondary to repetitive strikes through video analysis.Study Design: Descriptive epidemiology study.Methods: Competition data and video records for all KOs and TKOs from numbered Ultimate Fighting Championship MMAevents (n = 844) between 2006 to 2012. Analyses included (1) multivariate logistic regression to investigate factors associatedwith an increased risk of sustaining a KO or TKO secondary to repetitive strikes

		and (2) video analysis of all KOs and TKOs secondaryto repetitive strikes with descriptive statistics.Results: During the study period, the KO rate was 6.4 per 100 athlete-exposures (AEs) (12.7% of matches), and the rate of TKOs secondary to repetitive strikes was 9.5 per 100 AEs (19.1% of matches), for a combined incidence of match-ending head trauma of 15.9 per 100 AEs (31.9% of matches). Logistic regression identified that weight class, earlier time in a round, earlier round ina match, and older age were risk factors for both KOs and TKOs secondary to repetitive strikes. Match significance and previously sustained KOs or TKOs were also risk factors for KOs. Video analysis identified that all KOs were the result of direct impactto the head, most frequently a strike to the mandibular region (53.9%). The average time between the KO-strike and match stoppagewas 3.5 seconds (range, 0-20 seconds), with losers sustaining an average of 2.6 additional strikes (range, 0-20 strikes) to thehead. For TKOs secondary to strikes, in the 30-second interval immediately preceding match stoppage, losers sustained, onaverage, 18.5 strikes (range, 5-46 strikes), with 92.3% of these being strikes to the head.Conclusion: Rates of KOs and TKOs in MMA are higher than previously reported rates in other combative and contact sports.Public health authorities and physicians should be cognizant of the rates and mechanisms of head trauma. Preventive measuresto lessen the risks of head trauma for those who elect to participate in MMA are described.
Ignatjeva et al., (2021)	No	The main aim of this study is to analyze the influence of the external load on the reaction time of the lower limbs of mixed martial arts fighters. The research group consisted of 31 senior MMA fighters (with training experience of at least 10 years, divided into two weight categories: light (66-70 kg) and heavy (over 93 kg) and each of them into two categories: intermediate and advanced). The Keizer Leg Press pneumatic device (Keizer, Fresno, CA, USA) was used to evaluate the reaction time of the lower limbs of MMA fighters. The subjects performed a 20-minute warm-up and then worked on the Keizer Leg Press measuring device according

		to the manufacturer's protocol. Based on this data, a 10 single repetitions test was performed. The value of the external resistance and the time to the next repetition were not changed to obtain the measurement data. The obtained results indicate a constant deterioration of the value of the RT_{LBL} and RT_{LL} variable when increasing the external load in both studied groups. In the case of advanced players in the 66-70 kg weight group, a clear (statistically significant) increase in the value of the RT_{LBL} and RT_{LL} variable occurred from 70% 1RM, while in the intermediate group it was from 50% 1RM, and from 80% 1RM in the weight category above 93 in both study groups. Growth dynamics was lower in the advanced group in both tested weight categories. Performing the progression of the external load may allow the detection of the dominance of one lower limb and determination of the dynamics of differences in the reaction time of the frontal and back limb in MMA fighters.
James et al., (2017)[a]	Yes	Objectives:To determine those performance indicators that have the greatest influence on classifying outcome at the elite level of mixed martial arts (MMA). A secondary objective was to establish the efficacy of decision tree analysis in explaining the characteristics of victory when compared toalternate statistical methods.Design: Cross-sectional observational.Methods: Eleven raw performance indicators from male Ultimate Fighting Championship bouts(n=234) from July 2014–December 2014 were screened for analysis. Each raw performance indicator was also converted to a rate-dependent measure to be scaled to fight duration. Further, three additionalperformance indicators were calculated from the dataset and included in the analysis. Cohen's d effectsizes were employed to determine the magnitude of the differences between Wins and Losses, whiledecision tree (chi-square automatic interaction detector (CHAID)) and discriminant function analyses(DFA) were used to classify outcome (Win and Loss).Results: Effect size comparisons revealed differences

		between Wins and Losses across a number ofperformance indicators. Decision tree (raw: 71.8%; rate-scaled: 76.3%) and DFA (raw: 71.4%; ratescaled71.2%) achieved similar classification accuracies. Grappling and accuracy performanceindicators were the most influential in explaining outcome. The decision tree models also revealed multiple combinations of performance indicators leading to victory.Conclusions: The decision tree analyses suggest that grappling activity and technique accuracy are ofparticular importance in achieving victory in elite-level MMA competition. The DFA resultssupported the importance of these performance indicators. Decision tree induction represents anintuitive and slightly more accurate approach to explaining bout outcome in this sport when compared to DFA.
James et al., (2017)[b]	Yes	Purpose: To determine whether the maximal strength, impulse and power characteristics of competitive mixed martial arts (MMA) athletes differ according to competition level. Methods: Twenty-nine male semi-professional and amateur MMA competitors were stratified into either higher-level (HL) or lower-level (LL) performers on the basis of competition grade and success. The one-repetition maximum (1RM) squat was used to assess lower body dynamic strength, while a spectrum of impulse, power, force and velocity variables were evaluated during an incremental load jump squat. Additionally, participants performed an isometric mid-thigh pull (IMTP) and 1RM bench press to determine whole-body isometric force and upper body dynamic strength capabilities, respectively. All force and power variables were expressed relative to body mass (BM). Results: The HL competitors produced significantly superior values across a multitude of measures. These included 1RM squat strength (1.84 ± 0.23 vs 1.56 ± 0.24 kg.BM-1; P=0.003), in addition to performance in the incremental load jump squat that revealed greater peak power (P=0.005-0.002), force (P=0.002-0.004) and velocity (P=0.002-0.03) at each load. Higher measures of impulse (P=0.01-0.04) were noted in a number of conditions. Average power (P=0.002-0.02)

		and velocity (*P*=0.01-0.04) at all loads in addition to a series of rate-dependent measures were also superior in the HL group (*P*=0.005-0.02). The HL competitors' 1RM bench press values approached significantly greater levels (*P*=0.056), while IMTP performance did not differ between groups. Conclusions: Maximal lower body neuromuscular capabilities are key attributes distinguishing HL from LL MMA competitors. This information can be used to inform evidenced-based training and performance monitoring practices.
James et al., (2018)	Yes	This investigation sought to determine the relevance of anaerobic and aerobic-based measures to competition level andbout outcome in mixed martial arts competitors. For the primary analysis, seven higher-level and eight lower-level malemixed martial arts competitors were compared across a series of short-term anaerobic (sprints at 10 and 20 m),repeated maximal effort (repeated sprint ability), and intermittent aerobic tests (Yo-Yo Intermittent RecoveryLevel 2)). For the secondary analysis, data were then pooled so relationships could be explored between test performanceand percentage of bouts reaching a decision. Cohen's d effect sizes and qualitative magnitude-based inferences werecalculated to describe the differences between groups. These same descriptors were used to interpret the output of theregression analysis used to predict decision percentage. Superior performances by the higher-level group were revealedacross most variables to a non-trivial magnitude. Furthermore, it was likely that a decrease in short-term anaerobicperformance or an increase in intermittent endurance capacity positively related to an increased likelihood of bouts lasting the full scheduled duration. These findings indicate the importance of anaerobic and aerobic qualities to mixedmartial arts performance and combat methods.

James et al., (2020)	Yes	Gross countermovement jump (CMJ) performance measures aregreater in higher-level mixed martial arts (MMA) competitors than lower-level (LL) competitors. Differences in CMJ kinetics andkinematics throughout the action may explain those CMJ performance differences, but this remains to be investigated. After warmupand familiarization, 27 MMA competitors (divided into 2 groups based on competitive standard; higher level [HL]: n514 and LL:n 5 13) completed 3 maximal effort CMJs. Power, force, velocity, displacement-time waveforms and eccentric phase displacement,eccentric time, eccentric impulse, and the modified reactive strength index (RSImod) were compared between groups usingstatistical parametric mapping procedures and independent t-tests. Power (between 65 and 71% of the power-time curve) wasgreater in the HL than that of the LL group (p50.01) despite no differences in eccentric displacement (p50.50) or movement time(p50.17) between groups. The HL group demonstrated a greater RSImod (p50.05) alongside a reduced eccentric time (p50.02)and eccentric impulse (p 5 0.02). These findings suggest that timing and control of lower-body force production contributed tobetween-group differences in CMJ performance among MMA competitors.
Jansen et al., (2021)	No	Background: The accumulation of subconcussive impacts has been implicated in permanent neurological impairment. A gap in understanding the relationship between head impacts and neurological function is the lack of precise characterization and quantification of forces that individuals experience during sports training and competition. Purpose: To characterize impact exposure during training and competition among male and female athletes participating in boxing and mixed martial arts (MMA) via an instrumented custom-fit Impact Monitoring Mouthguard (IMM). Study Design: Cross-sectional study; Level of evidence, 3. Methods: Twenty-three athletes (n 1⁄4 4 women) were provided a custom-fit IMM. The IMM monitored impacts during sparring and competition. All training and competition sessions were

		videotaped. Video and IMM data were synchronized for post hoc data verification of true positives and substantiation of impact location. IMM data were collected from boxing and MMA athletes at a collaborating site. For each true-positive impact, peak linear acceleration and peak angular acceleration were calculated. Wilcoxon rank sum tests were used to evaluate potential differences in sport, activity type, and sex with respect to each outcome. Differ- ences in impact location were assessed via Kruskal-Wallis tests. Results: IMM data were collected from 53 amateur training sessions and 6 competitions (session range, 5-20 minutes). A total of 896 head impacts (men, n 1⁄4 786; women, n 1⁄4 110) were identified using IMM data and video verification: 827 in practice and 69 during competition. MMA and boxers experienced a comparable number of impacts per practice session or competition. In general, MMA impacts produced significantly higher peak angular acceleration than did boxing impacts (P < .001) and were more varied in impact location on the head during competitions. In terms of sex, men experienced a greater number of impacts than women per practice session. However, there was no significant difference between men and women in terms of impact magnitude. Conclusion: Characteristic profiles of head impact exposure differed between boxing and MMA athletes; however, the impact magnitudes were not significantly different for male and female athletes.
Jetton et al., (2013)	No	The purpose of this study was to characterize the magnitude of acute weight gain (AWG)and dehydration in mixed martial arts (MMA) fighters before competition. Urinary measures of hydration status and bodymass were determined approximately 24 hours before and then again approximately 2 hours before competition in 40 MMAfighters (mean 6 SE, age: 25.2 60.65 years, height: 1.77 60.01 m, body mass: 75.8 6 1.5 kg). The AWG was defined asthe amount of body weight the fighters gained in the approximately22-hour period between the official weigh-in and theactual competition. On average, the MMA fighters gained3.40 6 2.2 kg or 4.4% of their body weight in the approximately22-hour period

		before competition. Urinespecific gravity significantlydecreased (p , 0.001) from 1.028 6 0.001 to 1.020 60.001 duringthe approximately 22-hour rehydration period.Results demonstrated that 39% of the MMAfighters presentedwith a Usg of .1.021 immediately before competition indicatingsignificant orserious dehydration. The MMA fighters undergo significant dehydration and fluctuations inbody mass (4.4%avg.) in the 24-hour period before competition. Urinary measuresof hydrationstatus indicate that a significant proportion ofMMA fighters are not successfully rehydratingbefore competitionand subsequently are competing in a dehydrated state. Weightmanagement guidelines to prevent acute dehydration in MMA fighters are warranted to preventingnecessary adversehealth events secondary to dehydration.
Ji (2016)	No	Abstract. [Purpose] The purpose of the present study was to examine the types of injuries associated with mixedmartial arts and their location in order to provide substantial information to help reduce the risk of these injuriesduring mixed martial arts. [Subjects and Methods] Data were collected from 455 mixed martial arts athletes whopracticed mixed martial arts or who participated in mixed martial arts competitions in the Seoul MetropolitanCity and Gyeongnam Province of Korea between June 3, 2015, and November 6, 2015. Questionnaires were usedto collect the data. The convenience sampling method was used, based on the non-probability sampling extractionmethod. [Results] The arm, neck, and head were the most frequent locations of the injuries; and lacerations, concussions,and contusions were the most frequently diagnosed types of injuries in the mixed martial arts athletes in thisstudy. [Conclusion] Reducing the risk of injury by establishing an alert system and preventing critical injuries byincorporating safety measures are important.
Kasper et al., (2019)	No	The aim of the present case study was to quantify the physiological and metabolic impact of extreme weight cutting by an elite male MMA athlete. Throughout an 8-week period, we obtained regular assessments of body composition, resting metabolic rate (RMR), VO2peak and blood

		clinical chemistry to assess endocrine status, lipid profiles, hydration and kidney function. The athlete adhered to a "phased" weight loss plan consisting of 7 weeks of reduced energy (ranging from 1300 – 1900 kcal.d-1) intake (phase 1), 5 days of water loading with 8 L per day for 4 days followed by 250 ml on day 5 (phase 2), 20 h fasting and dehydration (phase 3) and 32 h of rehydration and refueling prior to competition (phase 4). Body mass declined by 18.1 % (80.2 to 65.7 kg) corresponding to changes of 4.4, 2.8 and 7.3 kg in phase 1, 2 and 3, respectively. We observed clear indices of relative energy deficiency, as evidenced by reduced RMR (-331 kcal), inability to complete performance tests, alterations to endocrine hormones (testosterone: <3 nmol.L-1) and hypercholesterolemia (>6 mmol.L-1). Moreover, severe dehydration (reducing body mass by 9.3%) in the final 24 hours prior to weigh-in induced hypernatremia (plasma sodium: 148 mmol.L-1) and acute kidney injury (serum creatinine: 177 μmol.L-1). These data therefore support publicized reports of the harmful (and potentially fatal) effects of extreme weight cutting in MMA athletes and represent a call for action to governing bodies to safeguard the welfare of MMA athletes.
Kirk et al., (2015)	Yes	Tri-axial accelerometry has previously been shown to provide a reliable technique for the assessment of MMA specific activities during isolated training movements. However, this method has yet to be used for the analysis of competition demands. Six male MMA trained participants took part in a single simulated MMA bout each comprising of 3 rounds of 5minutes with 1 minute rest between rounds. Participants wore a tri-axial accelerometer positioned between the T3-T4 vertebrae in a harness. Mean accumulated player load (PLdACC) and mean accumulated player load per minute (PLdACC.min-1) were calculated for each round and for the bout overall. Capillary blood lactate samples were also taken prior to warm up,post warm up, upon completion of each round and 5 minutes post boutwhilst video based time motion analysis was completed post hoc. PLdACCwas 224.32 ± 26.59 au, whilst PLdACC.min-1 was 14.91 ±

		1.78 au.Participants had a mean post bout lactate of 9.25 ± 2.96 mmol.L whilstthere was significant differences in lactate levels across all six sampling points. The group was found to have a work rest ratio (W:R) of 1:1.01whilst a significant difference was found between bout winners and bout losers in terms of the amount of successful takedowns.
Kirk et al., (2021)	Yes	The aim of this study was to quantify typical training load and periodisation practices of MMA athletes. MMA competitors (n = 14; age = 22.4 ± 4.4 years; body mass = 71.3 ± 7.7 kg; stature = 171 ±9.9 cm) were observed during training for 8 consecutive weeks without inter- vention. Seven athletes were training for competitive bouts whilst the remaining 7 were not. Daily training duration, intensity (RPE), load (sRPE and segRPE), fatigue (short question- naire of fatigue) and body region soreness (CR10 scale) were recorded. Using Bayesian analyses (BF_{10}�3), data demonstrate that training duration (weekly mean range = 3.9–5.3 hours), sRPE (weekly mean range = 1,287–1,791 AU), strain (weekly mean range = 1,143– 1,819 AU), monotony (weekly mean range = 0.63–0.83 AU), fatigue (weekly mean range = 16–20 AU) and soreness did not change within or between weeks. Between weeks monot- ony (2.3 ± 0.7 AU) supported little variance in weekly training load. There were no differ- ences in any variable between participants who competed and those who did not with the except of the final week before the bout, where an abrupt step taper occurred leading to no between group differences in fatigue. Training intensity distribution corresponding to high, moderate and low was 20, 33 and 47%, respectively. Striking drills accounted for the largest portion of weekly training time (20–32%), with MMA sparring the least (2–7%). Only striking sparring and wrestling sparring displayed

		statistical weekly differences in duration or load. Athletes reported MMA sparring and wrestling sparring as high intensity (RPE�7), BJJ spar- ring, striking sparring and wrestling drills as moderate intensity (RPE 5–6), and striking drills and BJJ drills as low intensity (RPE�4). We conclude that periodisation of training load was largely absent in this cohort of MMA athletes, as is the case within and between weekly microcycles.
Kostikiadis et al., (2018)	Yes	The purpose of the present study was to investigate the effect ofa short-term, high-intensity, low-volume Mixed Martial Arts(MMA) specific strength and conditioning training program onperformance in national level MMA athletes. Seventeen experiencedfighters were divided into two groups: (A) Specific TrainingGroup (STG; n = 10), which followed a specific strength andconditioning program designed according to the demands ofMMA competition and (B) Regular Training Group (RTG; n =7), in which participants followed a regular strength and conditioningprogram commonly used by MMA athletes. Before andafter the four-week training period (3 days per week), body composition,aerobic fitness, strength, power and speed were evaluated.Significant improvements in estimated VO2max, averagepower during the 2000 m rowing, bench press, back squat anddeadlift 1RM, SJ power, CMJ height power, medicine ball throwvelocity, 10 m sprint and 2 m take down speed and fat-free masswere found only in the STG (3.7 to 22.2%; $p < 0.05$; Hedge's g =-0.42 – 4.1). No significant changes were found for the RTG (p =0.225 to 0.811). Significant differences between the groups werefound for almost all post-training assessments ($p < 0.05$; Hedge'sg = 0.25 – 1.45) as well as for the percentage changes from pre topost training ($p < 0.05$; Hedge's g: 0.25 – 1.45). Significant relationshipswere found between percentage changes in fat-freemass, endurance capacity, muscle strength/power and speed (r: -

		0.475 to 0.758; $p < 0.05$). These results suggest that a high-intensitylow-volume strength and conditioning training interventiondesigned according to the demands of MMA competition may resultin significant performance improvements for well-trained fighters.
Khatib et al., (2021)	No	Mixed martial arts (MMA) is a sport where the fighters are at high risk of brain trauma, with characteristics, such as the frequency, magnitude, and interval of head impacts influencing the risk of developing short- and long-term negative brain health outcomes. These characteristics may be influenced by weight class as they may have unique fighting styles. The purpose of this research was to compare frequency, mag- nitude, and interval of head impacts between lightweight and heavy- weight fighters in professional MMA. Frequency, interval, event type, velocity, and location of head impacts were documented for 60 fight- ers from 15 Lightweight and 15 Heavyweight professional MMA fights. Head impact reconstructions of these events were performed using physical and finite element modelling methods to determine the strain in the brain tissues. The results found that LW and HW fighters sustained similar head impact frequencies and intervals. The LW fight- ers sustained a significantly higher frequency of very low and high magnitude impacts to the head from punches; HW a larger frequency of high category strains from elbow strikes. These brain trauma profiles reflect different fight strategies and may inform methods to manage and mitigate the long-term effects of repetitive impacts to the head.

Kingery et al., (2021)	Yes	Background: Mixed Martial Arts (MMA) is an increasingly popular combat sport incorporating striking and grappling that results in a high incidence of injuries. Objectives: The purpose of this study was to analyze the impact of injuries on the return to sport and post-injury performance of professional MMA athletes. We hypothesize that increased age is associated with lower probability of return to sport and diminished post-injury performance. Methods: Publicly available data (obtained from ESPN.com/MMA, UFC.com, Rotowire.com/MMA) from professional MMA fighters who resigned from fight cards due to injury from 2012 to 2014 were analyzed. Injury history, match history and outcomes, and duration of time to return to professional fighting were recorded and compared to a cohort consisting of uninjured opponents. Results: 454 fighters were included in the analysis. The mean age at the time of injury was 30.0 ±3.9 years. 94.4% of injured athletes were able to return to professional MMA, and athletes required a mean duration of 6.8±6.7 months between injury and their next professional fight (range 0.3– 58 months). There was no significant difference in winning percentage in the post-injury period between the injured group and the uninjured group (p = 0.691). Increased age at the time of injury was associated with the odds of being able to return to professional fighting after injury (OR = 0.822, p = 0.001). Conclusion: In this analysis of publicly available injury data on MMA fighters, there was a high rate of return to professional sport and no evidence of an associated decline in performance following major injury requiring withdrawal from a fight card. Older age at the time of injury was associated with decreased odds of being able to return to professional fighting. With increasing popularity of combat sports, sport-specific prognostic information will help guide and treat specific injuries associated with MMA participation.

Landers et al., (2017)	No	Aim: The purpose of this study was to determine the amount of visual acuity losswith head movement in actively training mixed martial arts (MMA) fighters. Methods:Vestibulo-ocular reflex function of 22 asymptomatic, male MMA fighters (age = 29.2 ±5.1) was assessed by taking the difference between static visual acuity and the dynamicvisual acuity test, in both yaw and pitch planes. Results: The mean static visual acuitytesting logMAR was -0.173 (standard deviation [SD] = 0.114). Mean dynamic visualacuity test values decreased with head movement to 0.196 logMAR (SD = 0.103) in yaw; $p < 0.001$, and to 0.283 logMAR (SD = 0.133) in pitch; $p < 0.001$. Conclusion: MMAfighters had a decay, beyond normal ranges, in visual acuity during head movement.These decreases may suggest vestibulo-ocular reflex impairment and were unrelatedto self-reported concussion history. These results should be cautiously interpretedsince there was not a control group.
Larocca et al., (2019)	No	Traumatic brain injury (TBI) is a major cause of death and disability worldwide, with mild TBI(mTBI) accounting for 85% of cases. mTBI is also implicated in serious long-term sequelaincluding second impact syndrome and chronic traumatic encephalopathy. mTBI often goes undiagnosed due to delayed symptom onset and limited sensitivity of conventional assessment measures compared with severe TBI. Current efforts seek to identify accurate and reliableon-invasive biomarkers associated with functional measures relevant to long-term outcomes.Herewe evaluated the utility of serum and salivary microRNAs (miRNAs) to serve as sensitiveand specific peripheral biomarkers of possible mTBI. Our primary objectives were to establishthe relationship between peripheral measures of miRNA, objective quantification of headimpacts, and sensitive indices of balance and cognitive function in healthy young adult athletes.A secondary objective was to compare the sensitivity of miRNA versus commonly usedblood-based protein biomarkers. 50 amateur mixed martial arts (MMA) fighters participated. 216saliva and serum samples were collected at multiple time points, both

		pre- and post-fight. Levelsof 10 serum proteins were compared in a subset of the fighters (n = 24). Levels of miRNAswere obtained by next generation sequencing. Functional outcomes were evaluated using a computerized assessment system that measured cognitive performance, body sway, and combinedcognitive performance and body sway during dual task completion. Data were analyzed using multivariate logistic regression for predictive classification, analysis of variance, correlation analysis and principal component analysis. We identified a subset of salivary and serum miRNAs that showed robust utility at predicting TBI likelihood and demonstrated quantitative associations with head impacts as well as cognitive and balance measures. In contrast, serum proteins demonstrated far less utility. We also found that the timing of the responses varies in saliva and serum, which is a critical observation for biomarker studies to consider.
Lindsay et al., (2015)	No	Cold water immersion is thought to reduce the inflammatory response to injury. Using cultured mono- nuclear cells and human subjects in a mixed martial arts (MMA) contest, we examined the effect of cryotherapy on 7,8-dihydroneopterin and neopterin generation. Urine was collected from 10 elite male mixed martial artists before, immediately post and 1, 2, 24 and 48 h following a con- test. Myoglobin was analysed by reverse-phase high per- formance liquid chromatography, and urinary neopterin and total neopterin (neopterin+7,8-dihydroneopterin) were measured by strong cation exchange high-perfor- mance liquid chromatography. Cold water immersion and passive recovery were compared using changes in these markers, while cryotherapy tested total neopterin production in γ-interferon and phorbol myristate ace- tate (PMA)-stimulated blood-derived mononuclear cells (monocytes/T cells). Myoglobin significantly increased ($p < 0.05$) at 1 h post-contest, neopterin significantly increased at 1 and 24 h ($p < 0.05$), total neopterin signifi- cantly increased ($p < 0.05$) at 1 h post for the passive group only, and significant individual

		variation was observed for all markers (p < 0.01). Cold water immersion attenu- ated total neopterin production (p < 0.05), while cryother- apy significantly reduced total neopterin production in PMA-stimulated mononuclear cells (p < 0.01). Cryotherapy attenuates the post-exercise inflammatory response fol- lowing an MMA contest. The evidence also suggests that the mechanisms responsible for this may be related to direct immune cell suppression.
Lindsay et al., (2017)	Yes	Combative sport is one of the most physically intense forms of exercise, yet the effect of recoveryinterventions has been largely unexplored. We investigated the effect of cold-water immersion onstructural, inflammatory and physiological stress biomarkers following a mixed martial arts contestpreparation training session in comparison to passive recovery.Semi-professional MMA competitors (n=15) were randomly assigned to a cold-water immersion (15min at 10ÅãC) or passive recovery protocol (ambient air) completed immediately following a contestpreparation training session. Markers of muscle damage (urinary myoglobin), inflammation/oxidativestress (urinary neopterin + total neopterin (neopterin + 7,8-dihydroneopterin)) and hypothalamicpituitaryaxis activation (saliva cortisol) were determined before, immediately post and one, two and24 hours post-session. Ratings of perceived soreness and fatigue, counter movement jump andgastrointestinal temperature were also measured.Concentrations of all biomarkers increased significantly (p<0.05) post-session. Cold water immersionattenuated increases in urinary neopterin (p<0.05, d = 0.58), total neopterin (p<0.05, d = 0.89) andsaliva cortisol after two hours (p<0.05, d = 0.68) and urinary neopterin again at 24 hours (p<0.01, d =0.57) in comparison to passive recovery. Perceived soreness, fatigue and gastrointestinal temperatureswere also lower for the cold water immersion group at several time points post-session whilst

		countermovement jump did not differ.Combative sport athletes who are subjected to impact-induced stress may benefit from immediatecold water immersion as a simple recovery intervention that reduces delayed onset muscle soreness aswell as macrophage and HPA activation whilst not impairing functional performance.
Little et al., (2015)	No	In antagonistic encounters, the primary decision to be made is to fight or not. Animals may then possess adaptations to assess fighting ability in their opponents. Previous studies suggest that humans can assess strength and fighting ability based on facial appearance. Here we extend these findings to specific contests by examining the perception of male faces from paired winners and losers of individual fights in mixed martial arts sporting competitions. Observers, unfamiliar with the outcome, were presented with image pairs and asked to choose which of the 2 men was more likely to win if they fought while other observers chose between the facesbased on masculinity, strength, aggressiveness, and attractiveness. We found that individuals performed at rates above chance in correctly selecting the winner as more likely to win the fight than the loser. We also found that winners were seen to be more masculine,stronger, and more aggressive than losers. Finally, women saw the winners as more attractive than the losers. Together these findings demonstrate that 1) humans can predict the outcome of specific fighting contests based on facial cues, 2) perceived masculinity and strength are putative cues to fighting success available from faces, and 3) facial cues associated with successful male–male competition are attractive to women.

Marinho et al., (2016)	No	Purpose To describe the morphofunctional characteristicsof elite mixed martial arts athletes. Methods Eight male Brazilian athletes (aged: 31 ± 5 years;training experience: 5 ± 1 years; height: 1.77 ± 0.05 m; bodymass: 82.1 ± 9.6 kg) with national training experience weresubjected to anthropometric evaluation to estimate body compositionand somatotype, andmaximal strength (1RM) in squatand bench press, abdominal and upper limb endurance, andlower limb power were determined. Results Body fat levels of 13.4 ± 5.6 %, lean mass levelsof 69.6 ± 4.6 %, and mesomorphic component (6.4 ± 0.8)were observed. Athletes performed 42 ± 14 sit-ups and37 ± 9 push-ups, and remained for 35 ± 10 s in theflexed-arm hang test. Athletes reached 2.19 ± 0.31 m inthe horizontal jump test, and obtained absolute 1-RMvalues of 80 ± 15 kg and 68.5 ± 6.0 kg and relative valuesof 1.00 ± 0.2 kg/kg and 0.84 ± 0.10 kg/kg in bench press and squat tests, respectively. Conclusion Results indicate body fat levels in accordance with other studies, high lean body mass, and a predominantly mesomorphic component. Abdominal and upperlimb endurance were classified as excellent, while results of the flexed-arm hang test were similar to previous data.Mixed martial arts athletes' lower limb performance in thehorizontal jump was classified as weak. Lower levels ofmaximal strength were obtained in squat and bench press tests.
Matuk et al., (2021)	No	Traumatic brain injury (TBI) is of significant concern in the realm of high impact contact sports, including mixed martial arts (MMA). Extracellular vesicles (EVs) travel between the brain and oral cavity and may be isolated from salivary samples as a noninvasive biomarker of TBI. Salivary EVs may highlight acute neurocognitive or neuropathological changes, which may be particularly useful as a biomarker in high impact sports. Pre and post-fight samples of saliva were isolated from 8 MMA fighters and 7 from controls. Real-time PCR of salivary EVs was done using the TaqMan Human Inflammatory array. Gene expression profiles were compared pre-fight to post-fight as well as pre- fight to controls. Largest signals were noted for fighters sustaining a loss by technical

		knockout (higher impact mechanism of injury) or a full match culminating in referee decision (longer length of fight), while smaller signals were noted for fighters winning by joint or choke submission (lower impact mechanism as well as less time). A correlation was observed between absolute gene information signals and fight related markers of head injury severity. Gene expression was also significantly different in MMA fighters pre-fight compared to controls. Our findings suggest that salivary EVs as a potential biomarker in the acute period following head injury to identify injury severity and can help elucidate pathophysiological processes involved in TBI.
Massey et al., (2013)	No	Objectives: The purpose of the current study was to use a grounded theory methodology to betterunderstand the psychological factors involved in training and competition in MMA.Design: A grounded theory methodology underpinned by an interpretivist epistemology wasutilized inthe current study.Methods: Nine MMA athletes participated in formal, recorded interviews with one athlete participating intwo recorded interviews. Additionally, observations and field notes from the first author were collectedover a one-year period, and aided data collection and analysis. In line with grounded theory methodology(Weed, 2009), an iterative and constant comparison approach to data collection and analysis wasemployed until saturation was reached.Results: Throughout data collection and analysis, self-regulation during a training camp emerged asintegral to optimal performance. Motivation and ongoing evaluation aided fighters in their ability to self-regulateboth external and internal factors related to training and performance. External factors associatedwith self-regulation consisted of the creation and maintenance of an ascetic routine throughenvironmental regulation, social support, and structured amnesty. Internal factors associated with self-regulationconsisted of deliberately induced pain and distress, which facilitated self-efficacy, and produced stress and fatigue.Conclusions: Results of the current study

		advance the scientific literature in sport psychology by examining the role of self-regulation in the performance of MMA fighters. As the popularity of MMA continues to increase, future research should also examine the role of self-regulation in other aspects of MMA (e.g., injury rehabilitation, life balance).
Matthews & Nicholas, (2017)	No	There is a lack of research documenting the weight-making practices of mixed-martial-arts (MMA) competitors. The purpose of the investigation was to quantify the magnitude and identify the methods of rapid weight loss (RWL) and rapid weight gain (RWG) in MMA athletes preparing for competition. Seven athletes (mean ± SD, age 24.6 ± 3.5 yrs, body mass 69.9 ± 5.7 kg, competitive experience 3.1 ± 2.2 yrs) participated in a repeated-measures design. Measures of dietary intake, urinary hydration status, and body mass were recorded in the week preceding competition. Body mass decreased significantly ($p<0.0005$) from baseline by 5.6 ± 1.4 kg (8 ± 1.8%). During the RWG period (32 ± 1 hours) body mass increased significantly ($p<0.001$) by 7.4 ± 2.8 kg (11.7 ± 4.7%), exceeding RWL. Mean energy and carbohydrate intake were 3176 ± 482 kcal.day-1 and 471 ± 124 g.day-1, respectively. At the official weigh-in 57% of athletes were dehydrated (1033 ± 19 mOsmol.kg-1) and the remaining 43% were severely dehydrated (1267 ± 47 mOsmol.kg-1). Athletes reported using harmful dehydration based RWL strategies, including sauna (43%) and training in plastic suits (43%). Results demonstrated RWG greater than RWL, this is a novel finding and may be attributable to the 32 hour duration from weigh-in till competition. The observed magnitude of RWL and strategies used are comparable to those which have previously resulted in fatalities. Rule changes which make RWL impractical should

		be implemented with immediate effect to ensure the health, safety and wellbeing of competitors.
Mayer et al., (2015)	Yes	Growing evidence suggests that temporally proximal acute concussions and repetitive sub-concussive head injuries may lead to long-term neurological deficits. However, the underlying mechanisms of injury and their relative time-scales are not well documented in human injury models. The current study therefore investigated whether biomarkers of brain chemistry (MR spectroscopy: N-acetylaspartate, combined glutamate and glutamine, total creatine**,** choline compounds and myo-inositol) and structure (cortical thickness, white matter/sub-cortical volume) differed between mixed martial artists (MMA; N=13) and matched healthy controls without a history of contact sport participation (HC; N=14). A subset of participants (MMA=9; HC=10) returned for follow-up visits, with MMA (N=3) with clinician-documented acute concussions also scanned serially. As expected, MMA self-reported a higher incidence of previous concussions and significantly more cognitive symptoms during prior concussion recovery. Fighters also exhibited reduced memory and processing speed relative to controls on neuropsychological testing coupled with cortical thinning in the left posterior cingulate gyrus and right occipital cortex at baseline assessment. Over a 1 year follow-up period, MMA experienced a significant decrease in both white matter volume and N-acetylaspartate concentration, as well as relative thinning in the left middle and superior frontal gyri. These longitudinal changes did not correlate with self-

		reported metrics of injury (i.e., fight diary). In contrast, HC did not exhibit significant longitudinal changes over a 4 month follow-up period ($p > 0.05$). Collectively, current results provide preliminary evidence of progressive changes in brain chemistry and structure over a relatively short time period in individuals with high exposure to repetitive head hits. These findings require replication in independent samples.
Mayer et al., (2017)	Yes	Although diffusion magnetic resonance imaging (dMRI) has been widely used to characterize the effects of repetitive mild traumatic brain injury (rmTBI), to date no studies have investigated how novel geometric models of microstructure relate to more typical diffusion tensor imaging (DTI) sequences. Moreover, few studies have evaluated the sensitivity of different registration pipelines (non-linear, linear and tract-based spatial statistics) for detecting dMRI abnormalities in clinical populations. Results from single-subject analyses in healthy controls (HC) indicated a strong negative relationship between fractional anisotropy (FA) and orientation dispersion index (ODI) in both white and gray matter. Equally important, only moderate relationships existed between all other estimates of free/intracellular water volume fractions and more traditional DTI metrics (FA, mean, axial and radial diffusivity). These findings suggest that geometric measures provide differential information about the cellular microstructure relative to traditional DTI measures. Results also suggest greater sensitivity for non-linear registration pipelines that maximize the anatomical information available in T1-weighted images. Clinically, rmTBI resulted in a pattern of decreased FA and increased ODI, largely overlapping in space, in conjunction with increased intracellular and free water fractions, highlighting the potential role of edema following repeated head trauma. In summary, current results suggest that geometric models of diffusion

		can provide relatively unique information regarding potential mechanisms of pathology that contribute to long-term neurological damage.
Mcclain et al., (2014)	No	Objective: To provide an updated comprehensive profile of mixedmartial arts (MMAs) injuries.Design: Correlational and multivariate analyses were conducted oncross-sectional data to examine injuries sustained during 711 MMAbouts. One physician diagnosed any injuries occurring during thebouts. Setting: Various sports venues in Kansas and Missouri holdingMMA competitions. Participants: Male and female and amateur and professionalMMA competitors contributing to 1422 fight participations (fightparticipations = 711 bouts · 2 fighters/bout). Independent Variables: State, level (amateur or professional),gender, number of rounds, and bout outcome (knockout/technical knockout [KO/TKO] vs other outcomes [eg, decision]). Main Outcome Measures: Injuries/fight participations, injury sustained (yes vs no), and fighter referred to emergency room (ER;yes vs no). Results: The overall injury rate was 8.5% of fight participations(121 injuries/1422 fight participations) or 5.6% of rounds (121/2178rounds). Injury rates were similar between men and women, buta greater percentage of the injuries caused an altered mental state inmen. The risk of being injured was significantly greater for bouts held in Kansas, at the professional level, lasting more rounds, and ending in a KO/TKO. Fighters also were more likely to be referred tothe ER if they participated in longer bouts ending in a KO/TKO. Conclusions: The observed injury rate was lower than previouslyreported suggesting recent regulatory changes have made MMAa safer sport. Increased

		clinical awareness and additional research'shold be extended to head-related injuries in MMAs especially those associated with KOs/TKOs.
Miarka et al., (2015)	No	This study aimed to compare time-motion analysis between weight divisions in male fighters and rounds of Mixed Martial Arts (MMA). Thesample consisted of 2097 bouts separated by weight divisions (Flyweight=114, Bantamweight n=224, Featherweight n=286, Lightweight n=450,Welterweight n=390, Middleweight n=316, Light heavyweight n=167,Heavyweight n=150) of 2012-2014 UFC™ events. The time-motion variableswere categorized into low or high-intensity, stand-up or groundwork situations by round. Effects of weight divisions were observed and themain results showed that, in the 1st round, the heavyweights(212.4±101.5s) presented with a shorter effort time than all other weight divisions (257.6±79.9s); in the 2nd round, the bantamweight competitors(132.8±90.9s) presented with the shortest total effort time (171.7±81.5s);in the 3rd round, the shortest total effort time was in the heavyweight division(246.3±89.1s) again, with the other weight divisions showing variableeffort times, including: welterweight (289.6±42.3s), lightweight(280.3±57.3s), featherweight (281.4±58s), bantamweight (285.6±47.2s),flyweight (287.7±43.8s) and middleweight (268.3±72.2s). The present datasuggest a focus on the intermittent demands presented in the various combatphases in MMA. Additionally, strength and conditioning coaches should pay particular attention to the high and low effort ratios for bothstanding and ground combat to most effectively condition their athletes.

Miarka et al., (2016)[a]	Yes	The aim of this study was to compare time-motion and technical-tactical analysis between pairedoutcomes and rounds of mixed martial arts (MMA) matches.The sample consisted of 645 rounds of MMA competitionpaired by outcomes (first round, winners n = 215 and losersn = 215; second round, winners n = 215 and losers n =215; third round, winners n = 215 and losers n = 215). Thetime-motion variables were categorized into low-intensity orhigh-intensity, stand-up or groundwork situations. Stand-up techniques were analyzed by observing total strikes to thehead and body, and takedowns. The actions on the groundwere analyzed by observing submission activity, includingsuccessful choking and joint locking actions, and also positional improvements, including advances to the mount, half guard, and side and back positions. Chi-squared and Wilcoxontests were conducted with a significance level of p #0.05. Results showed that winners had higher values for total strikes and submissions in all rounds, and also positionalimprovements, over losers. The standing combat withlow-intensity comparisons presented differences betweenthe rounds first, with a median of 2:33.5 (P25–P75%:1:20–3:56) minute, second, with 2:37 (1:24–3:59) minute,and third, with 2:07 (1:06–3:39.2) minute. These data suggest focus on the intermittent demand presented in combatphases with a special attention to the strike and groundtechnical-tactical skills; strength and conditioning coaches could emphasize the effort pause ratios for both standingand ground combat that mimic the requirements of MMA,especially during the third round.

Miarka et al., (2016)[b]	Yes	The aim of the present study was to compare the technical-tactical aspectsof female professional mixed martial arts (MMA) matches by combat outcomesand rounds from the Ultimate Fighting Championship (UFC™).We analyzed 174 rounds separated by Combat Outcomes (Split Decision=54; Unanimous Decision n=72; KO/TKO n=28; Submission n=20) of2012-2014 events. The time-motion variables were categorized into totalcombat time separated by the amount of time spent performing low orhigh intensity activity per round, on stand-up or groundwork situations. The principal findings showed significant differences between Split andUnanimous Decision outcomes vs. KO/TKO and Submission groups instand-up combat with low intensity by round (160.4±83.6s and158.4±87.6s vs. 44.8±38.8s and 42.1±44.1s, respectively, p<0.001) and intotal combat time by round (300.7±0.3s and 300.0±0.4s vs. 154.4±95.2sand 204.2±96.6s, respectively, p<0.001). Significant differences werefound when compared the Total, Head and Leg Strikes Attempts, wherewinners by KO/TKO and Submission demonstrated lower frequenciesthan Split and Unanimous Decision(p<0.001; for all comparisons).. Inconclusion, technical-tactical skills can be associated with contextualizedpractices, where female athletes who finalized the matches by KO/TKOand Submission had higher values of striking and grappling actions duringthe groundwork combat, while those who had Split or Unanimous Decisionoutcomes showed higher values of striking actions during stand-up combat.
Miarka et al., (2016)[c]	Yes	The aim of this study was to compare time-motion and technical analysisin mixed martial arts (MMA) home advantage situation with paired boutsof the same athletes in home and away matches. For this purpose, weanalyzed, 202 bouts and 606 rounds from UFC™ events (2012-2014),paired by both athletes competing at home (n=101) or away (n=101). Thetime-motion variables were categorized into low or high intensity, standup or groundwork situations. Techniques were analyzed by observing totalstrikes to the head and body, takedowns, submission,

		choking and jointlocking actions. Repeated measures analysis of variance with Bonferroni was used to compare the situations, p≤0.05. A significant effect wasobserved when comparing home vs. away bouts in total strikes landed(21.4±16.2 vs. 27.7±21.4 attempts), total strikes attempt (37.3±23.9 vs.46.4±26.3 attempts), head strikes attempts (21.5±15.1 vs. 26.5±16.2attempts) and in body strikes attempts (4.3±3.6 vs. 3.4±3.8 attempts). Timemotionand technical-tactical differences did not impact the combats 'outcomes suggesting tactical adjustments by professional athletes fromMMA, according to the condition of fighting.
Miarka et al., (2017)[a]	No	This study compared motor actions and spatiotemporal changes between weight divisions fromUltimate Fighting Championship (UFC™), conducting a practical application for mixed martialarts (MMA) training. For this, we analyzed 2814 rounds of all weight divisions by motor actionsand spatiotemporal changes according a ctions and time of the Keeping distance, Clinch andGroundwork combat phases. We observed differences between weight divisions in the keepingdistance on stand-up combat (p ≤0.001; with lower time in Featherweight 131.4 s and bantamweight127.9 s) clinch without attack (p≤ 0.001; with higher timer in Flyweight 11.4 s andHalf-middleweight 12.6 s) and groundwork without attack (p ≤0.001; with higher timer in Halfmiddleweight0.9 s). During keeping distance, half-middleweight presented a higher frequency ofHead Strikes Landed (p =0.026; 7 ± 8 times) and attempted (p =0.003; 24 ± 22 times). Inclinch actions heavyweight present a higher frequency (p ≤0.023) of head strike landed (3 ± 7times) and attempted (4 ± 9 times) and half-middleweight for body strikes (p ≤0.023) landed(2 ± 5 times) and attempted (3 ± 5 times). At the last, during groundwork, Bantamweightpresent a higher frequency (p ≤0.036) of head strikes landed (8 ± 10 times) and attempted(10 ± 13 times) body strikes landed (p ≤0.044; 3 ± 5 times) and attempted (3 ± 6 times).This study reveals important point to training and provide a challenge applied referential to theconditioning plains. From the weight divisions differences should be

		aware of the increase in thefrequency of distance actions, especially in light and middleweights. On the Ground, bantamweightcan focus on striking and grappling actions than others.
Miarka et al., (2017)[b]	Yes	The purpose of this study was to realise an objectivity performanceanalysis based on technical–tactical actions in different MMAspatiotemporal phases (i.e. keeping distance, clinch and groundcombat), and to estimate the extent to which technical–tactical actionsin different combat phases could increase the probability of winning.The sample was composed of 1,752 rounds of 584 UFC bouts (Winningvs. Losing) from 67 events in 2013 and 2014. Technical–tactical actions during spatiotemporal changes (i.e. keeping distance, clinchand ground) were observed, according to frequency of attemptedand landed strikes (separated in leg, body and head orientations),takedowns, chokes, locks and submissions. Wilcoxon was applied tocompare outcomes, and a logistic regression analysis was used toconfirm the effects of technical–tactical actions on the dichotomousperformance (losing vs. winning), $p \leq .05$. The main results showedsignificant differences in all spatiotemporal changes, except for headand leg strike attempts while keeping the distance. The performanceprobability was 69.6% for outcomes, 64.5% for winning vs. 74.7% forlosing. Furthermore, when verified the Wald's criterion, it is possibleto corroborate that Head Strikes Landed Keeping Distance, OffensivePasses and Takedowns Landed were the most significant variables,which contributed to probability of predicting winning bouts.

Miarka et al., (2018)[a]	No	This study aimed to support training program development through thecomparison of performance analysis of professionals mixed martial arts (MMA) athletesin the bouts that were not finished by points. Using digital recordings of each bout, weanalyzed 1,564 rounds (678 bouts) which were separated by Ending and other Rounds.Our results indicated that KO/TKO is the main outcome that defines the ending round(≈60%), however, there is a higher frequency of ending by submission on the 1st and 2[nd]round (>30%). Bouts ending during the 1st or 2nd rounds had shorter total time andstanding combat with low-intensity than ending in the 3rd round (91.5±71.4, 93.4±67.5and 143.2±87.4; for low-intensity in the 1st, 2nd and 3rd round respectively; $p<0.05$),while standing combat time with high intensity was longer in the last round incomparison to bouts that finished in the 1st or 2nd rounds (7.4±9.2, 9.7±18.0 and17.7±29.1 for high intensity in the 1st, 2nd and 3rd round respectively; $p<0.05$). Thelower time dedicated to low intensity stand up combat actions, regardless of round, andforcefulness of the actions in groundwork in the 1st and 2nd rounds appear to be elementsthat increase the probability of success in professional MMA bouts, these factors haveessential implications related to training program design.
Miarka et al., (2018)[b]	Yes	This study compared the technical–tactical analysis by time ratios between genders and weight divisions of female mixed martial arts(MMA) athletes competing in the Ultimate Fighting Championships.Eighty-two athletes (G1 = 21 male middleweights, G2 = 14 malelightweights, G3 = 21 female middleweights, G4 = 14 female lightweights), were evaluated using a time–motion and technical–tactical protocol. Four hundred eighty-four males and 205 female MMArounds were analyzed. Time–motion and technical–tactical variables were evaluated according to frequency of attempts and effective strikes, takedowns and submissions, as well as fighting activity time and preparatory of standing and groundwork combat. Comparing groundwork preparatory activity time G1 showed longer times than

		other groups (35:0 ± 1.8 s; p ≤ .002). For standing fighting activity time,G2 showed longer times than other groups (151.2 ± 72.9 s; p ≤ .001).G2 displayed longer times between actions during standing [p = .003;1: 32.5 (17.8; 74.8)] and groundwork [p ≤ .003; 1: 30.0 (9.0; 52.0)] thanG3. In conclusion, G1 and G2 showed longer groundwork and standing actions than G3 and G4. In standing and groundwork actions, G2presented longer time between striking actions than G3.
Miarka et al., (2019)[a]	No	This study aimed to compare time-motion and technical-tactical behaviours of professional mixed martial arts (MMA) bouts with afocus on longer bouts that were finished during 3rd round vs. 104th round vs. 5th round, comparing rounds. We analysed 779rounds (264 bouts – 3rd round n = 120; 4th round n = 92; ©and;5th round n = 52) which were compared by rounds. Our mainresults showed that bouts with 5th showed higher percentage/round of lowintensity time than the 3rd round (79% vs. 75%) andthe 4th rounds demonstrated higher percentage/round of lowintensity time effort 20 than other rounds (84% vs. 76%).Regarding striking actions, 1st round (39.5 ± 17.6) and 4th round(19.8 ± 12.5) showed lower frequency of strikes attempted than 2ndround (46.1 ± 21.9) and 3rd round (46.1 ± 27.0). This study demonstrated new parameters for long MMA bouts with 3, 4 and 5 ending rounds and other rounds.
Miarka et al., (2019)[b]	Yes	This research sought to determine actions during bouts which generate serious enough injury to stop the bout, verifying the injury incidence, types, and prevalence of doctor stoppages (doc-stoppage), and identify potential risk factors by analyzing technical-tactical profiles for injury in sanctioned mixed martial arts (MMA) bouts taking place over a 12-year period. *Methods*: The research analyzed 440 paired MMA matches separated by doc-stoppage (n=220) and no doc-stoppage (n=220) from 2002-2014. Technical-knockouts for doc-stoppage were diagnosed and managed by attending ringside doctors and the time-motion variables were categorized into total combat time separated by low- or high-intensity activity per round, stand-up or groundwork actions,

		*p_≤0_._0_5_*. *Results:* The main cause of injuries in doc-stoppage situations were due to facial injuries (>90%), with 87.1% occurring after striking actions during the second round. Lacerations were the leading type of injury, which occurred with 80% frequency. The results showed differences between doc-stoppage and no doc-stoppage for standing combat with low-intensity actions (130.6±8.5s vs. 83.3±6.9s for first round; 115.7±10.5s vs. 100.1±9.6s for second round and 121.5±19.5s vs. 106.3±11.7s for third round) and total strike attempts (34.5, 23.0-51.8 vs. 25.0, 12.0-40.8); in standing combat, head strike attempts (21, 10-33 vs. 11, 4-21) and body strikes (2.5, 1.0-5.8 vs. 1.0-2); and in groundwork combat, head strikes landed (0.0-3.0 vs. 0.0-5.0). *Conclusions*: Our research showed higher values of strike attempts with two main orientations, namely the head (on the ground and in stand-up actions) and body (in stand-up actions), and may provide important information regarding the technical knockout (TKO) and when it can be called by officials supervising MMA bouts.
Miarka et al., (2021)	No	The current analysis is essential to recognize essential mechanisms that are important and their intensity. The present study thus aimed to (i) typify the performance effects during continuous self-paced sequential female MMA rounds, (ii) verify the round effects in time-motion results of female MMA athletes during sequential combat phases, and (iii) verify the technical- tactical behaviors attempted and landed during each ending- round and round. The sample was composed of 13,572 sequential behavior analysis from 74 different female MMA fights that ended in KO or TKO. It included 174 rounds, separated into the round (1Rx1ER) of combat ending in the first round (*n* = 22); first round (1Rx2ER) and second round (2Rx2ER) of combat ending in the second round (*n* = 28); and first round (1Rx3ER), the second round (2Rx3ER), and third round (3Rx3ER) of combat ending in the third round (*n* = 124) from matches between 2014 and 2018 from all weight divisions from the following events: UFC 220, UFC 221, UFC Fight Night 124, UFC on Fox 28, UFC 228, UFC 223, UFC

		on FOX 29, UFC 225, UFC 224, UFC 226, UFC Fight Night 126, UFC on FOX 30, UFC Fight Night 125, Bellator 221, UFC Fight Night 129, ONE FC, Invicta FC 30, Invicta FC 28, Invicta FC 27, and Invicta FC 32. The main results indicated an increase in the frequency of attacks to the head, body, leg orientations, and the preparatory activity time compared to 1ER vs. 3ER. The correlation of round phases during MMA combats and critical technical-tactical behaviors, such as takedowns, submissions, and strike attempts in different orientations (i.e., to the head, body, or leg), represent the main aspects of attacking systems used by women. Besides, to obtain information for conditioning and strength training of female athletes, it is significant to highlight that open task and intermittent combat sports involve complex sequential skills, with a range of around 92.2 ± 71.0–162.3 ± 89.4s of low-intensity behaviors to 33.5 ± 52.2–69.8 ± 79.6s by high intensity.
Murugappan et al., (2021)	Yes	Rapid weight loss or "weight cutting" is a common but potentially harmful practice used in mixed martial arts competition. Following the official weigh-in, competitors refeed and rehydrate themselves in a process known as rapid weight gain (RWG) to realize a potential competitive advantage. While data from surveys and small series have indicated the majority of mixed martial arts athletes engage in rapid weight loss, there is a lack of officially collected data from sanctioning organizations describing its prevalence. The present investigation represents a summary of the data collected between December 2015 and January 2018 by the California State Athletic Commission. In total, 512 professional mixed martial artists (455 males and 57 females) were included. Of these, 503 (98%) athletes gained body mass between weigh-in and their bouts. Total RWG between weigh-in and competition was 5.5 ± 2.5 kg, corresponding to an 8.1% ± 3.6% body mass increase. Total RWG was 5.6 ± 2.5 kg (8.1% ± 3.6%) for males and 4.5 ± 2.3 kg (8.0% ± 3.8%) for females. More than one quarter of men and one third of women gained >10% body mass between weigh-in and competition. Athletes from

		leading international promotions gained more absolute, but not relative, body mass than those from regional promotions. Our findings indicate RWG is nearly ubiquitous in professional , with a similar prevalence in male and female athletes. Trends based on promotion suggest a larger magnitude of RWG in presumably more experienced and/or successful mixed martial artists from leading international promotions.
Ngai et al., (2008)	No	Background: Professional mixed martial arts (MMA) competition is a full-contact sport that has risen rapidly inpopularity in recent years. However, there is limitedinformation regarding the incidence of competition injuriesafter sanctioning by an athletic commission.Methods: We conducted a retrospective cohort study toexamine MMA injury patterns during a 5 year period after sanctioning in the state of Nevada. Data from all regulatedMMA competitions during the study period from March2002 to September 2007 (1270 fight exposures) was obtained. Injury odds ratios were calculated by conditionallogistic regression on match outcome, age, weight, andfight experience, using a pair-matched case-controldesign (n=464) and by multiple logistic regression onmatch outcome, age, fight experience, weight, combatminutes, and scheduled rounds.Results: During the 635 professional MMA matches, 300of the 1270 athletes sustained documented injuries withan injury rate of 23.6 per 100 fight participations. Mostcommon reported injuries were lacerations and upper limbinjuries. Severe concussion rate was 15.4 per 1000athlete exposures, or 3% of all matches. No deaths orcritical sports-related injuries resulted from any of theregulated matches during the study period. Age, weightand fight experience did not statistically increase thelikelihood of injuries after controlling for other covariates.Conclusions: Injury rates in regulated professional MMAcompetition are similar to other combat sports; the overallrisk of

		critical sports-related injury seems to be low.Additional study is warranted to achieve a better understanding of injury trends and ways to further lower Injury risk in MMA.
Niewczas et al., (2021)	No	Four factors—namely, harm avoidance, novelty seeking, reward addiction and persistence— represent the nature of temperament that is not genetically determined in itself. It was shown in earlier studies that a strong propensity to look for novelty or a tendency to engage in risky behavior is correlated with genetic variants in the area of the genes encoding dopamine receptors. Therefore, the aim of this study is to determine whether there is a relationship between personality traits and genetic variants in the area of the *DRD2* dopamine receptor gene in MMA athletes. The participants consisted of 85 mixed martial arts (MMA) athletes and 284 healthy, non-MMA male participants. Their personality traits were measured using the Revised Temperament and Character Inventory. Blood was collected for genetic assays and all samples were genotyped using the real-time PCR method. We observed a statistically significant effect of a complex factor of the *DRD2 rs1799732* genotype on MMA participants' control and reward dependence. Engaging in high-risk sport may be associated with several personality characteristics. The *DRD2 rs1799732* polymorphism may be associated with reduced harm avoidance in martial arts athletes, thereby modulating athletes' predisposition to participate in high-risk sport.

Park et al., (2019)	No	Background: Similar to other combat sports, mixed martial arts (MMA) includes divisional weight classes. Thepurpose of our research was to further investigate the amount of weight professional MMA fighters lost prior to weighing in for competition, their methods used to cut weight, and their sources of advice on how to cut weight. Methods: This survey was administered to 92 male professional MMA athletes. The survey questions includedduration of overall weight loss prior to competition, methods of weight-cutting, and their sources of adviceregarding weight cutting.Results: When comparing the number of methods of weight cutting with the source of advice, those whoreceived their advice from social media used slightly more methods of weight cutting ($M = 4.86$, $SD = 1.27$) thanthose who did not ($M = 4.02$, $SD = 1.55$); $t(90) = -2.53$, $p < .05$. MMA athletes that used the help of a registereddietitian nutritionist also reported using the least amount of methods for weight-cutting thanany other category($M = 3.84$, $SD = 1.67$). Those that used teammates and did not use a registered dietitian nutritionist used slightlymore methods ($M = 4.46$, $SD = 1.41$) than those who used a registered dietitian nutritionist.Conclusions: The findings of this study report that professional MMA athletes do undergo rapid weight lossthrough various methods to make weight for competition. This study adds evidence to the literature that mostprofessional MMA athletes undergo RWL for competition without the guidance of a registered dietitian nutritionist.It is unclear what the effect of using a registered dietitian nutritionist may have on an MMA athletes' ability toreduce weight in a safe and effective manner. Future research should seek to investigate if employing a registereddietitian nutritionist may lead to a higher rate of success for MMA athletes to make weight, and help reduce adverse risks of RWL.

Pavelka et al., (2020)	No	Mixed Martial Arts (MMA) is a multielement combat sport where fighters need to quickly react to an opponent's movements under fatigued conditions. Research indicates that fast reaction time is important in many sports, but the effect of fatigue has shown negative, null, or even positive influences on reaction time. However, few studies have been conducted in a controlled setting, especially using MMA fighters, whose matches are frequently resolved in a split-second. Therefore, this study investigated whether acute neuromuscular fatigue affects reaction and movement times, and their consistency in MMA fighters (N = 45).Before and after an upper-body Wingate test, a simple visual reaction time task was completed. Results showed a significant negative effect of fatigue on the reaction times and their consistency, with longer reactions (1.5% change) and lower consistency (14.7% change)after the Wingate test. Further, greater amounts of fatigue during the Wingate test seemed to negatively affect the consistency of post-Wingate movement time. Due to cumulative fatigue and the dynamic nature of MMA, our data indicate that not only the decrements in aerobic and anaerobic power likely affect a fighter's performance, but their reaction time and motor time may also be compromised during a fight.
Peacock et al., (2018)[a]	No	The purpose of this investigation is to present observational data regarding sleep variablesin professional Mixed Martial Arts (MMA) athletes. These sleep performance measures were relatedto physical performance and injury in MMA athletes. Eight professional athletes were placed intoa quasi-controlled, multivariable fight-camp environment for a six-week period in preparation forfight competition. Throughout a six-week fight camp environment, athletes were continuously monitored for sleep performance measures (sleep latency, sleep efficiency, onset, and wake variances)via validated wearable sleep monitoring technology. Athletes were tested seven days prior tocompetition on measures of physical performance (vertical jump, VO2max, heart rate recovery,prowler sled push, and pull-ups). Multiple

		correlational analyses were utilized to assess relationshipsbetween all sleep and physical performance measures. There were significant (P < 0.05) correlationsbetween sleep latency and VO2max, heart rate recovery, prowler sled push, vertical jump, and missedpractice sessions. There were also significant (P < 0.05) correlations between average fall asleep timeand heart rate recovery. Lastly, there were significant (P < 0.05) correlations between sleep efficiency,heart rate recovery, and missed practice sessions. MMA athletes who exhibited consistency in sleepdemonstrated stronger relationships with performance testing during the fight-camp period.
Peacock et al., (2018)[b]	No	With the recent increase inpopularity of Mixed Martial Arts (MMA), it is imperative to assess the effectiveness of strength and conditioning (S&C) modalities whileencouraging other S&C professionals to do so as well. There isminimal literature evaluating the role of S&C, and even less literature evaluating the role of the traditional mixed martial art, Tai Chi (TC)as it applies to MMA. Twelve professional MMA athletes were tested on balance, flexibility, and power following a 6-wk S&C program thatincluded TC in the form of a cool-down. The data suggestedimprovements (P≤0.05) in both balance (Balance Error ScoringSystem, BESS) and flexibility (sit and reach) following the 6-wk blockof MMA S&C in adjunct with TC.
Petrou et al., (2021)	Yes	The present study examined, for the first time, the multivariate association between social norms, negative self-conscious emotions, and self-regulatory efficacy and doping intentions in an international sample of MMA athletes, with an emphasis on moderation and mediation effects. We also examined whether MMA athletes with different doping experiences also differed in doping-related self-conscious emotions, self-regulatory efficacy, social norms and doping intentions. A cross-sectional survey- based design was used, and structured anonymous online questionnaires were completed by 249 MMA athletes from 16 countries. Three groups of users were identified based on self- reported doping use:

		never users, never user contemplators, and ever users. One-way ANOVA showed that athletes with differed doping experiences gave significantly different scores in social norms, self-conscious emotions, self-regulatory efficacy, and doping intentions. Hierarchical regression analysis showed that doping intentions were significantly associated with perceiving greater social approval of doping among referent others (injunctive norms), anticipating less negative self-conscious emotions from doping, and with lower levels of self-regulatory efficacy, after controlling for the effect of past doping use. Moderated regression analysis showed that self-conscious emotions did not interact with social norms in predicting doping intentions. Regression-based mediation analysis further showed that self-regulatory efficacy significantly mediated the association of injunctive norms and self-conscious emotions with doping intentions. Our findings highlight the role of social norms and self-conscious emotions in the decision-making process underlying doping in MMA athletes. The practical implications of our findings are discussed within the context of clean sport education and related campaigns to prevent doping in MMA.
Plush et al., (2021)	No	Mixed martial arts (MMA) is a combat sport that employs techniques from different combat disciplines. There are a multitude of technical and physiological characteristics that contribute to competitive success. Developing a single scientific assessment that can predict competitive outcomes poses great difficulty due to the complexity of MMA. While previous research has investigated some important physiological characteristics, there is no accepted best-practice for a comprehensive testing battery. As such, this study aimed to design and utilize a battery of physiological assessments to cover aerobic and anaerobic function, strength measures including explosive and maximal strength, body composition and repeat effort ability in Australian MMA athletes. Six participants with competitive experience were recruited. Testing involved a familiarization, three experimental sessions and including

		assessments such as the isometric midthigh pull, Wingate test, graded exercise test, countermovement jump and body composition scan. Results showed the testing battery in this study was realistic and able to be completed by the participants without issue and that regional Australian MMA athletes were similar physiologically to elite standard compared with previous research taken from a range of sources. However, future research with the testing battery is required with larger and more diverse samples to better understand the full profiles of MMA athletes. The results of the study can help inform athletes, researchers and support staff alike when deciding upon which testing protocols to use for MMA athletes. Future research should aim to develop normative data using the battery proposed in the current study.
Rahmani et al., (2017)	No	Background: Mixed martial arts is a sport that has recently enjoyed a significant increase in popularity. This rise inpopularity has catapulted many of these ''cage fighters'' into stardom and many regularly use social media to reach out totheir fans. An interesting result of this interaction on social media is that athletes are sharing images of their radiologicalexaminations when they sustain an injury. Purpose: To review instances where mixed martial arts fighters shared images of their radiological examinations onsocial media and in what context they were shared. Material and Methods: An Internet search was performed using the Google search engine. Search terms included''MMA,'' ''mixed martial arts,'' ''injury,'' ''scan,'' ''X-ray,'' ''fracture,'' and ''break.'' Articles which discussed injuries to MMAfighters were examined and those in which the fighter themselves shared a radiological image of their injury on socialmedia were identified.Results: During our search, we identified 20 MMA fighters that had shared radiological images of their injuries on socialmedia. There were 15 different types of injury, with a fracture of the mid-shaft of the ulna being the most common. Themost popular social media platform was Twitter. The most common imaging modality X-ray (71%). The majority ofinjuries were sustained during competition (81%)

		and 35% of these fights resulted in a win for the fighter. Conclusion: Professional mixed martial artists are sharing radiological images of their injuries on social media. This maybe in an attempt to connect with fans and raise their profile among other fighters.
Rainey (2009)	No	Background. Mixed martial arts (MMA) is currently thefastest growing sport in the United States and has recently surpassed boxing as the most popular full contact sport.Due to the physical nature of the sport, MMA is associated with various types of injuries.Objective. The purpose of this study was aimed at identifying prevalence and assessing the severity, location, andtype of injuries in MMA athletes sustained during MMArelated activities in the twelve month period prior to thesurvey.Methods. A total of fifty-five subjects between the ages of18 to 39 participated in the study. Participants were givena two-part questionnaire to collect demographic andinjury data.Results. Two hundred seven injuries were reported in thestudy. Low belt ranks had significantly more injuries morethan any other belt rank, resulting in more than two timeshigher injury rate. Professional fighters had significantly more injuries than amateur fighters, resulting in threetimes higher injury rate. The most common body regioninjured was the head/neck/face (38.2%), followed by thelower extremities (30.4%), upper extremities (22.7%),torso (8.2%), and groin (0.5%). Injuries to the nose (6.3%),shoulder (6.3%), and toe (6.3%) were the most common.The most common type of injury was contusions (29.4%),followed by strains (16.2%), sprains (14.9%), and abrasions(10.1%).Conclusion. Injury prevention efforts should consider theprevalence and distribution of injuries and focus on reducingor preventing injuries to the head/neck/face

		in MMArelated activities. Preventative measures should focus onimproving protective equipment during training, and possiblecompetition rule modifications to further minimize participant injury.
Ruiz-Barquín et al., (2019)	No	The aim of this study was to describe the perception of sports talent and excellence development in a sample of MMA fighters, and compare the obtained results with those of previous studies. A total of 42 adult (male and female) MMA athletes of several levels (amateur, semi-professional and professional) participated in the study. A socio-demographic questionnaire and the Psychological Characteristics of Developing Excellence Questionnaire – PCDEQ were used for collecting data. Statistical analyses included descriptive statistics and student’s *t*-test for means comparison. In general, MMA athletes obtained higher scores than those reported by samples of other sports in previous studies, and more specifically in factors I - Support for long-term success, II - Imagery use during practice and competition, and IV - Ability to organise and engage in quality practice. This can be explained due to the higher average age of our sample and the professional or semi-professional level already achieved by many of the MMA fighters. The study also revealed that MMA fighters may need specific psychological training related to factor III - Coping with performance and developmental pressures.

Richardson et al., (2021)	No	Growing evidence suggests that human males have been sexually selected for violent contest competition. I propose the hypothesis that increased arm length is an intrasexu- ally selected adaptation for fighting in males. Longer arms may have provided several advantages to our male ancestors during conflict. However previous research on the effects of arm span on fighting success have shown mixed results and may not have fully accounted for allometric scaling of arm span with size. In a sample of 1,660 modern mixed martial arts fighters, I find that arm span is sexually dimorphic and associated with fighting success, even when controlling for body size. However, effects of arm span on fighting success were very small, suggesting that selection may have been weak. I review evidence for alternative explanations for men's longer arm span and propose future directions to further test this hypothesis.
Santos-Junior et al., (2020)	No	Purpose The aim of this study was to investigate the weight loss behaviors among Brazilian professional mixed martial arts(MMA) athletes. Methods One hundred and seventy-nine Brazilian professional MMA athletes (164 males and 15 females; age: median25 years, range = 19–37 years) from different regions of the country answered an adapted version of a questionnaire developedto characterize weight loss behavior. In addition, questionnaire scores were associated with extremes in behavior. Results All athletes stated that they had engaged in weight loss procedures to make weight for competition and ~ 35% had engaged in weight loss procedures twice in the last season, usually losing a magnitude of up to 10% of their body mass.About 26% started their weight loss procedures in a time span of 30 days prior to competition. When athletes were analyzedby sex and weight classes, no significant differences were found in the questionnaire score. In contrast, international levelathletes showed significant differences in the questionnaire score when compared to state ($P < 0.001$) and national ($P < 0.05$)level athletes. Athletes also reported always use a combination of gradual diet (64.2%), restricting fluid intake (62.6%), andsweat suits

		(55.9%) as methods to cut weight. Additionally, athletes reported the usage of diuretics (~ 49%) and laxatives(~ 32%) at least once in their professional MMA carrier. The most influential sources of weight management behaviors werecoaches, training partners, and physical trainers. Conclusion Brazilian professional MMA athletes commonly undergo weight loss procedures through harmful and illegalmethods regardless of sex, weight class, although international level athletes demonstrated weight management behaviorthat was found to be even more aggressive.
Shin et al., (2014)	No	BACKGROUND AND PURPOSE: Traumatic brain injury is common in fighting athletes such as boxers, given the frequency of blows to thehead. Because DTI is sensitive to microstructural changes in white matter, this technique is often used to investigate white matter integrityin patients with traumatic brain injury. We hypothesized that previous fight exposure would predictDTI abnormalities in fighting athletesafter controlling for individual variation.MATERIALS AND METHODS: A total of 74 boxers and 81 mixed martial arts fighters were included in the analysis and scanned by use ofDTI. Individual information and data on fight exposures, including number of fights and knockouts, were collected. A multiple hierarchical linear regression model was used in region-of-interest analysis to test the hypothesis that fight-related exposure could predict DTI values separately in boxers and mixed martial arts fighters. Age, weight, and years of education were controlled to ensure that these factors wouldnot account for the hypothesized effects.RESULTS: We found that the number of knockouts among boxers predicted increased longitudinal diffusivity and transversal diffusivity in white matter and subcortical gray matter regions, including corpus callosum, isthmus cingulate, pericalcarine, precuneus, and amygdala, leading to increased mean diffusivity and decreased fractional anisotropy in the corresponding regions. The mixed martial arts fighters had increased transversal diffusivity in the posterior cingulate. The number

		of fights did not predict any DTI measures in either group. CONCLUSIONS: These findings suggest that the history of fight exposure in a fighter population can be used to predict microstructural brain damage.
Sinnett, Maglinti & Kingstone., (2018)	No	Background :Grunting is pervasive in many athletic contests, and empirical evidence suggests that it may result in one exerting more physical force. It may also distract one's opponent. That grunts can distract was supported by a study showing that it led to an opponent being slower and more error prone when viewing tennis shots. An alternative explanation was that grunting masks the sound of a ball being hit. The present study provides evidence against this alter- native explanation by testing the effect of grunting in a sport—mixed martial arts—where distraction, rather than masking, is the most likely mechanism. Methodology/Principal findings We first confirmed that kicking force is increased when a grunt is performed (Experiment 1), and then adapted methodology used in the tennis study to mixed martial arts (Experiment 2). Lifting the foot to kick is a silent act, and therefore there is nothing for a grunt to mask, i.e., its effect on an opponent's response time and/or accuracy can likely be attributed to attentional distraction. Participants viewed videos of a trained mixed martial artist kicking that included, or did not include, a simulated grunt. The task was to determine as quickly as possible whether the kick was traveling upward or downward. Overall, and replicating the tennis finding, the present results indicate that a participant's response to a kick was delayed and more error prone when a simulated grunt was present. Conclusions/Significance The present findings indicate that simulated

		grunting may distract an opponent, leading to slower and more error prone responses. The implications for martial arts in particular, and the broader question of whether grunting should be perceived as ’cheating’ in sports, are examined.
Stephenson&Rossheim (2018)	No	Limited research has systematically examined injuries resulting from Brazilian JiuJitsu (BJJ), Judo, and mixed martial arts (MMA), especially when compared to morewell-known or -established martial arts. These three combative sports differ substantially regarding their rules and techniques. BJJ emphasizes ground positioning and submission, Judo rewards throwing an opponent on their back with submission ending the match, and MMA emphasizes knocking out or forcing the submission ofone’s opponent. We examined injuries, among people of any age, experienced from participation in BJJ, Judo, and MMA. We analyzed data from the United States(U.S.) Consumer Product Safety Commission National Electronic Injury SurveillanceSystem to create estimates of injuries presenting to U.S. emergency departments(EDs). We compared injury profiles between sports, including estimatednumbers of injuries, their site, type, and mechanism. Participation in BJJ, Judo, andMMA resulted in an estimated 39,181 injuries presenting to U.S. EDs from 2008through 2015. Strains and sprains were the most common diagnoses for BJJ andJudo participants, whereas abrasions/contusions were the most commonly diagnosedMMA injury. Being struck resulted in the majority of injuries for all three sports.The head was the most injured body region for BJJ and MMA, whereas the leg wasthe most injured body region for Judo. Finally, the majority of BJJ and Judo injuriesoccurred during noncompetitive grappling, whereas most MMA injuries occurredduring

		competition. Our study adds to the limited literature examining injuries fromBJJ, Judo, and MMA using data from a probability sample and is an initial step towards understanding the national burden of injury from participation in thesesports. Given the quantity and severity of injuries sustained by participants, additional research is needed to assess the riskiness of participation and the effectiveness of interventions, such as improved personal protective gear and mats, as a means to prevent commonly occurring injuries.
Schild & Zettler., (2021)	No	Whereas voice pitch is strongly linked to people's perceptions in contexts of sexual selection, such as attractiveness and dominance, evidence that links voice pitch to actual behaviour or the formidability of a speaker is sparse and mixed. In this registered report, we investigated how male speakers' voice pitch is linked to fighting success in a dataset comprising 135 (amateur) mixed martial arts and 189 (ama- teur) boxing fights. Based on the assumption that voice pitch is an honest signal of formidability, we expected lower voice pitch to be linked to higher fighting success. The results indicated no significant rela- tion between a fighter's voice pitch, as directly measured before a fight, and successive fighting success in both mixed martial arts fighters and boxers.
Stellpflug, Menton & LeFevere.., (2020)	Yes	Objective: Knowledge of sportive chokes is vital to the practice of Sports Medicine when providing care at mixed martial arts and submission grappling events. This is a descriptive analysis of fight-ending chokes to help provide data on the topic not previously presented. Methods: An analysis was done on every fight-ending choke in the history of the UFCTM mixed martial arts promotion. Investigators focused on the frequency of chokes, types of chokes, handedness of the chokes, and whether chokes resulted in loss of consciousness. This analysis was done using existing fight outcome reports and video analysis of every choke that ended a fight in UFCTM history. Results: During the study period there were 904 such chokes, comprising 15.5% of fight outcomes and

		76.2% of grappling submissions. The makeup of right (50.1%) and left (49.9%) handedness of the chokes has been essentially identical ($\chi2$ [1] = 0.0011, p = .947, phi = .00). Most of the fight-ending chokes culminated in voluntary submission, however 11% resulted in loss of consciousness. The rear naked choke (RNC) was significantly more frequent than other chokes, comprising 49.1% of the total choke finishes; 19 other choke types accounted for the remaining 50.9%. Conclusion: Fight-ending chokes have been common in MMA. Many types of chokes have successfully ended UFCTM fights, with the RNC accounting for almost half of fight-ending chokes. Loss of consciousness occurred in 11% of fight-ending chokes. Right and left handed chokes were utilized equally.
Tabbenet al., (2018)	Yes	Objective: The aim of the present study was to examine the effect of Cold WaterImmersion (CWI) on the recovery of physical performance, hematological stress markersand perceived wellness (i.e., Hooper scores) following a simulated Mixed Martial Arts(MMA) competition.Methods: Participants completed two experimental sessions in a counter-balanced order (CWI or passive recovery for control condition: CON), after a simulated MMAscompetition (3 _ 5-min MMA rounds separated by 1-min of passive rest). During CWI,athletes were required to submerge their bodies, except the trunk, neck and head,in the seated position in a temperature-controlled bath (_10_C) for 15-min. DuringCON, athletes were required to be in a seated position for 15-min in same room ambient temperature. Venous blood samples (creatine kinase, cortisol, and testosteroneconcentrations) were collected at rest (PRE-EX, i.e., before MMAs), immediately following MMAs (POST-EX), immediately following recovery (POST-R) and 24 h postMMAs (POST-24), whilst physical fitness (squat jump, countermovement-jump and 5-and 10-m sprints) and perceptual measures (well-being Hooper index: fatigue, stress,delayed onset muscle soreness (DOMS), and sleep) were collected at PRE-EX, POST-Rand POST-24, and at PRE-EX and POST-24, respectively. Results: The main results indicate that POST-R sprint (5- and

		10-m) performanceswere 'likely to very likely' (d = 0.64 and 0.65) impaired by prior CWI. However, moderateimprovements were in 10-m sprint performance were 'likely' evident at POST-24 afterCWI compared with CON (d = 0.53). Additionally, the use of CWI 'almost certainly'resulted in a large overall improvement in Hooper scores (d = 1.93). Specifically, CWI'almost certainly' resulted in improved sleep quality (d = 1.36), stress (d = 1.56) andperceived fatigue (d = 1.51), and 'likely' resulted in a moderate decrease in DOMS(d = 0.60). Conclusion: The use of CWI resulted in an enhanced recovery of 10-m sprintperformance, as well as improved perceived wellness 24-h following simulated MMA competition.
Tiernan et al., (2021)	Yes	Thirteen MMA athletes were fitted with the MiG2.0 Stanford instrumented mouthguard. 451 video confirmed impacts were recoded during sparring sessions and competitive events. The competitive events resulted in five concussions. The impact with the highest angular acceler- ation from each event was simulated using the GHBMC head model. Average strain in the cor- pus callosum of concussed fighters was 0.27, which was 87.9% higher than uninjured fighters and was the best strain indicator of concussion. The best overall predictor of concussion found in this study was shear stress in the corpus callosum which differed by 111.4% between con- cussed and uninjured athletes
Tota et al., (2019)	No	The aim of the study was: 1. to evaluate the effects of conditioning training on body build and physical fitnessin elite mixed martial athletes, 2. to investigate the training load structure and assess body build as well as thephysiological profile of mixed martial arts athletes. Fifteen MMA male athletes (body mass: 79.8 ± 3.9 kg; body height:178.7 ± 7.9 cm; body fat: 13.4 ± 1.6%) were involved in the study. The average training experience of athletes equalled11 ± 1.1 years. Body composition, upper limb peak anaerobic power and aerobic performance were assessed before andafter the preparatory phase. During each evaluation, athletes underwent two stress tests: the Wingate test for the upperlimbs (upper

		limb anaerobic peak power measurement) and the graded exercise test until volitional exhaustion(maximal oxygen uptake measurement and second ventilatory threshold determination). Training means wereinvestigated for the workload type, intensity and exercise metabolism. In the follow-up, bodyfat mass decreased, whileanaerobic peak power and aerobic performance improved. Improvement in the time to obtain and maintain peak powering the upper limbs was noted. Training periodization resulted in advantageous body composition changes and improvedphysical fitness of the MMA athletes.
Wiechmann et al., (2016)	No	The aim of this paper is to identify predictorsof serum muscle damage marker (MDM)response following mixed martial arts (MMA)matches. Creatine kinase activity (CK) andmyoglobin concentration (Mb) were measured in ten male elite MMA fighters (aged 28±5.7years) prior to, 2 h, 24 h, and 96 h following 9different MMA matches. The number of performedupright punches and kicks (UKF) thatfailed the opponent, the number of obtained hits to the upper and lower body (LBH), as wellas the total fight duration (TFD) were evaluated as potential predictors from video recordings.CK peaked 24 h (829±753 U/L-1) and Mbpeaked 2 h (210±122 μg/L-1) post matches.Almost 80% of the peak CK variance could beexplained by LBH and UKF, whereas 87% of theMb variation was explained by TFD and LBH.MMA result in a significant skeletal muscledamage, which largely depends on LBH.Furthermore, eccentric contractions to deceleratekicks that missed the opponent and the TFD seem to contribute to the MDM response.
Zebrowska et al., (2017)	No	Objective: Physical methods are reported to be important for accelerating skeletal muscle regeneration, decreasing musclesoreness, and shortening of the recovery time. The aim of the study was to assess the effect of the physical methods of lymphaticdrainage (PMLD) such as manual lymphatic drainage (MLD), the Bodyflow (BF) therapy, and lymphatic drainage by deep oscillation(DO) on postexercise regeneration of the forearm muscles of mixed martial arts (MMA) athletes. Design and

		Methods: EightyMMAathletes aged 27.566.4 years were allocated to 4 groups: MLD, the BF device,DOtherapy, and the control group. Blood flowvelocity in the cephalic vein was measured with the ultrasound Doppler velocity meter. Maximalstrength of the forearm muscles(Fmax), muscle tissue tension, pain threshold, blood lactate concentration (LA), and activity of creatine kinase were measured in allgroups at rest, after the muscle fatigue test (post-ex) and then 20 minutes, 24, and 48 hours after the application of PMLD.Results: The muscle fatigue test reduced Fmax in all subjects, but in the groups receiving MLD, DO, and BF significantly higherFmax was observed at recovery compared with post-ex values. The application of MDL reduced the postexercise blood LA andpostexercise muscle tension. Conclusions: The lymphatic drainage methods, whether manual or using electro-stimulation andDO, improve postexercise regeneration of the forearm muscles of MMA athletes. The methods can be an important element of therapeutic management focused on optimizing training effects and reducing the risk of injuries of the combat sports athletes.
